# Systematic Exploration of the Potential Material Basis and Molecular Mechanism of the Mongolian Medicine Shudage-4 in Attenuating Stress-Induced Gastric Ulcer in Rat

**DOI:** 10.1155/2023/8998368

**Published:** 2023-06-16

**Authors:** Xin Jia, Xiaoling Zhu, Siyuan Chen, Yuexuan Wang, Jing Liu, Tianlong Liu, Yu Dong

**Affiliations:** ^1^School of Chinese Materia Medica, Tianjin University of Traditional Chinese Medicine, Tianjin 301617, China; ^2^Department of Pharmacy, Affiliated Hospital of Inner Mongolia Medical University, Hohhot 010059, China; ^3^Engineering Technology Research Center of Pharmacodynamic Substance and Quality Control of Mongolian Medicine in Inner Mongolia, Hohhot 010110, China; ^4^Inner Mongolian International Mongolian Hospital, Wulanchabudong Street, Hohhot 010090, China; ^5^Department of Natural Medicinal Chemistry, College of Pharmacy, Inner Mongolia Medical University, Hohhot 010110, China; ^6^Key Laboratory of Clinical and Basic Research on Cardiovascular Diseases, Basic Research Team of Cardiovascular Diseases, Inner Mongolia Medical University, Hohhot 010110, China

## Abstract

Shudage-4, an ancient and well-known formula in traditional Mongolian medicine comprising four different types of traditional Chinese medicine, is widely used in the treatment of gastric ulcers. However, the potential material basis and molecular mechanism of Shudage-4 in attenuating stress-induced gastric ulcers remain unclear. This study aimed to first explore the potential material basis and molecular mechanism of Shudage-4 in attenuating gastric ulcers in rats. The chemical constituents and transitional components in the blood of Shudage-4 were identified by ultra-performance liquid chromatography time-of-flight mass spectrometry (UPLC-TOF-MS). The rat gastric ulcer model was induced by water immersion restraint stress (WIRS). The ulcer damage to gastric tissue was measured at the gross anatomical level and pathological level by hematoxylin-eosin (HE) staining of gastric tissue. RNA sequencing of gastric tissue and plasma metabolomics were performed to analyze the mechanism of Shudage-4 against gastric ulcers. A Pearson correlation analysis was performed to explore the association between serum metabolites and gene expression of gastric tissue. A total of 30 chemical constituents were identified in Shudage-4 by UPLC-TOF-MS. Among 30 constituents, 13 transitional components in the blood were considered as the potential material basis. Shudage-4 treatment had a significant effect on WIRS-induced gastric ulcers in rats. HE staining of gastric tissue illustrated that WIRS-induced ulcer damage was suppressed by Shudage-4 treatment. RNA sequencing of gastric tissue showed that 282 reversed expression genes in gastric tissue were related to Shudage-4 treatment, and gene set enrichment analysis revealed that Shudage-4 treatment significantly inhibited gene set expression related to reactive oxygen species (ROS), which was also validated by detecting rat gastric tissue MDA, GSH, SOD, GSH-Px, and CAT activities. The plasma metabolomic data demonstrated that 23 significantly differential metabolites were closely associated with the Shudage-4 treatment. The further multiomics joint analysis found that significantly upregulated 5 plasma metabolites in Shudage-4-treated rats compared to model rats were negatively correlated with gene set expression related to ROS in gastric tissue. Shudage-4 alleviated WIRS-induced gastric ulcers by inhibiting ROS generation, which was achieved by regulating plasma metabolites level.

## 1. Introduction

Gastric ulcers are a common disorder of the digestive system. The direct cause of its development is the disruption of the balance between the defense factors of the gastric mucosa (mucus, prostaglandins, HCO_3_, and so on) and the attack factors of the gastric mucosa (gastric acid, pepsin, and so on) [1, 2]. The prevalence of the disease is growing as habits and diets change [3]. Reactive oxidative species (ROS) are free radicals or nonradical-like products obtained by the reduction of oxygen molecules, including superoxide anion (O^2−^), hydroxyl radical (OH^−^), and hydrogen peroxide (H_2_O_2_) [4]. ROS are usually products of electron transfer reactions such as mitochondria and peroxisomes and can be scavenged by intracellular enzymatic or nonenzymatic antioxidant systems, including reduced glutathione (GSH), peroxidase, superoxide dismutase (SOD), malondialdehyde (MDA), superoxide dismutase (SOD), and malondialdehyde (MDA) [5, 6]. Under pathological conditions, oxidative stress occurs when the body's ROS levels increase or the cellular antioxidant capacity decreases. Some studies reported the association between increased ROS levels and the development of gastric ulcers [7]. Besides, it has been found that ROS as signaling molecules could damage local gastric tissue and lead to ulcer damage by promoting the proliferation and differentiation of B lymphocytes and monocyte macrophages in the gastric mucosa [8]. Currently, gastric ulcer is managed by inhibiting gastric acid secretion using chemical drugs, neutralizing the acid with antacid drugs, or impeding cellular apoptosis with cytoprotective drugs [9, 10]. However, most of these drugs have side effects such as joint pain, altered heartbeat, hemopoietic changes, gynecomastia, impotence, and systemic alkalosis.

Shudage-4 (also called “Chang Pu Si Wei,” 4 : 2 : 1 : 1), an ancient and classic formula in traditional Mongolian medicine consists of *Alpinia officinarum* Hance., *Halite Violaceous*, *Aucklandia lappa* Decne., and *Acrorus tatarinowii* Schott., is commonly used in Mongolian medicine to treat a variety of symptoms such as indigestion, chest tightness and shortness of breath, diaphragmatic spasm, and stabbing pain in the chest and ribs. In the clinical practice of Mongolian medicine, Shudage-4 is commonly used in the treatment of gastric ulcers and is highly effective; however, the pharmacological basis and mechanism of Shudage-4 for the treatment of diseases are not clarified. A variety of chemical components are reported in Shudage-4, including flavonoids, lactones, phenylpropanoids, and volatile oils. Especially for flavonoids, kaempferol, galangin, kaempferitin, and galanin-3-methyl ether were isolated from Shudage-4 in our labs. This study aimed to investigate the mechanism of protection against gastric ulcers by Shudage-4 using multiomics data.

## 2. Materials and Methods

### 2.1. Animals and Ethics Statement

Six-week-old male Wistar rats (200–220 g) were obtained from the Laboratory Animal Centre of Inner Mongolia University (Licence ID : SCXK2016-0001, Hohhot, China). The animals were housed under a 12 h light/dark cycle at 23–25°C in a 40–70% relative humidity environment. All animals had free access to water and food during the experimental periods. The animals were acclimatized to the laboratory conditions for a week before the experiments. The animal experiments were performed by the Principles of Laboratory Animal Care and were approved by the ethical committee of experimental animal care at Inner Mongolia Medical University (NO. YKD202001061) on April 7th, 2020.

### 2.2. Induction of Gastric Ulcers and Treatment

As described previously, acute gastric ulcers were induced by water immersion restraint stress in a rat model [3]. Rats were immobilized in unique individual cages, and we immersed these animals in water at 22–24°C for 24 h to the level of the xiphoid process (water immersion and restraint stress). After exposure to stress, rats were weighed and anesthetized by intraperitoneal injections of 0.3% sodium pentobarbital (0.3 mL/100 g). Blood samples were collected from the abdominal aorta using filling K (2) EDTA blood collection tubes to detect metabolomic characteristics, then the stomach was collected and prepared for histopathological studies to evaluate the protective effect of Shudage-4 on the gastric ulcer.

### 2.3. Preparation of Ethanolic Extract of Shudage-4

An alcoholic extract of Shudage-4 was prepared, and the extraction rate was 5.50%. Briefly, a total of 20 kg Shudage-4 (4 : 2 : 1 : 1, consists of *Alpinia officinarum* Hance., *Halite Violaceous*, *Aucklandia lappa* Decne., and *Acrorus tatarinowii Schott.*) was powdered and extracted with 50% (v/v) ethanol reflux extraction, extracted twice for 2 h each time, and then mixed with extraction solvent. The extraction solvent was concentrated by reduced-pressure distillation until alcohol-free; ethyl alcohol content in the extraction solvent was determined with an alcohol meter, and then was fixed to capacity with 1.5-fold volume distilled water. The solution was separated through an AB/8 macroporous adsorption resin column and deionized water was used as elution solution, then aqueous eluate was discarded. 50% ethanol solution (8-fold) was used as elution solution and collected elution solution. The solvent was concentrated to be alcohol-free, and ethyl alcohol content in elution solution was determined with alcohol meter. The elution solution then was evaporated to dryness to obtain the ethanolic extract of Shudage-4.

### 2.4. Gastric Ulcers Treatment

The ethanolic extract of Shudage-4 was dissolved with 0.5% CMC-Na to prepare a suspension solution used to treat rats. According to body surface area conversion equations (human : rat = 1 : 6.3), the clinical dosage of Shudage-4 (400 g/70 kg) in adults is equal to the daily dosage of 105 mg/kg/d in rats. The rats in the high-, middle-, and low-dose Shudage-4-treated groups received 315 mg/kg, 105 mg/kg, and 35 mg/kg Shudage-4 treatment *per* day by gavage, respectively. Fifty rats were randomly assigned to the vehicle (*n* = 10), gastric ulcers (*n* = 10), and Shudage-4-treated (high, middle, and low dose, *n* = 10 *per* group) groups. Rats received a two-week pretreatment before being exposed to stress. The rats in the Shudage-4-treated groups received daily high-, middle-, and low-dose Shudage-4 *per* day by gavage, whereas the rats in the vehicle and gastric ulcers groups got the same volume of 0.5% CMC-Na daily by gavage. After pretreatment, rats in the gastric ulcers and Shudage-4-treated groups underwent water immersion restraint stress.

### 2.5. Identify Chemical Composition, the Compositions Absorbed into the Rat Blood Plasma and Gastric Tissue of Shudage-4 by UPLC-Q-TOF-MS

15 mg ethanolic extract of Shudage-4 was dissolved to 15 mL with 50% acetonitrile in water, and then the chemical composition of Shudage-4 was identified by UPLC-Q-TOF-MS. The normal rats were treated with a 10-fold dosage of Shudage-4 by gavage for 30 min and then were anesthetized by intraperitoneal injections of 0.3% sodium pentobarbital (0.3 mL/100 g). Blood samples were collected from the abdominal aorta using filling K (2) EDTA blood collection tubes, and gastric tissue was collected. Blood samples were centrifuged at 1000 × g for 10 min to obtain plasma. 4 mL acetonitrile was added to 1 mL plasma to prepare a mixed solution, and then was centrifuged at 1000 × g for 10 min. The supernatant was evaporated to dryness with centrifugal vacuum concentrators. The solid was dissolved with 200 *μ*L water containing 50% acetonitrile and then was centrifuged at 1000 × g for 10 min to obtain the supernatant. After the supernatant was filtered with 0.22 *μ*m microporous membrane, the compositions absorbed into the rat blood plasma of Shudage-4 were detected by UPLC-Q-TOF-MS.

The gastric tissue homogenate was prepared with a roller-ball crusher; 10 mL homogenate mixed with 4 mL acetonitrile, and then was centrifuged at 1000 × g for 10 min to obtain the supernatant. The supernatant was evaporated to dryness with centrifugal vacuum concentrators. The solid was dissolved with 200 *μ*L water containing 50% acetonitrile, and then was centrifuged at 1000 × g for 10 min to obtain supernatant. After the supernatant was filtered with 0.22 µm microporous membrane, the compositions absorbed into the gastric tissue of Shudage-4 were detected by UPLC-Q-TOF-MS.

### 2.6. Chromatography/Mass Spectrometry Conditions

The chemical composition of Shudage-4 was identified by UPLC-Q-TOF-MS (Agilent 1290 InfinityII-6546, USA) under positive and negative ion modes as following chromatography/mass spectrometry conditions, the HPLC system (1290, Agilent, USA) comprised an EC-C18 (2.1 × 10 mm, 1.8 *μ*m) with a column temperature of 40°C. The gradient elution used 0.1% carbolic acid in water (solution A) and 0.1% carbolic acids in acetonitrile (solution B), the gradient program is shown in Supplementary Table S1. The mass spectrometer parameters were source injection voltages of 4000 V (+) and 3500 V (−), an ion source temperature of 120°C, desolvation temperature of 300°C, collision energy of 3.5 eV, extraction cone voltage of 4.0 V, source temperature of 120°C, cone gas flow of 5 L/h and desolvation gas of 11 L/h. Data were centroided and mass was corrected during acquisition using an internal reference infused at a flow rate of 50 *μ*L/min via a lock spray interface, generating a real-time reference ion of [M + H] + (121.0508 Da) for positive and negative ion modes of [M − H] − (112.9855 Da) to ensure accurate MS analysis. All data collected in centroid mode were obtained and used to calculate the accurate mass and composition of the relative target ions with Qualitative Analysis (V10.0) software.

### 2.7. Gastric Ulcers and Pathological Tests

The pathological test was performed according to previous methods [11]. At the end of the experiment, the rats were anesthetized with 0.3% sodium pentobarbital (0.3 mL//100 g body weight), and the rat's stomach tissue was harvested. The gross photographs of gastric tissue were taken by camera (700 D, Canon, Japan) to evaluate the level of ulcer damage. The area of the gastric ulcer was counted using ImageJ software after the stomach tissue was photographed. The stomach tissue was immersed in paraffin and divided into 5 m slices after being fixed in 4% paraformaldehyde for 48 hours. To assess the level of pathological alteration, successive stomach tissue sections were stained using a HE staining kit (C0105S, Abcam, Beyotime, Shanghai, China). Tissue sections were photographed with a microscope (DM2000, Leica, Germany) for histopathological evaluation according to a previous study [12].

### 2.8. RNA Sequencing of Rat Gastric Tissue

To clarify the protective mechanisms of Shudage-4 on gastric ulcers, the rat stomach tissue was chosen to perform RNA sequencing, and there were five replicates per group. Total RNA was extracted using a TRIzol reagent according to the manufacturer's protocol. RNA purity and quantification were evaluated using a NanoDrop 2000 spectrophotometer (Thermo Scientific, USA). RNA integrity was assessed using an Agilent 2100 Bioanalyzer (Agilent Technologies, Santa Clara, CA, USA). Then, libraries were constructed using the TruSeq Stranded mRNA LT Sample Prep Kit (Illumina, San Diego, CA, USA) according to the manufacturer's instructions. Applied Protein Technology Co., Ltd. (Shanghai, China) conducted transcriptome sequencing and analysis.

### 2.9. Plasma Untargeted Metabolomics

After exposure to stress for 24 h, the rats were anesthetized with 0.3% sodium pentobarbital (0.3 mL/100 g body weight), and blood samples were collected from the abdominal aorta using filling K (2) EDTA blood collection tubes. The blood samples were centrifuged at 1000 × g for 10 min to isolate plasma. Plasma untargeted metabolomics was conducted by Applied Protein Technology Co., Ltd. (Shanghai, China). The raw MS data (whiff. scan files) were converted to MzXML files using ProteoWizard MSConvert before they were imported into freely available XCMS software.

### 2.10. Rat Gastric Tissue MDA, GSH, SOD, GSH-Px, and CAT Activities Detection

Plasma malondialdehyde (MDA), reduced glutathione (GSH), superoxide dismutase (SOD), glutathione peroxidase (GSH-Px), and catalase (CAT) activities were detected by the MAD Assay Kit (S0131S, Beyotime Bioengineering Institute, Shanghai, China), GSH/GSH-Px Assay Kit (S0053, Beyotime Bioengineering Institute, Shanghai, China), SOD Assay Kit (S0101S, Beyotime, Shanghai, China) and CAT Assay Kit (S0051, Beyotime, Shanghai, China), respectively.

### 2.11. Statistical Analysis

The mean ± standard deviation (SD) was calculated for all data. Statistical differences were calculated with the two-tailed Student's *t*-test when comparing two conditions and an ANOVA was used when comparing more than two conditions. For parametric data with equal variance, one-way ANOVA with the Tukey's post hoc test was used. For parametric data with unequal variance, one-way ANOVA with the Games–Howell post hoc test was used. Pearson correlation analysis, principal component analysis (PCA), and Gene Set Enrichment Analysis (GSEA) were performed using R version 3.5.2 (2018–12-20) (R Core Team 2018, 2018, The R Foundation for Statistical Computing, Vienna, Austria). A *pvalue* <0.05 was considered statistically significant.

## 3. Results

### 3.1. The Potential Material Basis of Shudage-4 in Improving Gastric Ulcers

**Figure 1:**
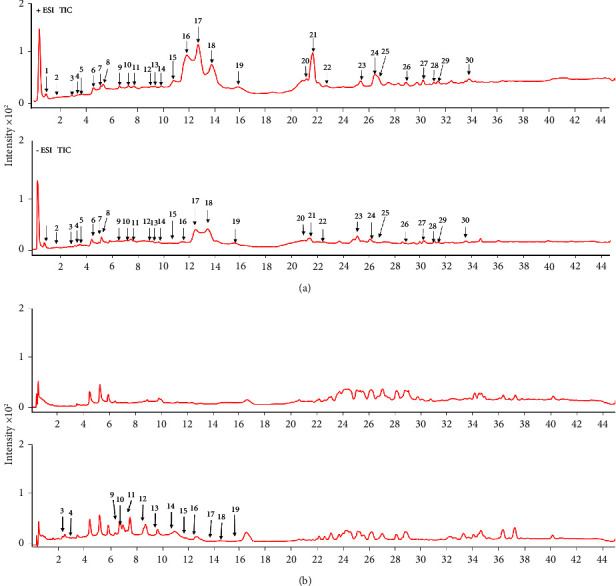
The potential material basis of Shudage-4 in improving gastric ulcers. (a) Total ion chromatogram of ethanolic extract of Shudage-4 under positive (up) and negative ion (down) mode and (b) Total ion chromatogram of blank plasma (up) and drug containing plasma (down).

To study the material basis of Shudage-4, the constituents were first identified in ethanolic extracts of Shudage-4 by UPLC-Q-TOF-MS under positive and negative ion modes. The total ion chromatogram is shown in Figure 1(a). The ion peaks in the chromatogram were further analyzed by Qualitative Analysis 10.0 software (Agilent, USA) to obtain direct fragmentation information. Finally, 30 constituents in ethanolic extracts of Shudage-4 were identified, which are shown in Supplementary Table S2. Further analysis showed that 13 constituents absorbed into the blood of Shudage-4 were found by UPLC-Q-TOF-MS, which are displayed in Figure 1(b) and Supplementary Table S3.

### 3.2. Protective Effect of Shudage-4 on Water Immersion Restraint Stress-Induced Gastric Ulcers

**Figure 2:**
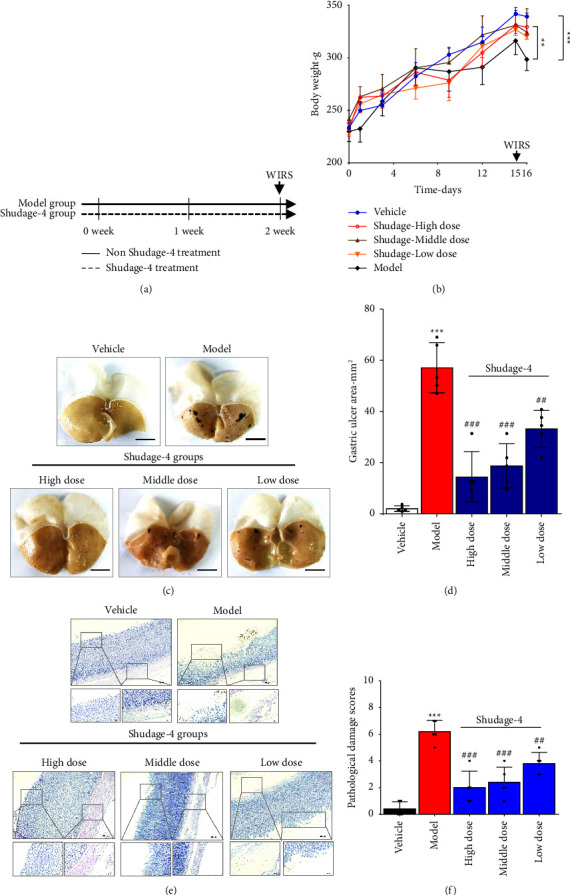
Protective effect of Shudage-4 on water immersion restraint stress-induced gastric ulcers. (a) Overall experimental design; (b) rat body weight change; (c) representative images of the gross gastric tissue (scale bar, 1 cm); (d) quantification of the gastric ulcers in different groups (*n* = 5); (e) hematoxylin and eosin-stained gastric tissue (scale bar, 40 *μ*m, 20x); (f) quantification of the gastric damage in different groups (*n* = 5). Data are presented as the mean ± SD based on a one-way ANOVA with Bonferroni post hoc test, compared to vehicle, ^*∗∗∗*^*P* < 0.001; compared to model, ^##^*P* < 0.01, ^###^*P* < 0.001.

The protective effect of Shudage-4 on gastric ulcers was validated according to a predetermined experimental design (Figure 2(a)). The results illustrated that Shudage-4 had a significant protective effect on the WIRS-induced decrease in rat body weight (Figure 2(b)). After exposure to stress for 24 h, rat stomach tissue appeared obvious ulcers and local hemorrhage in the glandular part of the stomach, while the area of the ulcer and hemorrhage was significantly less in the stomach tissue of Shudage-4-treated rats than in model groups (Figures 2(c) and 2(d)). Compared to vehicle rats, the intact structure of the gastric mucosa was changed in model group, which were showed the disruption in the glandular epithelium with erosion, edema of submucosa, while Shudage-4-treated rats improved these pathological injury, and showed less mucosal damage and milder edema compared with model rats (Figures 2(e) and 2(f)).

### 3.3. RNA Sequencing Highlights Shudage-4 Improved Gastric Ulcers by Inhibiting Water Immersion Restraint Stress-Induced Oxidative Stress

**Figure 3:**
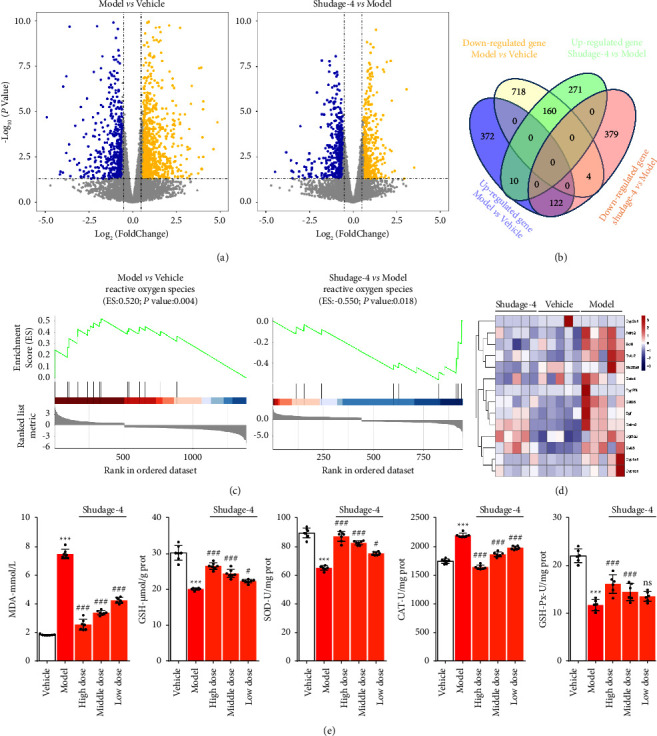
RNA sequencing highlights Shudage-4 improved gastric ulcers by inhibiting water immersion restraint stress-induced oxidative stress. (a) Volcano plot visualizing differential expression of the genes of the gastric tissue. (b) An overlap was performed between upregulated and downregulated genes in different groups. (c) Gene set enrichment analysis (GSEA) results of significantly expressional genes. (d) The gene set involved in reactive oxygen species was enriched in different groups. (e) The markers of reactive oxygen stress were detected in different groups (*n* = 6). Data are presented as the mean ± SD based on a one-way ANOVA with Bonferroni post hoc test, compared to vehicle, ^*∗∗∗*^*P* < 0.001; compared to model, ^#^*P* < 0.05, ^###^*P* < 0.001, ns: no significant difference.

To gain a greater understanding of the protection of Shudage-4 on gastric ulcers, RNA sequencing of rat gastric tissue was performed. For RNA sequencing, fold changes >1.5 or <0.67 in gene expression among the different groups and *p* values <0.05 were considered statistically significant. Compared to the stomach tissue of Vehicle rats, 504 and 882 differentially expressed genes were significantly upregulated and downregulated in the rat stomach tissue of the gastric ulcers group, respectively. Compared with the rat stomach tissue of the gastric ulcers group, 441 and 505 differentially expressed genes were found in the rat stomach tissue of the Shudage-4-treated group (Figure 3(a)). Venn diagram showed that the protective effect of Shudage-4 on gastric ulcers was related to reverse water immersion restraint stress-induced 282 gene expression in stomach tissue (Figure 3(b)). A gene set enrichment analysis (GSEA) of the differentially expressed genes was performed using R software. The results highlighted those Shudage-4 improved gastric ulcers by inhibiting water immersion restraint stress-induced oxidative stress (Figure 3(c)). The expression level of the gene set involved in oxidative stress was displayed in Figure 3(d). To validate the results of RNA sequencing, MDA, GSH, SOD, GSH-Px, and CAT activities as oxidative stress-related proteins were detected in rat gastric tissue. Consistent with RNA sequencing results, a significant increase in the activities of MDA and CAT, and a decrease in the activities of GSH, SOD, and GSH-Px were found in rat gastric tissue of the gastric ulcers group compared to the vehicle group, while those were inhibited in rat gastric tissue of Shudage-4 treated group compared to the gastric ulcers group (Figure 3(e)). In summary, those results illustrated those Shudage-4 improved gastric ulcers by inhibiting water immersion restraint stress-induced oxidative stress.

### 3.4. Nontargeted Metabolomics Screened for Plasma Metabolites Regulated by Shudage-4

**Figure 4:**
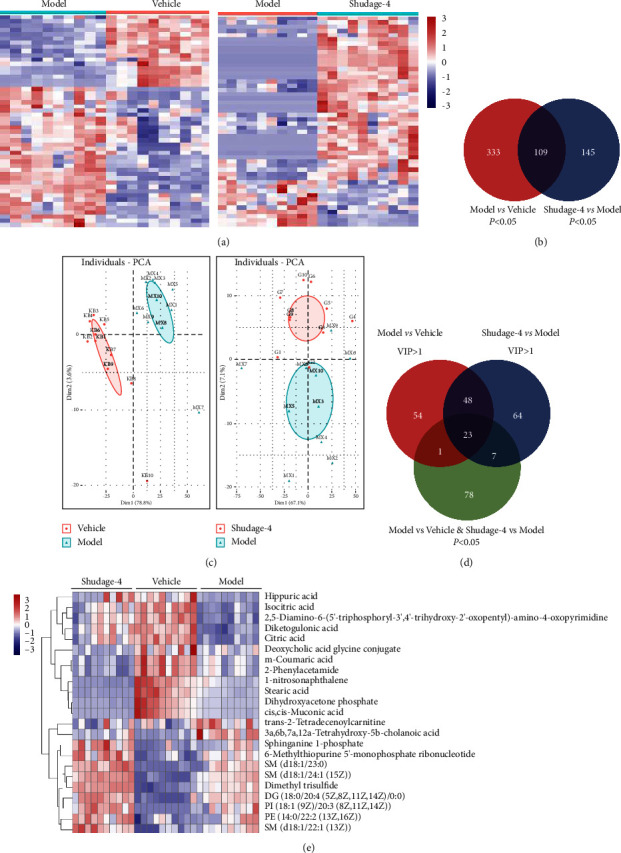
Plasma untargeted metabolomics highlights the protective mechanism of Shudage-4 on gastric ulcers. (a) The heatmap of plasma metabolite level in different groups (top 50); (b) an overlap of significant differential metabolites was performed among different groups; (c) principal component analysis (PCA) results of plasma untargeted metabolomics; (d) an overlap was performed between significantly differential metabolites and VIP value > 1 metabolites; (e) the heatmap of 23 plasma metabolites related to Shudage-4 treatment in different groups.

A total of 1155 metabolites were identified in rat plasma by performing untargeted metabolomics in positive and negative modes (Supplementary Table S4). The *p* values <0.05 among the different groups were considered statistically significant. Four hundred and forty-two metabolites had significant differences between vehicle and model rats, while those were 254 metabolites between Shudage-4-treated and model rats (Figure 4(a) and Supplementary Tables S5 and S6). Venn diagram showed that 109 significantly different metabolites were found among groups (Figure 4(b)). PCA score plots revealed that the Shudage-4-treated group was separated from the model and vehicle groups (Figure 4(c)). Finally, 23 metabolites with variable importance on projection (VIP) > 1 and *p* value <0.05 were found among the different groups (Figures 4(d) and 4(e)). In summary, plasma nontargeted metabolomics showed that the protective effect of Shudage-4 on WIRS-induced gastric ulcers was closely associated with regulating plasma 23 metabolites.

### 3.5. Multiomics Analysis Highlighted the Protective Mechanism of Shudage-4 on Gastric Ulcers

**Figure 5:**
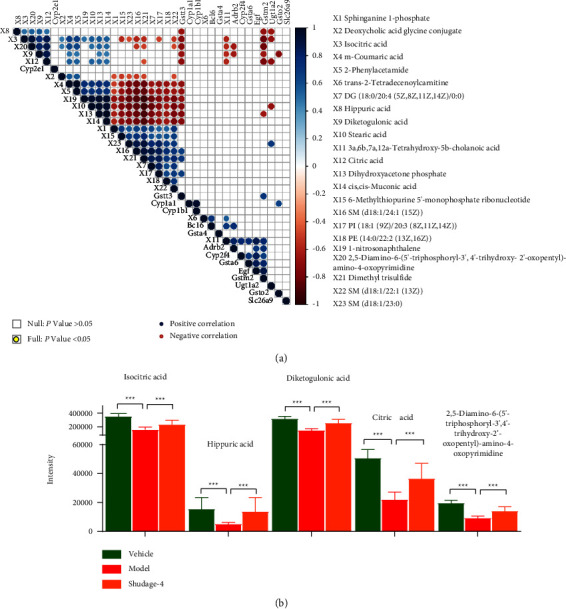
Multiomics analysis highlighted the protective mechanism of Shudage-4 on gastric ulcers. (a) Pearson correlations between metabolites related to Shudage-4 treatment and genes related to the reactive oxygen species in gastric tissue. (b) The levels of 5 metabolites related to Shudage-4 treatment were a negative correlation with gene expression related to the generation of reactive oxygen species in gastric tissue. Data are presented as the mean ± SD based on a one-way ANOVA with Bonferroni post hoc test, compared to vehicle, ^*∗∗∗*^*P* < 0.001.

To further investigate the protective mechanism of Shudage-4 on gastric ulcers, an integrative analysis of plasma nontargeted metabolomics and RNA sequencing of gastric tissue was performed. We calculated Pearson correlations between 23 metabolites related to Shudage-4 treatment and genes related to the generation of reactive oxygen species in gastric tissue. Among 23 metabolites, 5 upregulated metabolites in the plasma of the Shudage-4-treated rat were positively correlated with the expression level of at least one gene related to the generation of reactive oxygen species in gastric tissue (Figures 5(a) and 5(b)). Taken together, these findings provide direct evidence that Shudage-4 treatment inhibited the generation of reactive oxygen species in gastric tissue by regulating plasma metabolites to ameliorate WIRS-induced gastric ulcers.

## 4. Discussion

Shudage-4, an ancient and classic formula in traditional Mongolian medicine comprising four kinds of TCM, is widely used in the treatment of gastric ulcers. At present, there are limited publications on the biological basis and mechanism of Shudage-4 in improving stomach ulcers. In our study, 30 compositions were first identified in ethanolic extracts of Shudage-4 by UPLC-Q-TOF-MS. Among the 30 compositions, 13 constituents were absorbed into the rat blood plasma and gastric tissue. Multiomics analyses illustrated the protective effect of Shudage-4 on gastric ulcers related to inhibiting water immersion restraint stress-induced oxidative stress via regulating 5 metabolites in rat plasma.

Oxidative stress plays a critical role in the progression of gastric ulcers [13]. Reactive oxidative species (ROS) were mainly derived from mitochondrial dysfunction and released from infiltrating neutrophils under pathological conditions [14, 15]. Previous studies showed that ROS deteriorated gastric ulcers through multiple means. Excessive ROS reacted with lipids to cause lipid peroxidation, which was the main cause of structural and functional disorders of the gastric mucosal cells [16, 17]. Besides, ROS can influence the synthesis and release of matrix metalloproteinases (MMPs), while MMPs have been suggested to play a crucial role in the progression of gastric ulcers [18]. Under physiological conditions, gastric epithelial cells, macrophages, and neutrophils could generate and secrete MMPs, those MMPs and tissue inhibitors of metalloproteinases (TIMPs) worked together to maintain extracellular matrix (ECM) function in gastric tissues [19, 20]. It had been reported that excessive ROS disrupted the balance of protease-antiprotease action leading to ECM remodeling, which was closely associated with the pathogenesis of gastric ulcers [21]. Moreover, previous studies demonstrated that scavenging excessive ROS was considered a potential therapeutic approach for gastric ulcers [22, 23]. In our study, RNA sequencing of gastric tissue illustrated that Shudage-4 treatment could protect water immersion restraint stress-induced gastric ulcers via inhibiting expression of gene sets related to ROS.

Shudage-4 (also called “Chang Pu Si Wei,” 4 : 2 : 1 : 1), an ancient and classic formula in traditional Mongolian medicine, consists of *Alpinia officinarum* Hance, *Halite Violaceous*, *Auckland lappa* Decne., *Acorus tatarinowii* Schott. and *Alpinia officinarum* Hance. as an aromatic rhizome, used for many years in Chinese traditional medicine (TCM), has multiple pharmacological efficacies, such as anticancer, antiinflammation, and antioxidation [24]. Except for medicinal uses, *Alpinia officinarum* Hance native to Chine also been used as a spice in Europe for over 1000 years. Nearly 100 compounds were isolated from various parts of *Alpinia officinarum* Hance., mainly including diarylheptanoid, glycosides, flavonoids, and sesquiterpene [25]. Among compounds, there is sufficient evidence that flavonoids could improve multiple diseases via suppressed ROS [26]. *Aucklandia lappa* Dence. (Chinese trade name: Muxiang), the dried root of Radix Aucklandiae, has various kinds of bioactivities, including anti-inflammatory, antiulcer, hepatoprotective, sialagogic, antitumor and other functions [27]. Therefore, *Auckland lappa* Dence. is widely used in TCM or folk medicine for the treatment of abdominal distention, vomiting, diarrhea, and dysentery tenesmus [28]. YANG X's study showed that the extract of *Auckland lappa* Decne. exhibited a marked effect on animal peptic ulcer activity [29]. *Acorus tatarinowii* Schott. (Chinese trade name: Shi Chang Pu) belongs to the *Araceae* family and is used clinically in the treatment of dementia, learning and memory loss, depressant, convulsant, epileptic, and digestive diseases [30]. Some lignans and neolignans, volatile oils, polysaccharides, and alkaloids as main chemical compositions of *Acorus tatarinowii* Schott were associated closely with its bioactivities, especially for antioxidative stress [31–34].

In our study, the multiomics analysis showed that Shudage-4 has a significant protective effect on stress-induced gastric ulcers by inhibiting the expression of gene set related to ROS in gastric tissue, which was achieved via regulating 4 plasma metabolites level. Isocitric acid as a key metabolite of the tricarboxylic acid cycle possessed antioxidant properties [35]. Hippuric acid is a cometabolite generated by a variety of gut microorganisms from the digestion of plant polyphenols and aromatic amino acids [36]. Some studies showed that low hippuric acid levels in blood and urine were found in age-related illnesses such as sarcopenia, hypomobility, and cognitive impairment [37, 38]. Besides, decreased levels of hippuric acid in plasma have been related to type 2 diabetes and obesity [39]. Our results firstly found that the hippuric acid level was significantly decreased in the plasma of gastric ulcer rats compared to normal rats, while its downregulation was reduced in plasma after the treatment with Shudage-4. On the one hand, the change in plasma hippuric acid level of Shudage-4-treated rats is attributed to polyphenols supplementation from Shudage-4; on the other hand, the effect of Shudage-4 on intestinal flora promotes the generation of hippuric acid. Citric acid, produced in the tricarboxylic acid (TCA) cycle, plays a critical role in maintaining blood pH levels, suppressing the excess accumulation of blood lactate, and promoting energy production [40]. A clinical study reported that the intake of citric acid alleviated fatigue in daily activities [41]. Moreover, K Marazova's study reported that MX1 (a novel salt of the active metabolite of roxatidine with a complex of bismuth and citric acid) is significantly protective against stress-induced gastric ulcers in rats [42]. In summary, those studies provided a theoretical basis for elucidating the mechanism of Shudage-4 against stress-induced gastric ulcers.

## Data Availability

Except for supplementary data, other original data that used to support the findings of this study are available from the corresponding author upon reasonable request.

## Abbreviations

CAT: Catalase
CMC-Na: Sodium carboxymethylcellulose
ECM: Extracellular matrix
GSEA: Gene set enrichment analysis
GSH: Reduced glutathione
GSH-Px: Glutathione peroxidase
HE: Hematoxylin-eosin
MDA: Malondialdehyde
MMPs: Matrix metalloproteinases
PCA: Principal component analysis
ROS: Reactive oxygen species
SOD: Superoxide dismutase
TCM: Traditional Chinese medicine
TIMPs: Metalloproteinases
UPLC-TOF-MS: Ultra-performance liquid chromatography time-of-flight mass spectrometry
WIRS: Water immersion restraint stress.

## Supplementary Materials

Supplementary Materials Table S1: The elution gradient. Table S2: Mass spectrum information of compounds of Shudage-4. Table S3: Information table of the chemical composition of Shudage-4 in plasma. Table S4: 1155 metabolites were identified in rat plasma. Table S5: Significantly differential metabolites in rat plasma between model group and vehicle groups. Table S6: Significantly differential metabolites in rat plasma between Shudage-4 group and model groups.

## References

[1] Li W., Wang X., Zhi W. (2017). The gastroprotective effect of nobiletin against ethanol-induced acute gastric lesions in mice: impact on oxidative stress and inflammation. *Immunopharmacology and Immunotoxicology*.

[2] Bi W. P., Man H. B., Man M. Q. (2014). Efficacy and safety of herbal medicines in treating gastric ulcer: a review. *World Journal of Gastroenterology*.

[3] Dembinski A., Warzecha Z., Ceranowicz P. (2005). Role of capsaicin-sensitive nerves and histamine H1, H2, and H3 receptors in the gastroprotective effect of histamine against stress ulcers in rats. *European Journal of Pharmacology*.

[4] Ji Y., Yin W., Liang Y., Sun L., Yin Y., Zhang W. (2020). Anti-inflammatory and anti-oxidative activity of indole-3-acetic acid involves induction of HO-1 and neutralization of free radicals in RAW264.7 cells. *International Journal of Molecular Sciences*.

[5] Qiu W., He Y., Li L., Liu Z., Zhong S., Yu Y. (2021). Donor-acceptor pairs in covalent organic frameworks promoting electron transfer for metal-free photocatalytic organic synthesis. *Langmuir*.

[6] Limon-Pacheco J., Gonsebatt M. E. (2009). The role of antioxidants and antioxidant-related enzymes in protective responses to environmentally induced oxidative stress. *Mutation Research, Genetic Toxicology and Environmental Mutagenesis*.

[7] Yamasaki R., Dekio S., Jidoi J. (1985). Contact dermatitis from grape bud. *Contact Dermatitis*.

[8] Mittal M., Siddiqui M. R., Tran K., Reddy S. P., Malik A. B. (2014). Reactive oxygen species in inflammation and tissue injury. *Antioxidants and Redox Signaling*.

[9] Sontag S. J. (1997). Guilty as charged: bugs and drugs in gastric ulcer. *American Journal of Gastroenterology*.

[10] Fagundes F. L., Piffer G. D. M., Perico L. L., Rodrigues V. P., Hiruma-Lima C. A., dos Santos R. D. C. (2020). Chrysin modulates genes related to inflammation, tissue remodeling, and cell proliferation in the gastric ulcer healing. *International Journal of Molecular Sciences*.

[11] Yan T., Zhu X., Zhang X. (2022). The application of proteomics and metabolomics to reveal the molecular mechanism of Nutmeg-5 in ameliorating cardiac fibrosis following myocardial infarction. *Phytomedicine*.

[12] Lu S., Wu D., Sun G. (2019). Gastroprotective effects of Kangfuxin against water-immersion and restraint stress-induced gastric ulcer in rats: roles of antioxidation, anti-inflammation, and pro-survival. *Pharmaceutical Biology*.

[13] Singh L. P., Mishra A., Saha D., Swarnakar S. (2011). Doxycycline blocks gastric ulcer by regulating matrix metalloproteinase-2 activity and oxidative stress. *World Journal of Gastroenterology*.

[14] Bhatti J. S., Bhatti G. K., Reddy P. H. (2017). Mitochondrial dysfunction and oxidative stress in metabolic disorders a step towards mitochondria based therapeutic strategies. *Biochimica et Biophysica Acta Molecular Basis of Disease*.

[15] Lefer D. J., Granger D. N. (2000). Oxidative stress and cardiac disease. *The American Journal of Medicine*.

[16] Fan J., Xu G., Jiang T., Qin Y. (2012). Pharmacologic induction of heme oxygenase-1 plays a protective role in diabetic retinopathy in rats. *Investigative Ophthalmology & Visual Science*.

[17] Beiranvand M., Bahramikia S. (2020). Ameliorating and protective effects mesalazine on ethanol-induced gastric ulcers in experimental rats. *European Journal of Pharmacology*.

[18] Ganguly K., Kundu P., Banerjee A., Reiter R. J., Swarnakar S. (2006). Hydrogen peroxide-mediated downregulation of matrix metalloprotease-2 in indomethacin-induced acute gastric ulceration is blocked by melatonin and other antioxidants. *Free Radical Biology and Medicine*.

[19] Swarnakar S., Ganguly K., Kundu P., Banerjee A., Maity P., Sharma A. V. (2005). Curcumin regulates expression and activity of matrix metalloproteinases 9 and 2 during prevention and healing of indomethacin-induced gastric ulcer. *Journal of Biological Chemistry*.

[20] Ganguly K., Maity P., Reiter R. J., Swarnakar S. (2005). Effect of melatonin on secreted and induced matrix metalloproteinase 9 and 2 activity during prevention of indomethacin-induced gastric ulcer. *Journal of Pineal Research*.

[21] Ganguly K., Swarnakar S. (2009). Induction of matrix metalloproteinase 9 and 3 in nonsteroidal anti-inflammatory drug-induced acute gastric ulcers in mice: regulation by melatonin. *Journal of Pineal Research*.

[22] Dengiz G. O., Odabasoglu F., Halici Z., Suleyman H., Cadirci E., Bayir Y. (2007). Gastroprotective and antioxidant effects of amiodarone on indomethacin-induced gastric ulcers in rats. *Archives of Pharmacal Research*.

[23] Beiranvand M., Bahramikia S., Dezfoulian O. (2021). Evaluation of antioxidant and anti-ulcerogenic effects of Eremurus persicus (Jaub & Spach) Boiss leaf hydroalcoholic extract on ethanol-induced gastric ulcer in rats. *Inflammopharmacology*.

[24] Liu R., Li H., Wei N., Tan Y. (2021). Simultaneous determination of two galangin metabolites from Alpinia Officinarum Hance in rat plasma by UF LC-MS/MS and its application in pharmacokinetics study. *PeerJ*.

[25] Abubakar I. B., Malami I., Yahaya Y., Sule S. M. (2018). A review on the ethnomedicinal uses, phytochemistry and pharmacology of Alpinia officinarum Hance. *Journal of Ethnopharmacology*.

[26] Slika H., Mansour H., Wehbe N. (2022). Therapeutic potential of flavonoids in cancer: ROS-mediated mechanisms. *Biomedicine & Pharmacotherapy*.

[27] Cai X., Yang C., Qin G. (2022). Antimicrobial effects and active compounds of the root of Aucklandia lappa Decne (Radix Aucklandiae). *Frontiers in Chemistry*.

[28] Yuan Y., Hu Q., Liu L. (2022). Dehydrocostus lactone suppresses dextran sulfate sodium-induced colitis by targeting the ikk*α*/*β*-NF-*κ*b and keap1-nrf2 signalling pathways. *Frontiers in Pharmacology*.

[29] Yang X., Zhang X., Yang S. P., Le T., Chen B. (2016). Evaluation of Aucklandia lappa Decne extracts as antiulcer activity in animals. *Pakistan journal of pharmaceutical sciences*.

[30] Zhu M., Zhu H., Tan N. (2014). The effects of Acorus tatarinowii Schott on 5-HT concentrations, TPH2 and 5-HT1B expression in the dorsal raphe of exercised rats. *Journal of Ethnopharmacology*.

[31] Ni G., Shen Z. F., Lu Y. (2011). Glucokinase-activating sesquinlignans from the rhizomes of Acorus tatarinowii Schott. *Journal of Organic Chemistry*.

[32] Zhang Y., Long Y., Yu S. (2021). Natural volatile oils derived from herbal medicines: a promising therapy way for treating depressive disorder. *Pharmacological Research*.

[33] Zhong J., Qiu X., Yu Q., Chen H., Yan C. (2020). A novel polysaccharide from Acorus tatarinowii protects against LPS-induced neuroinflammation and neurotoxicity by inhibiting TLR4-mediated MyD88/NF-*κ*B and PI3K/Akt signaling pathways. *International Journal of Biological Macromolecules*.

[34] Faisal M., Shahzad D., Larik F. A., Dar P. (2018). Synthetic approaches to access acortatarins, shensongines and pollenopyrroside; potent antioxidative spiro-alkaloids with a naturally rare morpholine moiety. *Fitoterapia*.

[35] Morgunov I. G., Karpukhina O. V., Kamzolova S. V., Samoilenko V. A., Inozemtsev A. N. (2018). Investigation of the effect of biologically active threo-Ds-isocitric acid on oxidative stress in Paramecium caudatum. *Preparative Biochemistry & Biotechnology*.

[36] Li M., Wang B., Zhang M. (2008). Symbiotic gut microbes modulate human metabolic phenotypes. *Proceedings of the National Academy of Sciences of the U S A*.

[37] Kameda M., Teruya T., Yanagida M., Kondoh H. (2020). Frailty markers comprise blood metabolites involved in antioxidation, cognition, and mobility. *Proceedings of the National Academy of Sciences of the U S A*.

[38] Brunelli L., Davin A., Sestito G. (2021). Plasmatic hippuric acid as a hallmark of frailty in an Italian cohort: the mediation effect of fruit-vegetable intake. *The Journals of Gerontology: Series A*.

[39] Palau-Rodriguez M., Tulipani S., Isabel Queipo-Ortuno M., Urpi-Sarda M., Tinahones F. J., Andres-Lacueva C. (2015). Metabolomic insights into the intricate gut microbial-host interaction in the development of obesity and type 2 diabetes. *Frontiers in Microbiology*.

[40] Hara Y., Kume S., Kataoka Y., Watanabe N. (2021). Changes in TCA cycle and TCA cycle-related metabolites in plasma upon citric acid administration in rats. *Heliyon*.

[41] de Salles Painelli V., Lancha Junior A. H. (2018). Thirty years of investigation on the ergogenic effects of sodium citrate: is it time for a fresh start?. *British Journal of Sports Medicine*.

[42] Marazova K., Klouchek E., Popov A., Ivanov C. H., Krushkov I. (2011). Gastroprotective effect of MX1 (a novel salt of the active metabolite of roxatidine with a complex of bismuth and citric acid) against stress ulcers in rats. *Journal of Pharmacy and Pharmacology*.

