# Supplementary material for: Systematic Exploration of the Potential Material Basis and Molecular Mechanism of the Mongolian Medicine Shudage-4 in Attenuating Stress-Induced Gastric Ulcer in Rat
Source: Evid Based Complement Alternat Med. 2023 Jun 16;2023:8998368. doi: 10.1155/2023/8998368 (PMC10289874; doi:10.1155/2023/8998368)
Supplement: Supplementary Materials — Table S1: The elution gradient. Table S2: Mass spectrum information of compounds of Shudage-4. Table S3: Information table of the chemical composition of Shudage-4 in plasma. Table S4: 1155 metabolites were identified in rat plasma. Table S5: Significantly differential metabolites in rat plasma between model group and vehicle groups. Table S6: Significantly differential metabolites in rat plasma between Shudage-4 group and model groups. [file 8998368.f1.docx]

**Systematic exploration of the potential material basis and molecular mechanism of the Mongolian medicine Shudage-4 in attenuating stress-induced gastric ulcer in rat**

Xin Jia^a,b,c^, Xiaoling Zhu^d,c^, Siyuan Chen^e^ , Yuexuan Wang^e^, Jing Liu^a^, Tianlong Liu^a,f*^ and Yu Dong^e,c^*

**Author affiliations:**

**^a^** Department of Pharmacy, Affiliated Hospital of Inner Mongolia Medical University, Hohhot 010059, China.

**^b^** School of Chinese Materia Medica, Tianjin University of Traditional Chinese Medicine, Tianjin, 301617, China.

**^c^** Engineering Technology Research Center of Pharmacodynamic Substance and Quality Control of Mongolian Medicine in Inner Mongolia, Hohhot 010110, China

^d^ Inner Mongolian International Mongolian Hospital, Wulanchabudong Street, Hohhot 010090, China.

**^e^** Department of Natural Medicinal Chemistry, College of Pharmacy, Inner Mongolia Medical University, Hohhot 010110, China

^f^ Key Laboratory of Clinical and Basic Research on Cardiovascular Diseases, Basic Research Team of Cardiovascular Diseases, Inner Mongolia Medical University, Hohhot 010110, China

***Correspondence to:**

**Tianlong Liu, PhD**

Department of Pharmacy, Affiliated Hospital of Inner Mongolia Medical University, No. 1, Tongdao North Street, Huimin District, Hohhot 010059, PR China

E-mail: tianlongliu1984@163.com

Phone: Tel: +86-0471-3451657

**Yu Dong, PhD**

Department of Natural Medicinal Chemistry, College of Pharmacy, Research Center of Pharmacodynamic Substance and Quality Control of Mongolian Medicine in Inner Mongolia, Inner Mongolia Medical University, Jinshan Development Zone, Hohhot 010110, PR China.

E-mail: dongyu010@126.com

Phone: Tel: +86-13644887629

Running Title: Shudage-4 and gastric ulcer in rat

**Table S1 The elution gradient**

| Time (min) | A(0.1% carbolic acid in water)% | B(0.1% carbolic acid in acetonitrile)% |
| --- | --- | --- |
| 0.01 | 95 | 5 |
| 2 | 95 | 5 |
| 4 | 70 | 30 |
| 8 | 50 | 50 |
| 10 | 20 | 80 |
| 14 | 0 | 100 |
| 15 | 0 | 100 |
| 15.1 | 95 | 5 |
| 16 | 95 | 5 |

**Table S2 Mass spectrum information of compounds of Shudage-4**

| No. | tR/min | Identify | Formula | Mass | Fragment ions |
| --- | --- | --- | --- | --- | --- |
| 1 | 1.38 | P-Hydroxibenzoic acid | C_7_H_6_O_3_ | 138.0317 | 121 [M-OH]  95 [M-COO]- |
| 2 | 1.63 | Episyringaresinol 4'-O-β-D-glncopyranoside | C_28_H_36_O_13_ | 580.2155 | 580[M+]  418 [M-162] |
| 3 | 3.21 | 5-hydroxy-1-phenyl (4-hydroxy-7 -(4-hydroxy-5-methoxy phenyl) -3-heptanone | C_20_H_24_O_5_ | 344.1623 | 325 [M-H_2_O]-  149[M-195] |
| 4 | 3.47 | Pinobaksin | C_15_H_12_O_5_ | 272.0684 | 225[M-COOH]-  151[M-121]- |
| 5 | 3.91 | Cianidanol | C_15_H_14_O_6_ | 290.0793 | 290[M+]  271[M-H_2_O]- |
| 6 | 4.26 | Isorhamnetin | C_16_H_10_O_7_ | 314.0426 | 302[M-CH]+  271[M-COO]- |
| 7 | 4.54 | Luteolin | C_15_H_10_O_6_ | 286.0477 | 287[M+H]  213[M-CH]+ |
| 8 | 4.78 | 5-hydroxy-1-phenyl-7 -(3, 4-dihydroxyphenyl) -3-heptanone | C_19_H_22_O_4_ | 314.1518 | 285[M-CH]-  165[M-149] |
| 9 | 6.29 | Kaempferol | C_15_H_10_O_6_ | 286.0477 | 255[M-H_2_O]+  227[M-59] |
| 10 | 7.09 | Curcumin | C_21_H_20_O_6_ | 368.1259 | 351[M-OH]  323[M-COOH] |
| 11 | 7.81 | 3'-O-methylviolanone | C_18_H_18_O_6_ | 330.1103 | 314[M-OH]+  299[M-OCH_3_] |
| 12 | 8.22 | Quercetin | C_15_H_10_O_7_ | 302.0426 | 303[M+]  287[M-CH_3_] |
| 13 | 9.29 | 3，5-dihydroxy-1 -(4-hydroxy-3-methoxy phenyl) -7-phenylheptane | C_20_H_26_O_4_ | 330.1831 | 313[M-OH]  295[M-OH-H_2_O] |
| 14 | 10.09 | Pinocembrin | C_15_H_12_O_4_ | 256.0735 | 257[M+]  153[M-103] |
| 15 | 10.83 | 5-hydroxy-7 - (4-hydroxy-3-methoxy phenyl) -1-phenyl-3-he*p*tanone | C_20_H_24_O_4_ | 328.1674 | 310[M-OH]+  180[M-148] |
| 16 | 11.62 | Kaempferide | C_16_H_12_O_6_ | 300.0633 | 284[M-OH]+  227[M-73] |
| 17 | 12.46 | Galangin | C_15_H_10_O_5_ | 270.0528 | 269[M-]  242[M-CO] |
| 18 | 13.64 | Galangin-3-methyl ether | C_16_H_12_O_5_ | 284.0684 | 283[M+]  153[M-131] |
| 19 | 15.95 | 1,7-diphenyl-5-hydroxy-3-heptanone | [C_19_H_22_O_2_](https://pubchem.ncbi.nlm.nih.gov/#query=C19H22O2) | 282.1619 | 264[M-OH]-  159[M-123]- |
| 20 | 21.21 | Apigenin | C_15_H_10_O_5_ | 270.0528 | 271[M+]  153[M-47] |
| 21 | 21.55 | Diosmetin | C_16_H_12_O_6_ | 300.0633 | 286[M-CH_3_]-  271[M-CO]- |
| 22 | 22.63 | 2'-hydroxyformononetin | C_16_H_12_O_5_ | 283.0684 | 269[M-CH_3_]-  239[M-COO] |
| 23 | 25.64 | Hesperidin | C_28_H_34_O_15_ | 610.1897 | 609[M-]  301[M-309] |
| 24 | 26.53 | α-Asarone | C_12_H_16_O_3_ | 208.1099 | 208[M+]  193[M-CH_3_] |
| 25 | 26.53 | β-asarum | C_12_H_16_O_3_ | 208.1099 | 208[M+]  165[M-COO]- |
| 26 | 28.46 | Cedrenol | C_15_H_24_O | 220.1827 | 220[M+]  201[M-OH] |
| 27 | 31.25 | Dehydroxycostunolide | C_15_H_18_O_2_ | 232.1533 | 213[M-H_2_O]+  165[M-67] |
| 28 | 31.17 | isoalantolactone | C_15_H_20_O_2_ | 232.1458 | 233[M+]  255[M+Na] |
| 29 | 30.58 | Costunolide | C_15_H_20_O_2_ | 232.1463 | 233[M+]  205[M-CO] |
| 30 | 33.84 | Dehydrodiisoeugenol | [C_20_H_22_O_4_](https://pubchem.ncbi.nlm.nih.gov/#query=C20H22O4) | 326.1518 | 327[M+H]  203[M-123] |

**Table S3 Information table of the chemical composition of Shudage-4 in plasma**

| No. | tR/min | Identify | Formula |
| --- | --- | --- | --- |
| 1 | 3.21 | 5-hydroxy-1-phenyl (4-hydroxy-7 -(4-hydroxy-5-methoxy phenyl) -3-heptanone |  |
| 2 | 3.47 | Pinobaksin |  |
| 3 | 6.29 | Kaempferol |  |
| 4 | 7.09 | Curcumin |  |
| 5 | 7.81 | 3'-O-methylviolanone |  |
| 6 | 8.22 | Quercetin |  |
| 7 | 9.29 | 3,5-dihydroxy-1 -(4-hydroxy-3-methoxy phenyl) -7-phenylheptane |  |
| 8 | 10.09 | Pinocembrin |  |
| 9 | 10.83 | 5-hydroxy-7 - (4-hydroxy-3-methoxy phenyl) -1-phenyl-3-he*p*tanone |  |
| 10 | 11.62 | Kaempferide |  |
| 11 | 12.46 | Galangin |  |
| 12 | 13.64 | Galangin-3-methyl ether |  |
| 13 | 15.95 | 1,7-diphenyl-5-hydroxy-3-heptanone |  |

**Table S4** 1155 metabolites were identified in rat plasma

| **ID** | **m/z** | **Retention time (min)** | **Ion mode** | **Metabolites** |
| --- | --- | --- | --- | --- |
| 10.66_540.3317m/z | 540.332 | 10.6599 | neg | LysoPC(16:0) |
| 11.51_568.3630m/z | 568.363 | 11.5097 | neg | LysoPC(18:0) |
| 10.57_569.3478n | 570.355 | 10.5699 | pos | LysoPC(22:5(4Z,7Z,10Z,13Z,16Z)) |
| 10.39_588.3319m/z | 588.332 | 10.3887 | neg | LysoPC(20:4(5Z,8Z,11Z,14Z)) |
| 11.09_507.3686n | 508.376 | 11.0935 | pos | LysoPC(P-18:0) |
| 10.37_567.3319n | 568.339 | 10.3693 | pos | LysoPC(22:6(4Z,7Z,10Z,13Z,16Z,19Z)) |
| 7.87_451.2712m/z | 451.271 | 7.86677 | neg | 7-Ketodeoxycholic acid |
| 10.09_493.3164n | 494.324 | 10.0902 | pos | LysoPC(16:1(9Z)/0:0) |
| 11.05_554.3474m/z | 554.347 | 11.0534 | neg | LysoPC(17:0) |
| 12.72_747.5672m/z | 747.567 | 12.7159 | neg | SM(d18:0/16:1(9Z)) |
| 10.87_566.3475m/z | 566.348 | 10.8715 | neg | LysoPC(18:1(11Z)) |
| 11.60_826.5621m/z | 826.562 | 11.5995 | neg | PC(16:0/20:4(8Z,11Z,14Z,17Z)) |
| 3.57_253.1181m/z | 253.118 | 3.5682 | pos | Tyrosyl-Alanine |
| 9.93_382.2713m/z | 382.271 | 9.9323 | pos | Sphinganine 1-phosphate |
| 10.70_296.2580m/z | 296.258 | 10.6969 | pos | Alpha-Linolenic acid |
| 8.65_272.2582m/z | 272.258 | 8.65385 | pos | Palmitoleic acid |
| 11.68_594.3790m/z | 594.379 | 11.682 | neg | LysoPC(20:1(11Z)) |
| 7.91_390.2767n | 391.284 | 7.90562 | pos | Nutriacholic acid |
| 9.73_379.2482n | 380.255 | 9.7293 | pos | Sphingosine 1-phosphate |
| 4.63_284.0900n | 283.083 | 4.62687 | neg | p-Cresol glucuronide |
| 9.99_542.3238m/z | 542.324 | 9.99397 | pos | LysoPC(20:5(5Z,8Z,11Z,14Z,17Z)) |
| 6.40_499.2967n | 482.293 | 6.40375 | pos | Taurodeoxycholic acid |
| 7.51_465.3089n | 466.316 | 7.51057 | pos | Glycocholic acid |
| 7.24_585.2704m/z | 585.27 | 7.24037 | pos | Bilirubin |
| 10.44_263.2366m/z | 263.237 | 10.437 | pos | Linoleic acid |
| 13.24_780.5505m/z | 780.551 | 13.2396 | pos | PC(14:0/20:2(11Z,14Z)) |
| 13.28_506.3604m/z | 506.36 | 13.284 | pos | LysoPC(P-18:1(9Z)) |
| 6.16_433.1128m/z | 433.113 | 6.16138 | pos | Phloretin 2'-O-glucuronide |
| 7.27_448.3077m/z | 448.308 | 7.26802 | neg | Chenodeoxycholic acid glycine conjugate |
| 10.03_518.3239m/z | 518.324 | 10.0312 | pos | LysoPC(18:3(6Z,9Z,12Z)) |
| 3.99_293.1149m/z | 293.115 | 3.98965 | neg | gamma-Glutamylphenylalanine |
| 7.51_449.3141n | 432.311 | 7.51057 | pos | Deoxycholic acid glycine conjugate |
| 7.68_459.2036m/z | 459.204 | 7.68047 | neg | 2-Methoxy-estradiol-17b 3-glucuronide |
| 7.92_499.2964n | 500.304 | 7.91513 | pos | Tauroursodeoxycholic acid |
| 0.70_195.0506m/z | 195.051 | 0.70377 | neg | D-Ribose |
| 3.99_295.1287m/z | 295.129 | 3.98743 | pos | Tyrosyl-Hydroxyproline |
| 6.43_498.2882m/z | 498.288 | 6.43312 | pos | Taurocholic acid |
| 8.20_451.2712m/z | 451.271 | 8.19848 | neg | 3-Oxocholic acid |
| 10.48_255.2315m/z | 255.231 | 10.4804 | pos | 16-Hydroxy hexadecanoic acid |
| 13.11_740.5215m/z | 740.521 | 13.1081 | pos | PE(14:0/22:4(7Z,10Z,13Z,16Z)) |
| 0.72_247.0936m/z | 247.094 | 0.72283 | neg | Serylproline |
| 7.26_347.2215m/z | 347.221 | 7.25505 | pos | Cortexolone |
| 0.70_161.1050n | 162.112 | 0.6975 | pos | L-Carnitine |
| 8.82_408.2871n | 426.321 | 8.81702 | pos | Cholic acid |
| 10.66_480.3101m/z | 480.31 | 10.6599 | neg | LysoPC(15:0) |
| 5.67_282.1698m/z | 282.17 | 5.66665 | pos | (S)-Abscisic acid |
| 7.68_391.2840m/z | 391.284 | 7.68442 | pos | 3a,6b,7b-Trihydroxy-5b-cholanoic acid |
| 11.14_298.2737m/z | 298.274 | 11.1382 | pos | Bovinic acid |
| 13.24_809.5922n | 832.581 | 13.2396 | pos | PC(20:4(5Z,8Z,11Z,14Z)/18:0) |
| 4.47_336.0733m/z | 336.073 | 4.46767 | neg | 2,8-Dihydroxyquinoline-beta-D-glucuronide |
| 10.46_400.3416m/z | 400.342 | 10.4592 | pos | L-Palmitoylcarnitine |
| 10.76_570.3552m/z | 570.355 | 10.7628 | pos | LysoPC(22:5(7Z,10Z,13Z,16Z,19Z)) |
| 5.27_395.1176m/z | 395.118 | 5.26795 | neg | Estrone sulfate |
| 7.00_391.2840m/z | 391.284 | 7.00495 | pos | Alpha-Muricholic acid |
| 7.10_261.1484m/z | 261.148 | 7.09673 | pos | alpha-CEHC |
| 10.52_294.2192n | 277.216 | 10.5249 | pos | 17-Hydroxylinolenic acid |
| 11.69_549.3789n | 550.386 | 11.6942 | pos | PC(18:1(9Z)e/2:0) |
| 4.13_308.0781m/z | 308.078 | 4.1295 | neg | Acetaminophen glucuronide |
| 5.71_513.2760n | 514.283 | 5.70542 | pos | Sulfolithocholylglycine |
| 7.27_450.3211m/z | 450.321 | 7.26798 | pos | Glycoursodeoxycholic acid |
| 9.35_316.2844m/z | 316.284 | 9.3531 | pos | Ricinoleic acid |
| 10.39_457.3294m/z | 457.329 | 10.3919 | pos | 3a,7a,12a-Trihydroxy-5b-cholestan-26-al |
| 6.54_515.2914n | 498.288 | 6.54178 | pos | Tauro-b-muricholic acid |
| 9.97_294.2426m/z | 294.243 | 9.97128 | pos | Stearidonic acid |
| 4.40_273.0886m/z | 273.089 | 4.40107 | neg | Trigonelline |
| 0.70_146.0690n | 147.076 | 0.6975 | pos | L-Glutamine |
| 5.66_231.0762m/z | 231.076 | 5.65733 | pos | 5-Hydroxyindoleacetylglycine |
| 5.05_349.2372m/z | 349.237 | 5.04765 | pos | Cortolone |
| 6.48_188.0705m/z | 188.071 | 6.47857 | pos | 5-Methoxyindoleacetate |
| 10.76_431.3152m/z | 431.315 | 10.7628 | pos | 7alpha-Hydroxy-3-oxo-4-cholestenoate |
| 10.92_319.2285m/z | 319.229 | 10.9236 | neg | 12-HETE |
| 6.03_288.2167m/z | 288.217 | 6.02738 | pos | L-Octanoylcarnitine |
| 10.96_572.3706m/z | 572.371 | 10.9614 | pos | LysoPC(22:4(7Z,10Z,13Z,16Z)) |
| 4.49_463.0869m/z | 463.087 | 4.4892 | pos | Kaempferol 3-glucuronide |
| 6.77_246.2426m/z | 246.243 | 6.77143 | pos | Myristic acid |
| 0.80_204.1229m/z | 204.123 | 0.79692 | pos | L-Acetylcarnitine |
| 10.44_343.3315m/z | 343.331 | 10.437 | pos | N-Oleoylethanolamine |
| 6.63_347.2214m/z | 347.221 | 6.62555 | pos | Dihydrocortisol |
| 7.25_453.2868m/z | 453.287 | 7.25428 | neg | Hyocholic acid |
| 8.64_288.2895m/z | 288.289 | 8.64437 | pos | Heptadecanoic acid |
| 1.97_247.1287m/z | 247.129 | 1.97008 | pos | gamma-Glutamylvaline |
| 2.23_118.0653m/z | 118.065 | 2.22818 | pos | Benzeneacetonitrile |
| 0.72_118.0863m/z | 118.086 | 0.71572 | pos | 5-Aminopentanoic acid |
| 4.19_267.0879m/z | 267.088 | 4.19017 | neg | 5'-(3'-Methoxy-4'-hydroxyphenyl)-gamma-valerolactone |
| 5.12_447.0943m/z | 447.094 | 5.1213 | neg | Naringenin 4'-O-glucuronide |
| 6.81_500.3036m/z | 500.304 | 6.81222 | pos | Taurochenodesoxycholic acid |
| 9.46_455.3151m/z | 455.315 | 9.46137 | pos | 3beta,7alpha-Dihydroxy-5-cholestenoate |
| 0.64_146.1055n | 147.113 | 0.6363 | pos | L-Lysine |
| 10.53_311.2235m/z | 311.224 | 10.5329 | neg | 9,12,13-TriHOME |
| 11.41_332.0918m/z | 332.092 | 11.4124 | pos | S-(Formylmethyl)glutathione |
| 12.80_854.5932m/z | 854.593 | 12.7999 | neg | PC(20:3(8Z,11Z,14Z)/18:1(11Z)) |
| 4.97_246.1004n | 247.108 | 4.96817 | pos | N-acetyltryptophan |
| 11.99_885.5514m/z | 885.551 | 11.9879 | neg | PI(20:4(8Z,11Z,14Z,17Z)/18:0) |
| 1.05_167.0204m/z | 167.02 | 1.04538 | neg | Uric acid |
| 12.59_240.2319m/z | 240.232 | 12.5893 | pos | (-)-alpha-Bisabolol |
| 11.09_548.3706m/z | 548.371 | 11.0935 | pos | LysoPC(20:2(11Z,14Z)) |
| 10.94_279.2315m/z | 279.232 | 10.9393 | pos | 12,13-EpOME |
| 2.42_309.1098m/z | 309.11 | 2.4181 | neg | Phenylacetylglutamine |
| 9.03_381.3108m/z | 381.311 | 9.02627 | pos | 20-HETE ethanolamide |
| 4.08_273.0080m/z | 273.008 | 4.07695 | neg | Ferulic acid 4-sulfate |
| 4.67_246.1487m/z | 246.149 | 4.66848 | pos | Bisphenol A |
| 8.82_372.2660n | 373.273 | 8.81702 | pos | Cervonoyl ethanolamide |
| 1.01_245.0767m/z | 245.077 | 1.01192 | pos | Uridine |
| 13.24_828.5499m/z | 828.55 | 13.2396 | pos | PC(22:6(4Z,7Z,10Z,13Z,16Z,19Z)/18:3(6Z,9Z,12Z)) |
| 4.65_619.3445m/z | 619.344 | 4.65427 | pos | L-Urobilinogen |
| 9.82_299.2821n | 282.279 | 9.81888 | pos | Sphingosine |
| 1.14_303.0840m/z | 303.084 | 1.13647 | neg | Imidazoleacetic acid riboside |
| 13.33_520.5084m/z | 520.508 | 13.3281 | pos | Ceramide (d18:1/16:0) |
| 8.92_281.1016m/z | 281.102 | 8.92495 | pos | 2-Phenylethanol glucuronide |
| 10.17_285.2078m/z | 285.208 | 10.1719 | neg | Hexadecanedioic acid |
| 2.23_310.1282m/z | 310.128 | 2.22818 | pos | Arginyl-Proline |
| 7.52_459.2036m/z | 459.204 | 7.5192 | neg | 4-Hydroxyandrostenedione glucuronide |
| 9.61_211.1328m/z | 211.133 | 9.60675 | pos | Traumatic acid |
| 0.68_174.0878m/z | 174.088 | 0.68392 | neg | Citrulline |
| 3.81_259.1302m/z | 259.13 | 3.8062 | neg | gamma-Glutamylisoleucine |
| 9.56_302.3051m/z | 302.305 | 9.56012 | pos | Sphinganine |
| 12.48_752.5569m/z | 752.557 | 12.4815 | pos | PE(22:2(13Z,16Z)/16:1(9Z)) |
| 3.81_261.1441m/z | 261.144 | 3.8055 | pos | gamma-Glutamylleucine |
| 3.86_230.1022m/z | 230.102 | 3.85608 | pos | Vanillactic acid |
| 3.87_190.0497m/z | 190.05 | 3.87028 | pos | 4-(2-Aminophenyl)-2,4-dioxobutanoic acid |
| 0.73_189.0867m/z | 189.087 | 0.73408 | pos | N-Acetylglutamine |
| 1.06_290.1344m/z | 290.134 | 1.05543 | pos | Ophthalmic acid |
| 10.92_313.2392m/z | 313.239 | 10.9236 | neg | 9E-Heptadecenoic acid |
| 12.50_563.2646m/z | 563.265 | 12.5041 | pos | Protoporphyrin IX |
| 9.66_391.2839m/z | 391.284 | 9.66133 | pos | 3a,6a,7b-Trihydroxy-5b-cholanoic acid |
| 4.56_230.9969m/z | 230.997 | 4.55592 | neg | Hydroxymethoxyphenylcarboxylic acid-O-sulphate |
| 10.74_358.3674m/z | 358.367 | 10.741 | pos | Behenic acid |
| 0.68_257.1025n | 280.092 | 0.67747 | pos | Glycerophosphocholine |
| 0.78_227.0903n | 250.08 | 0.77695 | pos | Deoxycytidine |
| 10.23_330.3362m/z | 330.336 | 10.2349 | pos | Arachidic acid |
| 0.79_243.0624m/z | 243.062 | 0.78747 | neg | Pseudouridine |
| 11.98_238.2161m/z | 238.216 | 11.9795 | pos | 2-trans,6-trans-Farnesal |
| 12.65_269.2259m/z | 269.226 | 12.6528 | pos | 11-cis-Retinol |
| 9.10_344.2792m/z | 344.279 | 9.09793 | pos | Dodecanoylcarnitine |
| 11.41_266.2476m/z | 266.248 | 11.4124 | pos | (all-Z)-8,11,14-Heptadecatrienal |
| 2.41_311.1236m/z | 311.124 | 2.40895 | pos | gamma-Glutamyltyrosine |
| 0.79_233.0304m/z | 233.03 | 0.78747 | neg | (Z)-But-1-ene-1,2,4-tricarboxylate |
| 1.52_232.1289m/z | 232.129 | 1.51503 | pos | Asparaginyl-Valine |
| 0.70_165.0397m/z | 165.04 | 0.70377 | neg | L-Lyxonate |
| 1.52_373.1623m/z | 373.162 | 1.52015 | neg | 2-Keto-6-acetamidocaproate |
| 3.68_204.0897n | 227.079 | 3.67933 | pos | L-Tryptophan |
| 12.61_212.2005m/z | 212.201 | 12.6103 | pos | Theaspirane |
| 9.91_316.3207m/z | 316.321 | 9.90942 | pos | Nonadecanoic acid |
| 0.54_885.5508m/z | 885.551 | 0.53828 | neg | PI(20:3(8Z,11Z,14Z)/18:1(11Z)) |
| 0.76_229.1180m/z | 229.118 | 0.75592 | pos | Prolylhydroxyproline |
| 10.09_362.2319m/z | 362.232 | 10.0902 | pos | 11-Dehydrocorticosterone |
| 4.97_204.0664m/z | 204.066 | 4.9729 | neg | Indolelactic acid |
| 0.64_130.0862m/z | 130.086 | 0.6363 | pos | L-Pipecolic acid |
| 0.83_192.0264n | 191.019 | 0.83007 | neg | Isocitric acid |
| 0.72_273.0737m/z | 273.074 | 0.72283 | neg | Deoxyuridine |
| 1.05_611.1456m/z | 611.146 | 1.04538 | neg | Oxidized glutathione |
| 12.59_170.1537m/z | 170.154 | 12.5893 | pos | Dihydrocarvone |
| 9.61_318.3000m/z | 318.3 | 9.60675 | pos | Phytosphingosine |
| 0.80_160.1330m/z | 160.133 | 0.79692 | pos | DL-2-Aminooctanoic acid |
| 3.44_248.0951m/z | 248.095 | 3.44418 | pos | Thymol Sulfate |
| 5.27_497.0566m/z | 497.057 | 5.26795 | neg | Norepinephrine sulfate |
| 6.03_151.0393m/z | 151.039 | 6.03363 | neg | 2-Methoxybenzoic acid |
| 1.12_164.0472n | 182.081 | 1.11803 | pos | m-Coumaric acid |
| 11.23_205.0857m/z | 205.086 | 11.2255 | pos | Monoisobutyl phthalic acid |
| 12.61_135.1167m/z | 135.117 | 12.6103 | pos | (1S,4S)-Dihydrocarvone |
| 2.08_211.1076m/z | 211.108 | 2.08292 | pos | 4-Hydroxy-5-phenyltetrahydro-1,3-oxazin-2-one |
| 7.40_205.0858m/z | 205.086 | 7.40252 | pos | Monobutylphthalate |
| 0.68_174.1115n | 175.119 | 0.67747 | pos | L-Arginine |
| 1.05_288.1206m/z | 288.121 | 1.04538 | neg | Glutaminylproline |
| 12.04_271.2284m/z | 271.228 | 12.0446 | neg | 2-Pentadecanone |
| 3.68_146.0599m/z | 146.06 | 3.67933 | pos | 3-Methyldioxyindole |
| 4.29_338.0888m/z | 338.089 | 4.29192 | neg | 6-Hydroxy-5-methoxyindole glucuronide |
| 9.71_209.1898m/z | 209.19 | 9.71215 | pos | Myristoleic acid |
| 0.81_176.0314n | 175.024 | 0.80783 | neg | Ascorbic acid |
| 1.05_175.0242m/z | 175.024 | 1.04538 | neg | D-Glucuronic acid |
| 1.52_218.1386m/z | 218.139 | 1.52415 | pos | Propionylcarnitine |
| 11.45_338.3050m/z | 338.305 | 11.4548 | pos | 5alpha-Pregnane-3alpha,20alpha-diol |
| 8.77_583.2547m/z | 583.255 | 8.76553 | pos | Biliverdin |
| 1.12_135.0686n | 136.076 | 1.11803 | pos | 2-Phenylacetamide |
| 4.41_241.1068m/z | 241.107 | 4.41223 | pos | 3-(3,4,5-Trimethoxyphenyl)propanoic acid |
| 7.18_329.2341m/z | 329.234 | 7.18195 | neg | 9,10,13-TriHOME |
| 9.76_313.2392m/z | 313.239 | 9.76363 | neg | 12,13-DHOME |
| 9.84_261.2210m/z | 261.221 | 9.84135 | pos | Gamma-Linolenic acid |
| 0.72_131.0696n | 132.077 | 0.71572 | pos | Creatine |
| 13.96_124.0869m/z | 124.087 | 13.9571 | pos | L-Histidinol |
| 8.08_263.1294m/z | 263.129 | 8.08262 | neg | gamma-CEHC |
| 0.76_212.1027m/z | 212.103 | 0.75592 | pos | Zalcitabine |
| 0.64_133.0971m/z | 133.097 | 0.6363 | pos | Ornithine |
| 5.49_301.1181m/z | 301.118 | 5.49065 | pos | Pantetheine |
| 5.93_239.0926m/z | 239.093 | 5.93125 | neg | Butylparaben |
| 0.78_231.1701m/z | 231.17 | 0.77695 | pos | Valyl-Isoleucine |
| 10.19_333.2080m/z | 333.208 | 10.1888 | neg | 20-Hydroxy-leukotriene B4 |
| 10.28_424.3416m/z | 424.342 | 10.2801 | pos | trans-2-Tetradecenoylcarnitine |
| 4.97_292.1654m/z | 292.165 | 4.96817 | pos | Lysyl-Tyrosine |
| 1.10_180.0661m/z | 180.066 | 1.10368 | neg | o-Tyrosine |
| 3.82_371.0990m/z | 371.099 | 3.81532 | neg | Dihydroferulic acid 4-O-glucuronide |
| 9.82_249.1500m/z | 249.15 | 9.82117 | neg | 2,4-Di-tert-butylphenol |
| 0.78_112.0507m/z | 112.051 | 0.77695 | pos | Cytosine |
| 1.97_245.1145m/z | 245.114 | 1.96773 | neg | Glutamylvaline |
| 10.87_203.1429m/z | 203.143 | 10.8728 | pos | 2-Pentyl-3-phenyl-2-propenal |
| 11.35_324.2893m/z | 324.289 | 11.3483 | pos | 11,14,17-Eicosatrienoic acid |
| 12.39_830.5661m/z | 830.566 | 12.3933 | pos | PC(20:4(5Z,8Z,11Z,14Z)/20:4(8Z,11Z,14Z,17Z)) |
| 4.12_230.1034m/z | 230.103 | 4.11955 | neg | Suberylglycine |
| 0.70_258.1098m/z | 258.11 | 0.6975 | pos | N2-gamma-Glutamylglutamine |
| 3.94_275.0237m/z | 275.024 | 3.94092 | neg | Dihydroferulic acid 4-sulfate |
| 5.43_192.0654m/z | 192.065 | 5.43382 | pos | Hydroxyphenylacetylglycine |
| 9.11_378.2400m/z | 378.24 | 9.109 | pos | Oleoyl glycine |
| 1.12_268.1038m/z | 268.104 | 1.11803 | pos | Adenosine |
| 1.21_221.0918m/z | 221.092 | 1.2065 | pos | 5-Hydroxy-L-tryptophan |
| 1.64_261.0072m/z | 261.007 | 1.64148 | neg | Dihydrocaffeic acid 3-sulfate |
| 3.69_360.0862m/z | 360.086 | 3.6894 | neg | S-Lactoylglutathione |
| 4.64_231.1703m/z | 231.17 | 4.63642 | pos | Isoleucyl-Valine |
| 7.19_267.1243m/z | 267.124 | 7.19353 | neg | 1-Hydroxyibuprofen |
| 7.30_363.2186m/z | 363.219 | 7.29692 | neg | Leukotriene A4 |
| 0.73_143.0814m/z | 143.081 | 0.73408 | pos | 4-Acetamido-2-aminobutanoic acid |
| 0.95_348.0700m/z | 348.07 | 0.9467 | pos | Inosine 2',3'-cyclic phosphate |
| 1.06_126.0662m/z | 126.066 | 1.05543 | pos | 5-Methylcytosine |
| 14.07_319.2629m/z | 319.263 | 14.0675 | pos | Allopregnanolone |
| 3.37_232.1542m/z | 232.154 | 3.37427 | pos | Butyrylcarnitine |
| 3.39_246.9920m/z | 246.992 | 3.39165 | neg | Vanillic acid 4-sulfate |
| 4.03_269.0671m/z | 269.067 | 4.03028 | neg | Phenylglucuronide |
| 11.85_627.5339m/z | 627.534 | 11.8498 | pos | DG(18:0/20:4(5Z,8Z,11Z,14Z)/0:0) |
| 2.21_144.0655m/z | 144.065 | 2.2062 | pos | Acetylhomoserine |
| 5.97_221.0806m/z | 221.081 | 5.96542 | pos | 3,4,5-Trimethoxycinnamic acid |
| 7.01_357.0624m/z | 357.062 | 7.01425 | neg | Aflatoxin B1 |
| 0.80_157.0970m/z | 157.097 | 0.79692 | pos | N-Acetylornithine |
| 1.12_123.0441m/z | 123.044 | 1.11803 | pos | 2-Hydroxybenzaldehyde |
| 10.28_212.2007m/z | 212.201 | 10.2801 | pos | (3S,6E)-Nerolidol |
| 10.66_1103.6510m/z | 1103.65 | 10.6599 | neg | Lithocholate 3-O-glucuronide |
| 10.94_302.2242n | 303.232 | 10.9393 | pos | Eicosapentaenoic acid |
| 7.30_229.1446m/z | 229.145 | 7.29692 | neg | Undecylenic acid |
| 8.34_274.2737m/z | 274.274 | 8.3446 | pos | Palmitic acid |
| 10.61_282.2786m/z | 282.279 | 10.6138 | pos | Oleamide |
| 3.68_159.0915m/z | 159.092 | 3.67933 | pos | Serotonin |
| 0.73_116.0707m/z | 116.071 | 0.73408 | pos | L-Proline |
| 13.48_729.5892m/z | 729.589 | 13.4809 | pos | SM(d18:1/18:1(11Z)) |
| 4.86_206.0821m/z | 206.082 | 4.8642 | neg | N-Acetyl-L-phenylalanine |
| 0.92_243.0854n | 244.093 | 0.92497 | pos | Cytidine |
| 10.70_341.3158m/z | 341.316 | 10.6969 | pos | Linoleoyl ethanolamide |
| 4.94_171.0657m/z | 171.066 | 4.93947 | neg | 3-Hydroxysuberic acid |
| 6.27_238.1087m/z | 238.109 | 6.27148 | neg | (R)-N-Methylsalsolinol |
| 0.72_247.0921m/z | 247.092 | 0.71572 | pos | 5,6-Dihydrouridine |
| 12.65_552.4013m/z | 552.401 | 12.6528 | pos | LysoPC(20:0/0:0) |
| 4.17_219.1127m/z | 219.113 | 4.1738 | pos | N-Acetylserotonin |
| 5.43_176.0706m/z | 176.071 | 5.43382 | pos | Indoleacetic acid |
| 1.06_258.1084m/z | 258.108 | 1.05543 | pos | 5-Methylcytidine |
| 1.57_283.0689m/z | 283.069 | 1.56782 | neg | Xanthosine |
| 3.61_233.1031m/z | 233.103 | 3.613 | neg | Azelaic acid |
| 7.70_159.1020m/z | 159.102 | 7.6986 | neg | 7-Hydroxyoctanoic acid |
| 9.16_275.1638m/z | 275.164 | 9.16295 | pos | 2-Polyprenyl-6-methoxy-1,4-benzoquinone |
| 0.80_130.0862m/z | 130.086 | 0.79692 | pos | Pipecolic acid |
| 10.68_295.2282m/z | 295.228 | 10.6765 | neg | 9,10-Epoxyoctadecenoic acid |
| 11.98_192.1512n | 210.185 | 11.9795 | pos | alpha-Ionone |
| 3.59_144.0655m/z | 144.065 | 3.59143 | pos | Aminoadipic acid |
| 3.68_220.1178m/z | 220.118 | 3.67933 | pos | Pantothenic acid |
| 4.04_212.0021m/z | 212.002 | 4.03848 | neg | Indoxyl sulfate |
| 9.87_239.1655m/z | 239.166 | 9.87187 | neg | 4-(2,6,6-Trimethyl-1-cyclohexen-1-yl)-2-butanone |
| 0.70_180.0631n | 203.052 | 0.6975 | pos | D-Glucose |
| 1.61_203.0558m/z | 203.056 | 1.61242 | neg | Succinylacetone |
| 8.48_318.3000m/z | 318.3 | 8.47553 | pos | 2-Hydroxystearic acid |
| 0.76_170.0922m/z | 170.092 | 0.75592 | pos | 1-Methylhistidine |
| 0.80_143.1178m/z | 143.118 | 0.79692 | pos | N(6)-Methyllysine |
| 1.03_130.0862m/z | 130.086 | 1.03413 | pos | D-Pipecolic acid |
| 1.25_162.0527n | 145.049 | 1.25138 | pos | 2-Hydroxyadipic acid |
| 10.85_282.2788m/z | 282.279 | 10.8505 | pos | 3-Dehydrosphinganine |
| 3.88_114.0915m/z | 114.092 | 3.88253 | pos | Aminocaproic acid |
| 7.20_567.3187m/z | 567.319 | 7.20265 | neg | Deoxycholic acid 3-glucuronide |
| 2.11_191.0581n | 209.092 | 2.11177 | pos | 5-Phenyl-1,3-oxazinane-2,4-dione |
| 4.26_178.0504m/z | 178.05 | 4.25998 | neg | Hippuric acid |
| 4.65_172.0973m/z | 172.097 | 4.65342 | neg | N-Acetylleucine |
| 4.85_165.0551m/z | 165.055 | 4.84987 | neg | Homovanillin |
| 1.05_192.0264n | 191.019 | 1.04538 | neg | Diketogulonic acid |
| 1.42_159.0292m/z | 159.029 | 1.42132 | neg | Gluconolactone |
| 3.82_222.0771m/z | 222.077 | 3.81532 | neg | N-Acetyl-L-tyrosine |
| 5.31_218.2114m/z | 218.211 | 5.30767 | pos | Dodecanoic acid |
| 7.52_267.1742m/z | 267.174 | 7.52347 | pos | Boldione |
| 0.73_146.1173m/z | 146.117 | 0.73408 | pos | Pantolactone |
| 1.16_143.0338m/z | 143.034 | 1.1621 | pos | Oxoadipic acid |
| 8.25_185.1180m/z | 185.118 | 8.24532 | neg | 2-Nonenal |
| 8.92_105.0702m/z | 105.07 | 8.92495 | pos | 2-Phenylethanol |
| 0.73_102.0552m/z | 102.055 | 0.73408 | pos | L-Homoserine |
| 1.03_127.0390m/z | 127.039 | 1.03413 | pos | Maltol |
| 12.61_156.1381m/z | 156.138 | 12.6103 | pos | 2-Pentylfuran |
| 5.91_185.0816m/z | 185.082 | 5.91262 | neg | 2-Propyl-2,4-pentadienoic acid |
| 5.41_190.0498m/z | 190.05 | 5.4097 | pos | Kynurenic acid |
| 9.04_275.2002m/z | 275.2 | 9.04407 | pos | (2'E,4'Z,7'Z,8E)-Colnelenic acid |
| 9.96_191.1792m/z | 191.179 | 9.95558 | pos | 2-Methyl-2-phenyl-undecane |
| 0.72_132.0655m/z | 132.066 | 0.71572 | pos | L-Glutamic gamma-semialdehyde |
| 1.06_139.0501m/z | 139.05 | 1.05543 | pos | 4-Imidazolone-5-propionic acid |
| 10.78_314.3412m/z | 314.341 | 10.7846 | pos | Phytol |
| 2.05_282.1196m/z | 282.12 | 2.04503 | pos | 1-Methyladenosine |
| 4.33_165.0187m/z | 165.019 | 4.33353 | neg | Phthalic acid |
| 4.53_194.0812m/z | 194.081 | 4.53305 | pos | Phenylacetylglycine |
| 0.70_147.0531n | 130.05 | 0.6975 | pos | L-Glutamic acid |
| 0.74_157.0359m/z | 157.036 | 0.74313 | neg | Allantoic acid |
| 11.94_300.2896m/z | 300.29 | 11.937 | pos | Elaidic acid |
| 4.25_211.1440m/z | 211.144 | 4.2506 | pos | Isoleucylproline |
| 4.64_209.0453m/z | 209.045 | 4.64268 | neg | 3-(3,4-Dihydroxy-5-methoxy)-2-propenoic acid |
| 4.79_173.0814m/z | 173.081 | 4.79132 | neg | Cyclohexanecarboxylic acid |
| 5.20_273.0443m/z | 273.044 | 5.20445 | neg | 4-Hydroxy-5-(phenyl)-valeric acid-O-sulphate |
| 6.12_189.0789n | 190.086 | 6.117 | pos | Indole-3-propionic acid |
| 1.36_153.0658m/z | 153.066 | 1.36168 | pos | N1-Methyl-2-pyridone-5-carboxamide |
| 1.47_251.1059m/z | 251.106 | 1.46965 | pos | Methionyl-Threonine |
| 11.98_164.1199n | 182.154 | 11.9795 | pos | trans-Jasmone |
| 3.99_242.9971m/z | 242.997 | 3.98965 | neg | 3-hydroxy-3-(3-hydroxyphenyl)propanoic acid-O-sulphate |
| 4.19_159.0656m/z | 159.066 | 4.19017 | neg | Pimelic acid |
| 1.12_136.0526n | 119.049 | 1.11803 | pos | Phenylacetic acid |
| 10.87_104.1071m/z | 104.107 | 10.8728 | pos | (E)-2-Penten-1-ol |
| 13.76_267.2680m/z | 267.268 | 13.7613 | pos | Stearic acid |
| 2.21_164.0710m/z | 164.071 | 2.21423 | neg | L-Phenylalanine |
| 3.87_228.0877m/z | 228.088 | 3.86513 | neg | Epinephrine |
| 4.90_447.0942m/z | 447.094 | 4.8964 | neg | Naringenin 5-O-glucuronide |
| 7.13_214.1800m/z | 214.18 | 7.12898 | pos | alpha-Terpineol acetate |
| 0.68_188.1522n | 189.16 | 0.67747 | pos | N6,N6,N6-Trimethyl-L-lysine |
| 0.70_175.0955n | 198.085 | 0.6975 | pos | Argininic acid |
| 1.06_232.1056n | 233.113 | 1.05543 | pos | 4-(Glutamylamino) butanoate |
| 1.58_235.0488m/z | 235.049 | 1.58038 | pos | Questiomycin A |
| 14.70_433.3673m/z | 433.367 | 14.7026 | pos | 6-Deoxocastasterone |
| 5.55_224.0928m/z | 224.093 | 5.54523 | neg | 2(N)-Methyl-norsalsolinol |
| 9.16_338.2321m/z | 338.232 | 9.16295 | pos | 5'-Carboxy-alpha-chromanol |
| 10.26_448.3417m/z | 448.342 | 10.2575 | pos | LysoSM(d18:1) |
| 15.14_350.9892m/z | 350.989 | 15.1389 | neg | Sedoheptulose 1,7-bisphosphate |
| 4.01_174.0549m/z | 174.055 | 4.00937 | pos | 5-Hydroxyindoleacetic acid |
| 4.64_217.1080m/z | 217.108 | 4.64268 | neg | 9-Oxo-nonanoic acid |
| 5.24_187.0972m/z | 187.097 | 5.23628 | neg | 4-ene-Valproic acid |
| 6.59_307.2014m/z | 307.201 | 6.58797 | pos | Hyoscyamine |
| 7.20_283.1691m/z | 283.169 | 7.20353 | pos | 2-Methoxyestrone |
| 8.86_288.2894m/z | 288.289 | 8.8619 | pos | Methyl hexadecanoic acid |
| 0.76_174.1004n | 175.108 | 0.75592 | pos | Glycyl-Valine |
| 0.77_179.0555m/z | 179.056 | 0.76508 | neg | D-Galactose |
| 1.45_163.0389m/z | 163.039 | 1.44762 | pos | (E)-3-(2,3-Dihydroxyphenyl)-2-propenoic acid |
| 10.67_104.1071m/z | 104.107 | 10.6748 | pos | Pentanal |
| 12.72_128.1069m/z | 128.107 | 12.7167 | pos | 3-Methyl-2-cyclohexen-1-one |
| 3.66_204.0896n | 203.082 | 3.65817 | neg | D-Tryptophan |
| 4.46_447.2067m/z | 447.207 | 4.45855 | neg | 1-Methyl-6-phenyl-1H-imidazo[4,5-b]pyridin-2-amine |
| 4.56_187.0066m/z | 187.007 | 4.56472 | neg | p-Toluenesulfonic acid |
| 5.58_514.2854m/z | 514.285 | 5.58103 | neg | Taurohyocholate |
| 0.64_155.0693n | 156.077 | 0.6363 | pos | L-Histidine |
| 0.80_219.0973m/z | 219.097 | 0.79692 | pos | Alanyl-Glutamic acid |
| 3.68_298.0966m/z | 298.097 | 3.67933 | pos | 5'-Methylthioadenosine |
| 0.68_381.0251m/z | 381.025 | 0.67747 | pos | 6-Thioxanthine 5'-monophosphate |
| 11.94_199.1690m/z | 199.169 | 11.937 | pos | 3-Hydroxydodecanoic acid |
| 2.17_126.0550m/z | 126.055 | 2.17412 | pos | Quinone |
| 2.37_121.0649m/z | 121.065 | 2.37333 | pos | Phenylacetaldehyde |
| 2.73_216.9811m/z | 216.981 | 2.72932 | neg | 3-hydroxybenzoic acid-3-O-sulphate |
| 3.68_259.1302m/z | 259.13 | 3.67568 | neg | Glutamylisoleucine |
| 3.70_157.0499m/z | 157.05 | 3.70147 | neg | Isopropylmaleate |
| 3.91_142.0862m/z | 142.086 | 3.91417 | pos | Betonicine |
| 4.11_245.0127m/z | 245.013 | 4.10695 | neg | 4-Vinylphenol sulfate |
| 4.56_171.0657m/z | 171.066 | 4.55592 | neg | But-2-enoic acid |
| 5.07_203.0581n | 186.055 | 5.07315 | pos | Indolepyruvate |
| 1.01_209.0556m/z | 209.056 | 1.01192 | pos | 3-Nitrotyrosine |
| 1.01_217.1293m/z | 217.129 | 1.01192 | pos | N-a-Acetyl-L-arginine |
| 1.43_237.0867m/z | 237.087 | 1.4257 | pos | 2-Hydroxyfelbamate |
| 10.23_297.2665n | 280.263 | 10.2349 | pos | Palmitoleoyl Ethanolamide |
| 10.97_277.1450m/z | 277.145 | 10.9689 | neg | Monoethylhexyl phthalic acid |
| 11.54_104.1072m/z | 104.107 | 11.5425 | pos | 3-Methyl-3-buten-1-ol |
| 3.68_144.0807m/z | 144.081 | 3.67933 | pos | Tryptophanol |
| 6.93_345.2057m/z | 345.206 | 6.93277 | pos | Cortisol |
| 9.04_368.2793m/z | 368.279 | 9.04407 | pos | Tetrahydrodeoxycortisol |
| 9.54_391.2864m/z | 391.286 | 9.54133 | neg | Chenodeoxycholic acid |
| 10.41_240.2450n | 258.279 | 10.4145 | pos | Palmitaldehyde |
| 3.43_185.1284m/z | 185.128 | 3.42632 | pos | Alanyl-Isoleucine |
| 3.58_211.1440m/z | 211.144 | 3.58203 | pos | Leucylproline |
| 3.82_231.0874m/z | 231.087 | 3.8245 | neg | alpha-Ketoisovaleric acid |
| 6.90_163.0388m/z | 163.039 | 6.90232 | pos | (2E)-3-(2,3-dihydroxyphenyl)prop-2-enoic acid |
| 0.78_141.0657m/z | 141.066 | 0.77695 | pos | Imidazolepropionic acid |
| 1.47_184.0604m/z | 184.06 | 1.46965 | pos | 4-Pyridoxic acid |
| 2.21_107.0494m/z | 107.049 | 2.2062 | pos | Benzaldehyde |
| 4.33_182.0455m/z | 182.045 | 4.33353 | neg | 3-Pyridylacetic acid |
| 4.51_259.1190m/z | 259.119 | 4.51327 | neg | Ketoleucine |
| 5.09_188.1645m/z | 188.164 | 5.086 | pos | 8-Methylnonenoate |
| 5.72_403.1406m/z | 403.141 | 5.71905 | neg | (-)-Matairesinol |
| 8.82_504.2402m/z | 504.24 | 8.81702 | pos | N-Acetyl-leukotriene E4 |
| 9.74_283.1921m/z | 283.192 | 9.7394 | neg | (3Z)-2-Propylpent-3-enoic acid |
| 1.40_132.1019m/z | 132.102 | 1.40362 | pos | L-Norleucine |
| 2.82_279.0749m/z | 279.075 | 2.82342 | pos | O-Phosphotyrosine |
| 3.68_187.0632n | 188.07 | 3.67933 | pos | Indoleacrylic acid |
| 8.92_120.0577n | 121.065 | 8.92495 | pos | 2-Methylbenzaldehyde |
| 1.28_276.0180m/z | 276.018 | 1.27892 | neg | Paracetamol sulfate |
| 2.23_148.0524n | 166.086 | 2.22818 | pos | (E)-3-(4-Hydroxyphenyl)-2-propenal |
| 3.43_126.0914m/z | 126.091 | 3.42632 | pos | Proline betaine |
| 0.97_231.1701m/z | 231.17 | 0.96852 | pos | Leucyl-Valine |
| 1.76_121.0649m/z | 121.065 | 1.7619 | pos | 2-Methoxy-4-methylphenol |
| 10.74_372.2740m/z | 372.274 | 10.741 | pos | Prostaglandin F2a |
| 12.48_731.6049m/z | 731.605 | 12.4815 | pos | SM(d18:0/18:1(11Z)) |
| 13.04_328.3205m/z | 328.321 | 13.043 | pos | 11Z-Eicosenoic acid |
| 2.52_158.0811m/z | 158.081 | 2.52225 | pos | 3-Methylcrotonylglycine |
| 3.37_159.0656m/z | 159.066 | 3.36972 | neg | 3-Methyladipic acid |
| 5.98_283.1689m/z | 283.169 | 5.97923 | pos | Adrenosterone |
| 2.01_171.0657m/z | 171.066 | 2.00918 | neg | Gamma-Butyrolactone |
| 2.37_145.1334m/z | 145.133 | 2.37333 | pos | N-Acetylcadaverine |
| 3.66_159.0683n | 160.076 | 3.65818 | pos | Indoleacetaldehyde |
| 3.70_172.9908m/z | 172.991 | 3.70147 | neg | Phenol sulphate |
| 4.51_181.0859m/z | 181.086 | 4.51147 | pos | 3-Methoxybenzenepropanoic acid |
| 5.33_447.0919m/z | 447.092 | 5.32815 | pos | Isoquercitrin |
| 5.46_217.1080m/z | 217.108 | 5.45552 | neg | 2-Hydroxydecanedioic acid |
| 0.68_154.0614m/z | 154.061 | 0.68392 | neg | 2-Oxoarginine |
| 1.05_316.1154m/z | 316.115 | 1.04538 | neg | Aspartylglycosamine |
| 1.45_182.0454m/z | 182.045 | 1.45255 | neg | 2-Aminobenzoic acid |
| 10.39_356.3519m/z | 356.352 | 10.3919 | pos | Erucic acid |
| 11.98_118.0652m/z | 118.065 | 11.9795 | pos | Indole |
| 3.68_261.0079m/z | 261.008 | 3.67568 | neg | Homovanillic acid sulfate |
| 4.56_259.0285m/z | 259.029 | 4.56472 | neg | Fluorouracil |
| 6.75_144.1382m/z | 144.138 | 6.7469 | pos | (E)-2-octenal |
| 8.26_315.1819m/z | 315.182 | 8.25772 | neg | 2-n-Propyl-4-oxopentanoic acid |
| 9.45_391.2864m/z | 391.286 | 9.45495 | neg | Deoxycholic acid |
| 9.96_111.1170m/z | 111.117 | 9.95558 | pos | (R)-1-Octen-3-ol |
| 0.68_304.0256n | 327.015 | 0.67747 | pos | 5-(3',4',5'-Trihydroxyphenyl)-gamma-valerolactone-3'-O-sulphate |
| 10.21_149.0960m/z | 149.096 | 10.2125 | pos | Perillic acid |
| 10.52_101.0236m/z | 101.024 | 10.5249 | pos | Methylmalonic acid |
| 12.15_326.3047m/z | 326.305 | 12.1535 | pos | Eicosadienoic acid |
| 14.72_299.0613m/z | 299.061 | 14.7249 | pos | Flutamide |
| 4.10_179.1178m/z | 179.118 | 4.09642 | pos | Glycinexylidide |
| 4.55_186.1131m/z | 186.113 | 4.5452 | neg | (±)-Pelletierine |
| 7.86_405.2655m/z | 405.265 | 7.85608 | neg | 3a,6b,7a,12a-Tetrahydroxy-5b-cholanoic acid |
| 9.88_167.0701m/z | 167.07 | 9.87727 | pos | 3-(3-Hydroxyphenyl)propanoic acid |
| 1.06_130.0499m/z | 130.05 | 1.05543 | pos | Pyroglutamic acid |
| 1.39_161.0448m/z | 161.045 | 1.39452 | neg | 3-Hydroxymethylglutaric acid |
| 10.24_392.2936n | 391.286 | 10.2394 | neg | Isohyodeoxycholic acid |
| 3.82_158.0816m/z | 158.082 | 3.8245 | neg | 5-Acetamidovalerate |
| 3.92_158.0816m/z | 158.082 | 3.919 | neg | 2-Methylbutyrylglycine |
| 5.15_163.0389m/z | 163.039 | 5.1451 | pos | Umbelliferone |
| 5.89_201.1129m/z | 201.113 | 5.8856 | neg | Sebacic acid |
| 9.07_338.9893m/z | 338.989 | 9.0664 | neg | D-Glyceraldehyde 3-phosphate |
| 2.65_202.1436m/z | 202.144 | 2.64622 | pos | Capryloylglycine |
| 4.61_158.0448m/z | 158.045 | 4.61332 | pos | 2-Aminomuconic acid |
| 9.07_361.1641m/z | 361.164 | 9.0664 | neg | Thyrotropin releasing hormone |
| 0.72_160.0602m/z | 160.06 | 0.71572 | pos | Sumiki's acid |
| 13.91_383.3666m/z | 383.367 | 13.9134 | pos | Campesterol |
| 4.53_192.0663m/z | 192.066 | 4.52753 | neg | 3-Carbamoyl-2-phenylpropionaldehyde |
| 4.77_287.0549m/z | 287.055 | 4.76692 | pos | Luteolin |
| 10.52_333.2055m/z | 333.206 | 10.5225 | neg | Prostaglandin E2 |
| 10.74_455.3177m/z | 455.318 | 10.7401 | neg | Gamma-Tocotrienol |
| 12.02_223.2054m/z | 223.205 | 12.0228 | pos | Farnesol |
| 12.50_796.5232m/z | 796.523 | 12.5041 | pos | PC(14:1(9Z)/20:1(11Z)) |
| 3.63_198.0527n | 181.049 | 3.62693 | pos | Vanillylmandelic acid |
| 5.29_144.0655m/z | 144.065 | 5.29307 | pos | Methyl 2-furoate |
| 5.87_159.1020m/z | 159.102 | 5.86877 | neg | 3-Hydroxyoctanoic acid |
| 1.06_192.0268n | 215.016 | 1.05543 | pos | Citric acid |
| 1.12_171.1127m/z | 171.113 | 1.11803 | pos | N6-Acetyl-L-lysine |
| 11.21_339.2548m/z | 339.255 | 11.2148 | neg | Linalyl oxide |
| 11.69_794.6033m/z | 794.603 | 11.6942 | pos | PC(20:1(11Z)/P-16:0) |
| 11.98_206.1667n | 224.201 | 11.9795 | pos | 4-(1,1,3,3-Tetramethylbutyl)-phenol |
| 12.35_327.2336m/z | 327.234 | 12.3505 | neg | Docosahexaenoic acid |
| 13.20_152.9947m/z | 152.995 | 13.1958 | pos | Dihydroxyacetone phosphate |
| 13.24_768.5870m/z | 768.587 | 13.2396 | pos | PC(18:1(11Z)/18:1(11Z)) |
| 2.13_127.0502m/z | 127.05 | 2.12793 | pos | Thymine |
| 5.79_186.0557m/z | 186.056 | 5.79288 | neg | Cinnamoylglycine |
| 8.79_172.1694m/z | 172.169 | 8.79433 | pos | beta-Geraniol |
| 9.75_135.0802m/z | 135.08 | 9.75132 | pos | 1-Phenyl-1-propanone |
| 0.70_104.1072m/z | 104.107 | 0.6975 | pos | 1-Penten-3-ol |
| 0.70_137.0708m/z | 137.071 | 0.6975 | pos | N-Methylnicotinamide |
| 1.15_203.0558m/z | 203.056 | 1.14835 | neg | Acetoacetic acid |
| 1.23_176.9718m/z | 176.972 | 1.22872 | pos | Fosfomycin |
| 1.36_153.0426n | 154.05 | 1.36168 | pos | 3-Hydroxyanthranilic acid |
| 4.56_151.0393m/z | 151.039 | 4.55592 | neg | 3,4-Dihydroxyphenylglycol |
| 8.95_172.1694m/z | 172.169 | 8.94742 | pos | 3,7-Dimethyl-1,6-octadien-3-ol |
| 0.79_249.0619m/z | 249.062 | 0.78747 | neg | Inosine |
| 1.06_123.0554m/z | 123.055 | 1.05543 | pos | Pi-Methylimidazoleacetic acid |
| 1.07_307.0352m/z | 307.035 | 1.06647 | neg | dUMP |
| 10.23_364.2479m/z | 364.248 | 10.2349 | pos | 21-Hydroxy-5b-pregnane-3,11,20-trione |
| 10.26_147.0439m/z | 147.044 | 10.2575 | pos | 4-Hydroxycinnamic acid |
| 13.24_794.6031m/z | 794.603 | 13.2396 | pos | PC(20:0/18:3(6Z,9Z,12Z)) |
| 2.12_242.0904n | 241.083 | 2.12063 | neg | Thymidine |
| 4.03_133.0528n | 134.06 | 4.03185 | pos | Indoxyl |
| 4.70_163.0394m/z | 163.039 | 4.6992 | neg | Phenylpyruvic acid |
| 4.85_193.0495m/z | 193.05 | 4.84592 | pos | Vanilpyruvic acid |
| 5.53_199.0972m/z | 199.097 | 5.5314 | neg | 3-Hydroxysebacic acid |
| 9.63_260.1278m/z | 260.128 | 9.62775 | pos | Equol |
| 0.78_305.0842m/z | 305.084 | 0.77695 | pos | Xylobiose |
| 10.89_289.1560m/z | 289.156 | 10.895 | pos | Normorphine |
| 10.96_286.3100m/z | 286.31 | 10.9614 | pos | Stearaldehyde |
| 11.23_121.0285m/z | 121.028 | 11.2255 | pos | 4-Hydroxybenzoic acid |
| 3.77_156.0659m/z | 156.066 | 3.77038 | neg | Tiglylglycine |
| 3.88_108.0811m/z | 108.081 | 3.88253 | pos | Benzylamine |
| 4.90_172.0973m/z | 172.097 | 4.8964 | neg | Hexanoylglycine |
| 9.76_215.1652m/z | 215.165 | 9.75512 | neg | Undecanal |
| 0.70_117.0788n | 140.068 | 0.6975 | pos | L-Valine |
| 1.10_188.0561m/z | 188.056 | 1.10368 | neg | N-Acetylglutamic acid |
| 10.52_157.0858m/z | 157.086 | 10.5249 | pos | 2-Propylglutaric acid |
| 13.02_731.6048m/z | 731.605 | 13.0211 | pos | SM(d18:0/18:1(9Z)) |
| 4.61_249.0883m/z | 249.088 | 4.60758 | neg | (±)-Tryptophan |
| 5.05_259.0468m/z | 259.047 | 5.04967 | neg | Citraconic acid |
| 5.26_343.2590m/z | 343.259 | 5.2613 | pos | 10-Nitrolinoleic acid |
| 6.61_261.1484m/z | 261.148 | 6.60863 | pos | Diisobutyl phthalate |
| 7.72_165.0909m/z | 165.091 | 7.72012 | pos | 2-Methylbenzyl alcohol acetate |
| 9.61_183.0804m/z | 183.08 | 9.60675 | pos | Benzophenone |
| 0.79_300.0942m/z | 300.094 | 0.78747 | neg | 7,8-Dihydroneopterin |
| 0.94_217.0119m/z | 217.012 | 0.93742 | neg | Glycerol 3-phosphate |
| 2.89_203.0558m/z | 203.056 | 2.89157 | neg | 2-Methyl-3-oxopropanoic acid |
| 4.32_174.0885n | 173.081 | 4.31522 | neg | Suberic acid |
| 6.05_123.0805m/z | 123.081 | 6.05277 | pos | 4-Ethylphenol |
| 8.08_193.0868m/z | 193.087 | 8.08262 | neg | Cuminaldehyde |
| 8.42_230.2477m/z | 230.248 | 8.42002 | pos | 2-Tetradecanone |
| 8.78_269.0461m/z | 269.046 | 8.78343 | neg | Aromadendrin |
| 0.62_171.0054m/z | 171.005 | 0.61677 | pos | (S)-2-Acetolactate |
| 10.07_442.3522m/z | 442.352 | 10.0679 | pos | 2,3-Diaminosalicylic acid |
| 11.18_127.0866m/z | 127.087 | 11.1816 | pos | 4-Aminophenol |
| 3.29_143.0702m/z | 143.07 | 3.29408 | pos | Choline |
| 3.29_168.0655m/z | 168.065 | 3.29408 | pos | Phenylglyoxylic acid |
| 3.78_197.1283m/z | 197.128 | 3.7846 | pos | (R)-Salsolinol |
| 3.97_133.0859m/z | 133.086 | 3.9651 | pos | 6-Hydroxyhexanoic acid |
| 4.20_144.0655m/z | 144.065 | 4.20417 | pos | 1,3,5-Trihydroxybenzene |
| 4.32_181.0971m/z | 181.097 | 4.32107 | pos | 3-Hydroxykynurenamine |
| 5.88_269.0457m/z | 269.046 | 5.87788 | neg | Genistein |
| 9.24_237.1498m/z | 237.15 | 9.23715 | neg | beta-Ionone |
| 9.73_128.1203n | 129.127 | 9.7293 | pos | 2-Octanone |
| 9.88_136.0756m/z | 136.076 | 9.87727 | pos | Dopamine |
| 1.05_231.0988m/z | 231.099 | 1.04538 | neg | Alanyl-Proline |
| 1.14_262.0379m/z | 262.038 | 1.13987 | pos | 2-Methoxyacetaminophen sulfate |
| 1.87_204.0267m/z | 204.027 | 1.87262 | pos | 2-Phospho-D-glyceric acid |
| 10.52_231.1588m/z | 231.159 | 10.5249 | pos | Dodecanedioic acid |
| 13.20_203.0215m/z | 203.021 | 13.1958 | pos | Imidazole acetol-phosphate |
| 2.13_168.0654m/z | 168.065 | 2.12793 | pos | Isopyridoxal |
| 2.56_204.0267m/z | 204.027 | 2.56047 | pos | 2-Phosphoglyceric acid |
| 3.27_166.0499m/z | 166.05 | 3.27323 | pos | Formylanthranilic acid |
| 3.63_168.0655m/z | 168.066 | 3.62693 | pos | Pyridoxal |
| 4.65_187.0971m/z | 187.097 | 4.65342 | neg | cis-3-Hexenyl acetate |
| 4.87_208.0968m/z | 208.097 | 4.86618 | pos | Phenylpropionylglycine |
| 5.18_189.1128m/z | 189.113 | 5.1824 | neg | Caprylic acid |
| 5.40_228.0280m/z | 228.028 | 5.3976 | neg | 5-Phosphoribosylamine |
| 6.91_227.1289m/z | 227.129 | 6.91498 | neg | Delta-Hexanolactone |
| 0.56_172.9961m/z | 172.996 | 0.556 | pos | (S)-Ureidoglycolic acid |
| 1.12_121.0649m/z | 121.065 | 1.11803 | pos | Tyrosol |
| 1.34_228.0715m/z | 228.071 | 1.33868 | pos | Galactaric acid |
| 10.09_179.0558m/z | 179.056 | 10.0866 | neg | Hydroxypropionic acid |
| 11.03_356.2790m/z | 356.279 | 11.0275 | pos | 8,9-DiHETrE |
| 11.23_190.0497m/z | 190.05 | 11.2255 | pos | 1-Nitro-5,6-dihydroxy-dihydronaphthalene |
| 13.72_101.0713m/z | 101.071 | 13.7176 | pos | L-2,4-diaminobutyric acid |
| 2.61_225.0880m/z | 225.088 | 2.61243 | neg | 5-Hydroxykynurenamine |
| 3.48_204.0267m/z | 204.027 | 3.47638 | pos | 3-Phosphoglyceric acid |
| 3.64_227.1753m/z | 227.175 | 3.64135 | pos | Isoleucyl-Isoleucine |
| 4.01_228.0877m/z | 228.088 | 4.00567 | neg | Normetanephrine |
| 4.99_298.1284m/z | 298.128 | 4.99225 | pos | Phenethylamine glucuronide |
| 5.18_204.0664m/z | 204.066 | 5.1824 | neg | N-Hydroxy-1-aminonaphthalene |
| 5.66_179.0558m/z | 179.056 | 5.65913 | neg | L-Lactic acid |
| 6.12_125.0962m/z | 125.096 | 6.117 | pos | 6-Methyl-3,5-heptadien-2-one |
| 8.88_244.2632m/z | 244.263 | 8.88085 | pos | Pentadecanal |
| 0.51_180.1130m/z | 180.113 | 0.5112 | pos | L-Histidine trimethylbetaine |
| 0.58_127.0390m/z | 127.039 | 0.578 | pos | benzene-1,2,4-triol |
| 0.68_152.0681m/z | 152.068 | 0.67747 | pos | N4-Acetylaminobutanal |
| 0.80_133.0608m/z | 133.061 | 0.79692 | pos | Ureidopropionic acid |
| 0.82_206.9665m/z | 206.966 | 0.81743 | pos | Phosphohydroxypyruvic acid |
| 0.92_194.0421m/z | 194.042 | 0.92497 | pos | Tetrahydrodipicolinate |
| 0.99_160.1331m/z | 160.133 | 0.9902 | pos | Methyl cyclohexanecarboxylate |
| 1.01_146.0923m/z | 146.092 | 1.01192 | pos | 4-Guanidinobutanoic acid |
| 1.03_203.1390m/z | 203.139 | 1.03413 | pos | Alanyl-Leucine |
| 1.39_231.0194n | 276.018 | 1.39452 | neg | Benzeneacetamide-4-O-sulphate |
| 1.98_207.0764m/z | 207.076 | 1.98122 | pos | 1-Nitronaphthalene-5,6-oxide |
| 11.00_179.0559m/z | 179.056 | 11.0001 | neg | D-Lactic acid |
| 11.05_104.1071m/z | 104.107 | 11.0497 | pos | Prenol |
| 11.16_179.0559m/z | 179.056 | 11.1643 | neg | Glyceraldehyde |
| 12.48_180.9895m/z | 180.99 | 12.4815 | pos | Dihydroxyacetone Phosphate Acyl Ester |
| 13.13_285.2212m/z | 285.221 | 13.1303 | pos | Retinal |
| 13.31_730.5371m/z | 730.537 | 13.3062 | pos | PC(16:1(9Z)/16:1(9Z)) |
| 2.11_207.0773m/z | 207.077 | 2.10807 | neg | L-Kynurenine |
| 4.60_201.1129m/z | 201.113 | 4.59682 | neg | Dihydro-5-pentyl-2(3H)-furanone |
| 4.67_259.1516m/z | 259.152 | 4.66848 | pos | Tetrahydrobiopterin |
| 4.73_192.0663m/z | 192.066 | 4.73262 | neg | 2-Methylhippuric acid |
| 4.89_291.1339m/z | 291.134 | 4.88873 | pos | L-Thyronine |
| 5.17_151.1117m/z | 151.112 | 5.17368 | pos | Cuminyl alcohol |
| 6.64_361.1124m/z | 361.112 | 6.64093 | neg | 8-Hydroxy-7-methylguanine |
| 7.96_187.1336m/z | 187.134 | 7.96128 | neg | 2-Nonanone |
| 8.50_225.1483m/z | 225.148 | 8.49865 | pos | Methyl jasmonate |
| 9.28_179.0558m/z | 179.056 | 9.28325 | neg | Dihydroxyacetone |
| 9.66_149.0960m/z | 149.096 | 9.66133 | pos | Anethole |
| 9.75_396.3103m/z | 396.31 | 9.75132 | pos | 2-Arachidonylglycerol |
| 0.62_275.0565m/z | 275.056 | 0.6222 | neg | Salicylic acid |
| 0.74_199.0375m/z | 199.037 | 0.74313 | neg | Diethylphosphate |
| 1.05_256.0941m/z | 256.094 | 1.04538 | neg | N-gamma-Glutamylglutamine |
| 1.12_204.0631m/z | 204.063 | 1.11803 | pos | L-Tyrosine |
| 10.47_255.2331m/z | 255.233 | 10.4658 | neg | 1-Pentadecene |
| 12.20_199.1803m/z | 199.18 | 12.1982 | pos | N-Ethyl trans-2-cis-6-nonadienamide |
| 13.24_180.9896m/z | 180.99 | 13.2396 | pos | cis,cis-Muconic acid |
| 14.29_170.0962m/z | 170.096 | 14.2876 | pos | 4-Aminobiphenyl |
| 14.51_381.3503m/z | 381.35 | 14.5085 | pos | Episterol |
| 15.50_152.9959m/z | 152.996 | 15.4982 | neg | Beta-Glycerophosphoric acid |
| 3.66_204.0300m/z | 204.03 | 3.65817 | neg | Xanthurenic acid |
| 3.81_209.1284m/z | 209.128 | 3.8055 | pos | 5-Methoxytryptophol |
| 4.35_267.1336m/z | 267.134 | 4.35432 | pos | Phenylalanyl-Threonine |
| 4.59_244.0231m/z | 244.023 | 4.58522 | neg | O-Phosphohomoserine |
| 4.73_215.0200m/z | 215.02 | 4.73262 | neg | Gallic acid |
| 5.26_240.1593m/z | 240.159 | 5.2613 | pos | 2-Hydroxyibuprofen |
| 5.57_196.0977m/z | 196.098 | 5.5719 | neg | N-Methyltyramine |
| 8.14_211.1338m/z | 211.134 | 8.13563 | neg | Traumatin |
| 8.90_146.0578n | 147.065 | 8.90303 | pos | Adipic acid |
| 0.51_164.1181m/z | 164.118 | 0.5112 | pos | Dimethylbenzimidazole |
| 0.77_237.0617m/z | 237.062 | 0.76508 | neg | Quinic acid |
| 1.06_166.0723m/z | 166.072 | 1.05543 | pos | 7-Methylguanine |
| 1.60_168.0655m/z | 168.065 | 1.6013 | pos | 3-Methoxyanthranilate |
| 11.33_311.1621m/z | 311.162 | 11.3264 | pos | Estriol |
| 11.36_295.2284m/z | 295.228 | 11.3584 | neg | Alpha-dimorphecolic acid |
| 11.96_109.1013m/z | 109.101 | 11.9582 | pos | Sulcatone |
| 13.24_796.5236m/z | 796.524 | 13.2396 | pos | PE(20:3(8Z,11Z,14Z)/22:6(4Z,7Z,10Z,13Z,16Z,19Z)) |
| 13.42_125.0710m/z | 125.071 | 13.4158 | pos | Methylimidazole acetaldehyde |
| 14.55_391.2838m/z | 391.284 | 14.5534 | pos | Ursocholic acid |
| 2.08_235.0823m/z | 235.082 | 2.07747 | neg | Galactosylglycerol |
| 2.84_115.0391m/z | 115.039 | 2.84462 | pos | Glutaric acid |
| 3.78_267.0514m/z | 267.051 | 3.7827 | neg | Diphenol glucuronide |
| 4.37_158.0816m/z | 158.082 | 4.36615 | neg | Isovalerylglycine |
| 4.61_190.0498m/z | 190.05 | 4.61332 | pos | 1-Nitronaphthalene-7,8-oxide |
| 5.21_259.0467m/z | 259.047 | 5.21337 | neg | Mesaconic acid |
| 5.40_263.0598m/z | 263.06 | 5.3976 | neg | 5,6-Dihydro-5-fluorouracil |
| 6.30_333.2058m/z | 333.206 | 6.30307 | pos | 11b-Hydroxyprogesterone |
| 8.14_426.9689m/z | 426.969 | 8.13563 | neg | 2-Oxo-3-hydroxy-4-phosphobutanoic acid |
| 0.68_188.1756m/z | 188.176 | 0.67747 | pos | N1-Acetylspermidine |
| 0.70_151.0476m/z | 151.048 | 0.6975 | pos | Dihydrothymine |
| 0.88_223.1438m/z | 223.144 | 0.88253 | pos | 3-Hydroxymonoethylglycinexylidide |
| 1.06_113.0348m/z | 113.035 | 1.05543 | pos | Uracil |
| 10.18_144.0654m/z | 144.065 | 10.1842 | pos | 1,2,3-Trihydroxybenzene |
| 10.60_239.0675m/z | 239.068 | 10.5958 | neg | 4-Aminohippuric acid |
| 10.66_255.2326m/z | 255.233 | 10.6599 | neg | Ethyl pentyl ketone |
| 10.92_179.0558m/z | 179.056 | 10.9236 | neg | (R)-2,3-Dihydroxy-isovalerate |
| 2.41_171.0657m/z | 171.066 | 2.4088 | neg | Ethenyl acetate |
| 2.75_156.0420m/z | 156.042 | 2.74795 | pos | N-Methylethanolaminium phosphate |
| 3.78_207.1127m/z | 207.113 | 3.7846 | pos | Indole-3-methyl acetate |
| 4.25_265.1181m/z | 265.118 | 4.2506 | pos | Acetyl-N-formyl-5-methoxykynurenamine |
| 4.28_267.0425m/z | 267.042 | 4.28423 | pos | Resveratrol |
| 4.55_152.1069m/z | 152.107 | 4.55177 | pos | Phenylpropanolamine |
| 4.61_245.0309m/z | 245.031 | 4.60758 | neg | 4-Fumarylacetoacetic acid |
| 5.11_107.0858m/z | 107.086 | 5.10983 | pos | o-Xylene |
| 5.22_201.0224m/z | 201.022 | 5.2223 | neg | 4-ethylphenylsulfate |
| 5.33_244.0232m/z | 244.023 | 5.33378 | neg | O-Phosphothreonine |
| 5.37_242.0576m/z | 242.058 | 5.36663 | pos | 3-Indolebutyric acid |
| 6.83_223.0975m/z | 223.098 | 6.82907 | neg | (2E,4E)-2,4-Hexadienoic acid |
| 7.02_777.6931m/z | 777.693 | 7.02388 | pos | Thyroxine |
| 7.25_391.2135m/z | 391.214 | 7.25428 | neg | Corticosterone |
| 7.47_187.1336m/z | 187.134 | 7.46528 | neg | 2-Methyl-4-heptanone |
| 7.64_187.1336m/z | 187.134 | 7.64457 | neg | 2,6-Dimethyl-4-heptanone |
| 9.68_194.0821m/z | 194.082 | 9.67573 | neg | 2-Phenyl-1,3-propanediol monocarbamate |
| 9.77_144.0653m/z | 144.065 | 9.77395 | pos | N-methyl-L-glutamic Acid |
| 9.77_234.1851m/z | 234.185 | 9.77395 | pos | 2-Hexyl-3-phenyl-2-propenal |
| 0.52_102.1280m/z | 102.128 | 0.51668 | pos | Cyclohexane |
| 0.62_148.9646m/z | 148.965 | 0.6222 | neg | Phosphoenolpyruvic acid |
| 0.68_266.0883m/z | 266.088 | 0.68392 | neg | N-Acetylgalactosamine |
| 0.70_203.1501m/z | 203.15 | 0.6975 | pos | Asymmetric dimethylarginine |
| 1.35_264.9529m/z | 264.953 | 1.35397 | neg | 2,3-Diphosphoglyceric acid |
| 1.55_211.0845n | 212.092 | 1.55353 | pos | 3-Methoxytyrosine |
| 11.77_179.0559m/z | 179.056 | 11.767 | neg | myo-Inositol |
| 12.59_163.1478m/z | 163.148 | 12.5893 | pos | 1,3-Diisopropylbenzene |
| 2.24_253.0832m/z | 253.083 | 2.24282 | neg | Formyl-5-hydroxykynurenamine |
| 2.74_184.0967m/z | 184.097 | 2.73528 | pos | 3-(2-Hydroxyphenyl)propanoic acid |
| 3.10_186.0760m/z | 186.076 | 3.10233 | pos | 3,4-Dihydroxymandelaldehyde |
| 3.91_113.1076m/z | 113.108 | 3.91417 | pos | N-Acetylputrescine |
| 4.24_332.1259m/z | 332.126 | 4.23673 | neg | Glutamyltryptophan |
| 4.89_433.1151m/z | 433.115 | 4.88728 | neg | (-)-epicatechin-3'-O-glucuronide |
| 4.93_244.1193m/z | 244.119 | 4.92865 | neg | Ecgonine methyl ester |
| 5.07_268.1040m/z | 268.104 | 5.07315 | pos | Deoxyguanosine |
| 5.19_186.0760m/z | 186.076 | 5.1851 | pos | 3,4-Dihydroxybenzeneacetic acid |
| 5.32_271.0597m/z | 271.06 | 5.31882 | pos | Apigenin |
| 5.41_186.0761m/z | 186.076 | 5.4097 | pos | Homogentisic acid |
| 5.75_179.0557m/z | 179.056 | 5.75143 | neg | Deoxyribose |
| 6.28_349.2027m/z | 349.203 | 6.27937 | neg | 16a-Hydroxydehydroisoandrosterone |
| 7.21_179.0709m/z | 179.071 | 7.20898 | neg | Coniferyl alcohol |
| 8.92_165.0756m/z | 165.076 | 8.92495 | pos | Galactitol |
| 9.76_349.2158m/z | 349.216 | 9.76363 | neg | Debrisoquine |
| 0.52_150.1276m/z | 150.128 | 0.51668 | pos | p-Mentha-1,3,5,8-tetraene |
| 0.54_810.5993m/z | 810.599 | 0.53792 | pos | PC(14:0/22:1(13Z)) |
| 0.70_268.1024m/z | 268.102 | 0.6975 | pos | Neuraminic acid |
| 0.70_417.0018m/z | 417.002 | 0.6975 | pos | 6-Methylthiopurine 5'-monophosphate ribonucleotide |
| 0.79_224.0234m/z | 224.023 | 0.78747 | neg | S-Carboxymethyl-L-cysteine |
| 0.94_346.0562m/z | 346.056 | 0.93742 | neg | 3'-AMP |
| 1.09_164.0380m/z | 164.038 | 1.08818 | neg | Methionine sulfoxide |
| 11.07_324.2892m/z | 324.289 | 11.0716 | pos | 8,11,14-Eicosatrienoic acid |
| 11.44_319.2284m/z | 319.228 | 11.4411 | neg | 5,6-Epoxy-8,11,14-eicosatrienoic acid |
| 13.06_813.6821m/z | 813.682 | 13.0648 | pos | SM(d18:1/24:1(15Z)) |
| 13.24_338.9892m/z | 338.989 | 13.2358 | neg | Alpha-D-Glucose 1,6-bisphosphate |
| 14.77_184.9854m/z | 184.985 | 14.7679 | pos | 3-Oxoglutaric acid |
| 3.20_247.1439m/z | 247.144 | 3.19625 | pos | Subaphylline |
| 3.44_279.1340m/z | 279.134 | 3.44418 | pos | N1-(alpha-D-ribosyl)-5,6-dimethyl-benzimidazole |
| 3.57_253.1196m/z | 253.12 | 3.57103 | neg | D-1-Piperideine-2-carboxylic acid |
| 3.86_311.1389m/z | 311.139 | 3.85608 | pos | Phenylalanyl-Tyrosine |
| 3.97_173.0814m/z | 173.081 | 3.97368 | neg | 2,3-Heptanedione |
| 4.03_162.0554m/z | 162.055 | 4.03028 | neg | Beta-Tyrosine |
| 4.23_263.0782m/z | 263.078 | 4.22672 | neg | Ethylmalonic acid |
| 4.23_385.1147m/z | 385.115 | 4.22672 | neg | trans-isoeugenol-O-glucuronide |
| 4.31_186.0760m/z | 186.076 | 4.30638 | pos | Vanillic acid |
| 4.53_260.0543m/z | 260.054 | 4.52753 | neg | Glycerylphosphorylethanolamine |
| 4.65_189.0765m/z | 189.077 | 4.65342 | neg | 4-Hydroxycyclohexylcarboxylic acid |
| 4.83_256.1193m/z | 256.119 | 4.82657 | neg | Isoproterenol |
| 4.86_124.1122m/z | 124.112 | 4.8569 | pos | p-Xylene |
| 6.55_365.2340m/z | 365.234 | 6.54503 | neg | Tetrahydrocortisol |
| 7.79_169.1228m/z | 169.123 | 7.79393 | neg | 3-Hydroxycapric acid |
| 8.10_268.1037m/z | 268.104 | 8.10235 | pos | Zidovudine |
| 8.30_310.1130m/z | 310.113 | 8.30433 | pos | N-Acetylneuraminic acid |
| 9.50_195.1389m/z | 195.139 | 9.50145 | neg | Cyclohexanone |
| 9.89_338.9893m/z | 338.989 | 9.89453 | neg | Fructose 1,6-bisphosphate |
| 0.58_143.0338m/z | 143.034 | 0.578 | pos | 3-Oxoadipic acid |
| 0.70_159.0762m/z | 159.076 | 0.6975 | pos | 2-Aminomuconic acid semialdehyde |
| 0.76_132.1018m/z | 132.102 | 0.75592 | pos | L-Isoleucine |
| 0.79_173.0561m/z | 173.056 | 0.78747 | neg | Formiminoglutamic acid |
| 0.84_134.0447m/z | 134.045 | 0.83957 | pos | Fumaric acid |
| 1.06_156.0058n | 139.002 | 1.05543 | pos | 2,5-Furandicarboxylic acid |
| 1.06_242.1132m/z | 242.113 | 1.05543 | pos | L-3-Hydroxykynurenine |
| 1.06_282.1194m/z | 282.119 | 1.05543 | pos | N6-Methyladenosine |
| 1.14_133.0608m/z | 133.061 | 1.13987 | pos | L-Asparagine |
| 1.23_257.0282m/z | 257.028 | 1.23085 | neg | Cyanuric acid |
| 1.70_192.0901n | 193.097 | 1.70135 | pos | Hydroxycotinine |
| 10.58_281.2492m/z | 281.249 | 10.582 | neg | Oleic acid |
| 11.04_293.2128m/z | 293.213 | 11.0396 | neg | 9-OxoODE |
| 11.86_554.3838m/z | 554.384 | 11.8648 | neg | LysoPC(O-18:0) |
| 13.26_399.0086m/z | 399.009 | 13.2566 | neg | D-Erythrose 4-phosphate |
| 13.39_137.0000m/z | 137 | 13.3933 | pos | 2-Furanmethanol |
| 14.89_170.0962m/z | 170.096 | 14.8945 | pos | Diphenylamine |
| 15.07_384.9938m/z | 384.994 | 15.0693 | neg | 1D-Myo-inositol 3,4-bisphosphate |
| 15.21_354.9844m/z | 354.984 | 15.2103 | neg | Ribose 1,5-bisphosphate |
| 15.52_156.0763m/z | 156.076 | 15.5157 | pos | Urocanic acid |
| 2.05_202.0717m/z | 202.072 | 2.04827 | neg | N-Acetylmannosamine |
| 3.41_267.1335m/z | 267.134 | 3.41172 | pos | Threoninyl-Phenylalanine |
| 3.57_275.1042m/z | 275.104 | 3.57103 | neg | Aspartame |
| 3.95_215.0848m/z | 215.085 | 3.9481 | pos | Methyl bisnorbiotinyl ketone |
| 4.01_175.0607m/z | 175.061 | 4.00567 | neg | 2-Isopropylmalic acid |
| 4.08_193.0504m/z | 193.05 | 4.07695 | neg | 1-Phenyl-1,2-propanedione |
| 4.50_158.0816m/z | 158.082 | 4.5008 | neg | Valerylglycine |
| 5.25_379.1208m/z | 379.121 | 5.25238 | pos | Citalopram N-oxide |
| 5.48_295.1191m/z | 295.119 | 5.48145 | neg | Ubiquinone-1 |
| 5.84_363.2184m/z | 363.218 | 5.84093 | neg | 3a,11b,21-Trihydroxy-20-oxo-5b-pregnan-18-al |
| 6.90_386.1451m/z | 386.145 | 6.90232 | pos | 3-O-Feruloylquinic acid |
| 7.30_386.1993m/z | 386.199 | 7.30173 | pos | Dehydroepiandrosterone sulfate |
| 8.16_219.1392m/z | 219.139 | 8.15733 | neg | 2-Ethyl-5-methylfuran |
| 8.81_837.5511m/z | 837.551 | 8.80973 | neg | PI(18:0/16:0) |
| 8.96_287.2235m/z | 287.223 | 8.95515 | neg | Valproic acid |
| 8.99_177.0545m/z | 177.054 | 8.99158 | pos | trans-Ferulic acid |
| 9.10_345.2077m/z | 345.208 | 9.09605 | neg | all-trans-Retinoic acid |
| 9.24_338.9893m/z | 338.989 | 9.23715 | neg | D-Fructose 2,6-bisphosphate |
| 9.65_179.0558m/z | 179.056 | 9.64592 | neg | D-Fructose |
| 9.73_107.0857m/z | 107.086 | 9.7293 | pos | Ethylbenzene |
| 0.68_203.0202m/z | 203.02 | 0.68392 | neg | 2-Maleylacetate |
| 0.70_126.0219m/z | 126.022 | 0.6975 | pos | Taurine |
| 0.76_131.0815m/z | 131.081 | 0.75592 | pos | 1-Pyrroline-2-carboxylic acid |
| 0.86_276.1189m/z | 276.119 | 0.86142 | pos | Ribothymidine |
| 0.99_133.0972m/z | 133.097 | 0.9902 | pos | D-Proline |
| 1.05_633.1278m/z | 633.128 | 1.04538 | neg | Norsanguinarine |
| 1.28_227.0784m/z | 227.078 | 1.27892 | neg | Dihydrouracil |
| 1.29_237.0868m/z | 237.087 | 1.29398 | pos | 8-Methoxykynurenate |
| 1.34_243.0273m/z | 243.027 | 1.33868 | pos | Fructose 6-phosphate |
| 1.36_128.0455m/z | 128.046 | 1.36168 | pos | 1-Methyl-4-nitroimidazole |
| 1.86_156.0421m/z | 156.042 | 1.85658 | pos | Betaine |
| 10.44_466.3886m/z | 466.389 | 10.437 | pos | 2-Deoxycastasterone |
| 10.50_325.2004m/z | 325.2 | 10.5021 | pos | N1,N12-Diacetylspermine |
| 11.01_454.3884m/z | 454.388 | 11.0062 | pos | 27-Deoxy-5b-cyprinol |
| 11.44_344.0859m/z | 344.086 | 11.4411 | neg | 8-Hydroxyguanosine |
| 11.78_329.1594m/z | 329.159 | 11.7838 | pos | N1-trans-Feruloylagmatine |
| 11.79_461.3648m/z | 461.365 | 11.7895 | neg | Gamma-Tocopherol |
| 12.29_768.5873m/z | 768.587 | 12.2856 | pos | PC(18:1(11Z)/18:1(9Z)) |
| 13.79_339.9964n | 338.989 | 13.788 | neg | D-Tagatose 1,6-bisphosphate |
| 14.83_198.0170m/z | 198.017 | 14.832 | pos | O-Phospho-4-hydroxy-L-threonine |
| 2.17_181.0971m/z | 181.097 | 2.17412 | pos | Tyrosinamide |
| 2.27_179.0557m/z | 179.056 | 2.2659 | neg | Allose |
| 2.88_107.0706m/z | 107.071 | 2.87755 | pos | 1-Methylpyrrolinium |
| 3.53_115.0755m/z | 115.076 | 3.53195 | pos | 2-Hydroxycaproic acid |
| 3.69_208.0613m/z | 208.061 | 3.6894 | neg | 3-Carbamoyl-2-phenylpropionic acid |
| 3.93_153.1273m/z | 153.127 | 3.92665 | pos | Pulegone |
| 5.15_132.0655m/z | 132.066 | 5.1451 | pos | 5-Aminolevulinic acid |
| 5.51_179.0557m/z | 179.056 | 5.5096 | neg | Alpha-D-Glucose |
| 6.95_235.1341m/z | 235.134 | 6.94823 | neg | beta-Damascenone |
| 7.31_132.0655m/z | 132.066 | 7.3147 | pos | 4-Hydroxyproline |
| 7.41_459.0944m/z | 459.094 | 7.41325 | neg | Hesperetin 7-O-glucuronide |
| 7.53_179.0557m/z | 179.056 | 7.52998 | neg | D-Tagatose |
| 7.89_179.0557m/z | 179.056 | 7.89 | neg | scyllo-Inositol |
| 8.21_179.0557m/z | 179.056 | 8.20608 | neg | L-Gulose |
| 8.34_331.1923m/z | 331.192 | 8.33827 | neg | Prostaglandin E3 |
| 8.47_347.2236m/z | 347.224 | 8.46918 | neg | Retinyl ester |
| 8.56_437.2916m/z | 437.292 | 8.5619 | neg | Isoursodeoxycholic acid |
| 9.96_107.0857m/z | 107.086 | 9.95558 | pos | m-Xylene |
| 0.52_136.0617m/z | 136.062 | 0.51668 | pos | Adenine |
| 0.80_260.1965m/z | 260.197 | 0.79692 | pos | Lysyl-Isoleucine |
| 1.56_115.0392m/z | 115.039 | 1.56287 | pos | Methylsuccinic acid |
| 1.76_115.0755m/z | 115.076 | 1.7619 | pos | 2,3-Hexanedione |
| 10.10_392.2937n | 437.292 | 10.1006 | neg | Murocholic acid |
| 10.47_179.0557m/z | 179.056 | 10.4658 | neg | L-Sorbose |
| 10.76_330.3362m/z | 330.336 | 10.7628 | pos | Phytanic acid |
| 10.83_277.2158m/z | 277.216 | 10.8285 | pos | 13-OxoODE |
| 11.05_115.0867m/z | 115.087 | 11.0497 | pos | D-Ornithine |
| 11.14_339.3002m/z | 339.3 | 11.1382 | pos | Alpha-Linolenoyl ethanolamide |
| 11.85_133.1009m/z | 133.101 | 11.8498 | pos | (+)-(S)-Carvone |
| 13.20_768.5528m/z | 768.553 | 13.1958 | pos | PC(15:0/18:1(11Z)) |
| 14.70_115.0867m/z | 115.087 | 14.7026 | pos | 3-Amino-2-piperidone |
| 2.07_186.0760m/z | 186.076 | 2.07395 | pos | p-Hydroxymandelic acid |
| 2.33_133.0496m/z | 133.05 | 2.32922 | pos | D-Xylose |
| 3.25_188.9860m/z | 188.986 | 3.24977 | neg | Pyrocatechol sulfate |
| 3.41_125.0962m/z | 125.096 | 3.41172 | pos | 2-ene-Valproic acid |
| 4.47_196.0614m/z | 196.061 | 4.46767 | neg | Acetaminophen |
| 4.73_545.0583m/z | 545.058 | 4.73262 | neg | dTDP-D-glucose |
| 4.92_175.0971m/z | 175.097 | 4.91815 | neg | Heptanoic acid |
| 4.93_186.0760m/z | 186.076 | 4.93312 | pos | 3-Hydroxymandelic acid |
| 5.11_179.0557m/z | 179.056 | 5.1123 | neg | D-chiro-inositol |
| 5.44_367.0033m/z | 367.003 | 5.4448 | neg | 6-Thiourate |
| 5.99_179.0557m/z | 179.056 | 5.98557 | neg | L-Galactose |
| 6.16_179.0557m/z | 179.056 | 6.16347 | neg | D-Mannose |
| 7.20_164.1075m/z | 164.107 | 7.20265 | neg | Pseudoephedrine |
| 7.81_143.0107m/z | 143.011 | 7.81403 | pos | 2-Hydroxybutyric acid |
| 8.05_130.0862m/z | 130.086 | 8.05135 | pos | 2-Pyrrolidineacetic acid |
| 8.66_179.0557m/z | 179.056 | 8.65785 | neg | Beta-D-Galactose |
| 9.02_392.2935n | 437.292 | 9.0241 | neg | Hyodeoxycholic acid |
| 9.71_136.0756m/z | 136.076 | 9.71215 | pos | p-Octopamine |
| 0.73_187.0711m/z | 187.071 | 0.73408 | pos | 2-Furoylglycine |
| 0.74_267.0726m/z | 267.073 | 0.74313 | neg | Ethyl glucuronide |
| 0.76_420.9702m/z | 420.97 | 0.75592 | pos | Inositol 1,3,4-trisphosphate |
| 1.06_129.1022m/z | 129.102 | 1.05543 | pos | (3S,5S)-3,5-Diaminohexanoate |
| 1.14_148.0967m/z | 148.097 | 1.13987 | pos | Adipate semialdehyde |
| 1.54_175.0867m/z | 175.087 | 1.54347 | pos | 1H-Indole-3-acetamide |
| 10.60_833.5204m/z | 833.52 | 10.5958 | neg | PI(16:1(9Z)/18:1(9Z)) |
| 11.25_193.0062m/z | 193.006 | 11.2468 | pos | Diethylthiophosphate |
| 11.90_445.3331m/z | 445.333 | 11.9024 | neg | 7a-Hydroxy-cholestene-3-one |
| 13.89_768.5529m/z | 768.553 | 13.8915 | pos | PC(20:4(5Z,8Z,11Z,14Z)/15:0) |
| 15.52_113.0711m/z | 113.071 | 15.5157 | pos | 2-Hydroxypyridine |
| 3.52_244.1291m/z | 244.129 | 3.52075 | pos | Porphobilinogen |
| 3.87_162.0761m/z | 162.076 | 3.87028 | pos | Maleic acid homopolymer |
| 4.55_151.1117m/z | 151.112 | 4.55177 | pos | (R)-Carvone |
| 6.49_129.1022m/z | 129.102 | 6.49 | pos | (3S)-3,6-Diaminohexanoate |
| 6.62_525.2828m/z | 525.283 | 6.62147 | neg | Desmosine |
| 7.20_192.1027m/z | 192.103 | 7.20265 | neg | (S)-N-Methylsalsolinol |
| 8.12_601.2653m/z | 601.265 | 8.12347 | pos | APC |
| 8.84_325.2026m/z | 325.203 | 8.83972 | neg | 12S-HHT |
| 9.27_547.0217m/z | 547.022 | 9.2652 | neg | Ferulic acid 4-O-glucuronide |
| 9.29_195.1389m/z | 195.139 | 9.29382 | neg | (Z)-3-Hexenal |
| 9.75_132.0576n | 133.065 | 9.75132 | pos | Cinnamaldehyde |
| 0.52_152.0566m/z | 152.057 | 0.51668 | pos | Guanine |
| 0.56_190.9790m/z | 190.979 | 0.556 | pos | Mercaptopurine |
| 0.72_104.0709m/z | 104.071 | 0.71572 | pos | Dimethylglycine |
| 0.78_174.0873m/z | 174.087 | 0.77695 | pos | Imidazolelactic acid |
| 0.80_146.0269m/z | 146.027 | 0.79692 | pos | Acetylcysteine |
| 1.00_187.1085m/z | 187.108 | 1.0015 | neg | N-Alpha-acetyllysine |
| 1.25_115.0392m/z | 115.039 | 1.25138 | pos | Dimethylmalonic acid |
| 1.34_183.0208n | 184.028 | 1.33868 | pos | L-Homocysteic acid |
| 1.56_153.0406m/z | 153.041 | 1.56287 | pos | Xanthine |
| 1.56_164.0685n | 165.076 | 1.56287 | pos | Rhamnose |
| 1.62_237.0632m/z | 237.063 | 1.61872 | neg | Nicotinate D-ribonucleoside |
| 10.12_315.2548m/z | 315.255 | 10.1171 | neg | Pelargonic acid |
| 10.50_395.2196m/z | 395.22 | 10.5021 | pos | Prostaglandin F1a |
| 11.30_327.2549m/z | 327.255 | 11.3036 | neg | Vaccenic acid |
| 11.30_447.3488m/z | 447.349 | 11.3036 | neg | 24-Hydroxycholesterol |
| 11.52_1074.6905m/z | 1074.69 | 11.5201 | pos | Trihexosylceramide (d18:1/18:0) |
| 11.65_126.0913m/z | 126.091 | 11.6498 | pos | o-Cresol |
| 11.88_312.2551m/z | 312.255 | 11.8849 | neg | Palmitoylglycine |
| 2.80_131.0704m/z | 131.07 | 2.8009 | pos | 3-Methyl-2-oxovaleric acid |
| 2.98_382.1012m/z | 382.101 | 2.975 | neg | Succinyladenosine |
| 3.50_158.0812m/z | 158.081 | 3.4983 | pos | 2-methoxybenzene-1,3-diol |
| 4.82_109.1015m/z | 109.101 | 4.81923 | pos | 2-Octenal |
| 5.10_165.0758m/z | 165.076 | 5.10075 | pos | Sorbitol |
| 7.91_206.0667m/z | 206.067 | 7.90562 | pos | cis-2-Methylaconitate |
| 8.88_120.0557m/z | 120.056 | 8.88085 | pos | Isoniazid |
| 0.54_759.5749n | 782.568 | 0.53792 | pos | PC(14:0/20:1(11Z)) |
| 0.54_887.5632m/z | 887.563 | 0.53792 | pos | PI(18:1(9Z)/20:3(8Z,11Z,14Z)) |
| 0.58_113.0599m/z | 113.06 | 0.578 | pos | 4-Acetylbutyrate |
| 0.68_262.0683m/z | 262.068 | 0.67747 | pos | Fucose 1-phosphate |
| 0.70_155.0424m/z | 155.042 | 0.6975 | pos | N-Carbamoylsarcosine |
| 0.70_169.9856m/z | 169.986 | 0.6975 | pos | Iminoaspartic acid |
| 0.70_209.0300m/z | 209.03 | 0.70377 | neg | Glucaric acid |
| 0.80_148.0967m/z | 148.097 | 0.79692 | pos | 2-Methyl-3-ketovaleric acid |
| 0.82_162.0760m/z | 162.076 | 0.81743 | pos | Fructosamine |
| 0.88_106.0502m/z | 106.05 | 0.88253 | pos | L-Serine |
| 1.07_160.0608m/z | 160.061 | 1.06647 | neg | Glucosamine |
| 1.70_166.0723m/z | 166.072 | 1.70135 | pos | N2-Methylguanine |
| 1.70_241.0036n | 242.011 | 1.70135 | pos | Indole-3-carboxilic acid-O-sulphate |
| 10.55_317.2130m/z | 317.213 | 10.5498 | neg | 15-KETE |
| 10.97_317.2126m/z | 317.213 | 10.9689 | neg | Leukotriene B4 |
| 2.09_244.9983m/z | 244.998 | 2.08795 | neg | Dimethylallylpyrophosphate |
| 2.23_113.0236m/z | 113.024 | 2.22818 | pos | 2-Furoic acid |
| 3.52_166.0499m/z | 166.05 | 3.52075 | pos | 4-Pyridoxolactone |
| 3.58_237.1230m/z | 237.123 | 3.58203 | pos | Phenylalanyl-Alanine |
| 3.63_157.0495m/z | 157.05 | 3.62693 | pos | Shikimic acid |
| 3.66_271.0705m/z | 271.07 | 3.65817 | neg | Hypoxanthine |
| 3.88_159.0287m/z | 159.029 | 3.88253 | pos | D-Glucurono-6,3-lactone |
| 4.03_156.1019m/z | 156.102 | 4.03185 | pos | 3-Ethyl-1,2-benzenediol |
| 4.11_158.0816m/z | 158.082 | 4.10695 | neg | N-Acetylvaline |
| 4.70_337.0428m/z | 337.043 | 4.6992 | neg | Imidazoleacetic acid ribotide |
| 4.71_165.0759m/z | 165.076 | 4.70712 | pos | Mannitol |
| 4.89_323.0272m/z | 323.027 | 4.88728 | neg | Uridine 5'-monophosphate |
| 4.97_447.0943m/z | 447.094 | 4.9729 | neg | hesperetin-7-O-glucuronide |
| 5.46_285.0409m/z | 285.041 | 5.45552 | neg | Kaempferol |
| 5.91_141.0913m/z | 141.091 | 5.91262 | neg | 5-Hydroxyvalproic acid |
| 6.66_223.1327m/z | 223.133 | 6.66378 | pos | 3-Hydroxyibuprofen |
| 7.77_166.0862m/z | 166.086 | 7.7738 | pos | trans-Cinnamic acid |
| 8.90_119.0492m/z | 119.049 | 8.90303 | pos | 2-Methylbenzoic acid |
| 9.17_391.2863m/z | 391.286 | 9.16605 | neg | Ursodeoxycholic acid |
| 9.30_311.2235m/z | 311.224 | 9.30108 | neg | 13-L-Hydroperoxylinoleic acid |
| 0.52_150.0773m/z | 150.077 | 0.51668 | pos | 3-Methyladenine |
| 0.60_622.3246m/z | 622.325 | 0.6021 | neg | (3a,5b,7a,12a)-24-[(carboxymethyl)amino]-1,12-dihydroxy-24-oxocholan-3-yl-b-D-Glucopyranosiduronic acid |
| 0.62_479.3681m/z | 479.368 | 0.61677 | pos | Galactosylsphingosine |
| 0.70_371.0244m/z | 371.024 | 0.70377 | neg | 3-Fumarylpyruvate |
| 0.73_146.0445m/z | 146.044 | 0.73408 | pos | 2-Keto-glutaramic acid |
| 0.73_189.1230m/z | 189.123 | 0.73408 | pos | Glycylleucine |
| 0.73_259.0921m/z | 259.092 | 0.73408 | pos | 3-Methyluridine |
| 0.86_331.0445m/z | 331.045 | 0.86142 | pos | Inosinic acid |
| 0.94_327.1303m/z | 327.13 | 0.93742 | neg | L-Rhamnulose |
| 0.97_138.0913m/z | 138.091 | 0.96852 | pos | 3-Methylbenzaldehyde |
| 1.07_192.0688m/z | 192.069 | 1.07472 | pos | N-Acetyl-L-methionine |
| 1.34_293.0543m/z | 293.054 | 1.33868 | pos | p-Hydroxyfelbamate |
| 1.52_176.1281m/z | 176.128 | 1.51503 | pos | 3-Oxovalproic acid |
| 1.58_183.0512m/z | 183.051 | 1.58038 | pos | 1-Methyluric acid |
| 1.69_244.9756m/z | 244.976 | 1.6933 | neg | Methylgallic acid-O-sulphate |
| 10.37_455.3175m/z | 455.318 | 10.3731 | neg | epsilon-Tocopherol |
| 10.84_361.2394m/z | 361.239 | 10.8387 | neg | Pregnenolone |
| 12.21_742.5404m/z | 742.54 | 12.2069 | neg | PC(15:0/18:2(9Z,12Z)) |
| 13.42_250.1772m/z | 250.177 | 13.4158 | pos | Isoleucyl-Threonine |
| 13.87_856.5809m/z | 856.581 | 13.8701 | pos | PC(18:1(9Z)/22:5(4Z,7Z,10Z,13Z,16Z)) |
| 2.27_240.1017m/z | 240.102 | 2.27023 | pos | L-Cystathionine |
| 2.32_211.1328m/z | 211.133 | 2.31842 | pos | Jasmonic acid |
| 3.70_113.0599m/z | 113.06 | 3.70093 | pos | 2-Ketohexanoic acid |
| 3.74_155.0449m/z | 155.045 | 3.74187 | pos | Hydantoin-5-propionic acid |
| 3.74_162.0554m/z | 162.055 | 3.73817 | neg | 4-Hydroxy-4-(3-pyridyl)-butanoic acid |
| 4.43_203.0016m/z | 203.002 | 4.42753 | neg | O-methoxycatechol-O-sulphate |
| 4.44_198.1124m/z | 198.112 | 4.43545 | pos | Propylparaben |
| 5.01_258.0389m/z | 258.039 | 5.0062 | neg | Glucosamine 6-phosphate |
| 5.30_195.0507m/z | 195.051 | 5.30188 | neg | D-Ribulose |
| 5.88_508.0743m/z | 508.074 | 5.87788 | neg | Adenylsuccinic acid |
| 5.95_201.1235m/z | 201.124 | 5.95067 | pos | Isoleucyl-Serine |
| 6.91_389.1614m/z | 389.161 | 6.9059 | neg | Tryptophyl-Tryptophan |
| 7.63_208.1332m/z | 208.133 | 7.62667 | pos | Prenyl benzoate |
| 0.56_151.0865m/z | 151.087 | 0.556 | pos | Mandelonitrile |
| 0.81_527.0548m/z | 527.055 | 0.80783 | neg | 3-Methoxy-4-Hydroxyphenylglycol sulfate |
| 0.87_147.0291m/z | 147.029 | 0.87275 | neg | Arabinonic acid |
| 0.98_464.9494m/z | 464.949 | 0.98122 | neg | Uridine triphosphate |
| 1.14_221.0665m/z | 221.067 | 1.13647 | neg | 3-Isopropylmalate |
| 1.21_115.1119m/z | 115.112 | 1.2065 | pos | 2-Heptanone |
| 1.23_244.9981m/z | 244.998 | 1.23085 | neg | Isopentenyl pyrophosphate |
| 1.57_181.0362m/z | 181.036 | 1.56782 | neg | 7-Methyluric acid |
| 1.73_115.1119m/z | 115.112 | 1.72957 | pos | Heptanal |
| 10.27_432.3129m/z | 432.313 | 10.2654 | neg | Lithocholic acid glycine conjugate |
| 12.50_794.6030m/z | 794.603 | 12.5041 | pos | PC(20:0/18:3(9Z,12Z,15Z)) |
| 2.76_149.0449m/z | 149.045 | 2.76153 | pos | L-Xylonate |
| 3.40_176.0317m/z | 176.032 | 3.39898 | pos | Mesalazine |
| 3.54_176.0317m/z | 176.032 | 3.54312 | pos | 3-Aminosalicylic acid |
| 3.58_367.1413m/z | 367.141 | 3.58018 | neg | Phosphorylcholine |
| 3.87_144.0447m/z | 144.045 | 3.86513 | neg | 4-Oxo-1-(3-pyridyl)-1-butanone |
| 4.10_418.1341m/z | 418.134 | 4.09642 | pos | 5-(3',4',5'-Trihydroxyphenyl)-gamma-valerolactone-3'-O-glucuronide |
| 4.70_155.1178m/z | 155.118 | 4.6977 | pos | Tyramine |
| 4.82_171.1021m/z | 171.102 | 4.8159 | neg | 1-Octen-3-one |
| 4.86_162.0913m/z | 162.091 | 4.8569 | pos | 1-Naphthol |
| 6.54_136.0756m/z | 136.076 | 6.54178 | pos | Vanillylamine |
| 9.74_579.0265m/z | 579.027 | 9.7394 | neg | Uridine diphosphate glucuronic acid |
| 9.97_311.2237m/z | 311.224 | 9.96982 | neg | 8(R)-Hydroperoxylinoleic acid |
| 0.62_146.1650m/z | 146.165 | 0.61677 | pos | Spermidine |
| 0.66_192.0797m/z | 192.08 | 0.6568 | pos | 2-Hydroxyiminostilbene |
| 0.68_189.1345m/z | 189.134 | 0.67747 | pos | Homo-L-arginine |
| 0.97_115.1120m/z | 115.112 | 0.96852 | pos | 5-Methyl-2-hexanone |
| 1.06_183.0288m/z | 183.029 | 1.05543 | pos | Maleylacetoacetic acid |
| 1.25_164.0341m/z | 164.034 | 1.25138 | pos | 2-Methyl-3-hydroxy-5-formylpyridine-4-carboxylate |
| 1.34_151.0614m/z | 151.061 | 1.33868 | pos | Dipropyl disulfide |
| 1.53_149.0449m/z | 149.045 | 1.53465 | pos | Ribonic acid |
| 1.70_184.0048n | 167.002 | 1.70135 | pos | 6-thiouric acid |
| 14.20_136.1120m/z | 136.112 | 14.1992 | pos | Amphetamine |
| 2.37_204.0867m/z | 204.087 | 2.37333 | pos | N-Acetyl-b-D-galactosamine |
| 3.08_204.0866m/z | 204.087 | 3.08167 | pos | N-Acetyl-D-glucosamine |
| 3.16_182.0812m/z | 182.081 | 3.15768 | pos | 2-Hydroxycinnamic acid |
| 3.80_196.0614m/z | 196.061 | 3.79528 | neg | 2-Amino-3-methylbenzoate |
| 3.95_204.0866m/z | 204.087 | 3.9481 | pos | beta-N-Acetylglucosamine |
| 4.83_335.1717m/z | 335.172 | 4.82657 | neg | Pyridoxamine |
| 5.22_149.0449m/z | 149.045 | 5.2168 | pos | D-Xylono-1,5-lactone |
| 5.53_196.0614m/z | 196.061 | 5.5314 | neg | Dopamine quinone |
| 6.07_195.0877m/z | 195.088 | 6.07078 | pos | Caffeine |
| 6.82_509.2540m/z | 509.254 | 6.82178 | pos | Ganosporeric acid A |
| 6.83_162.0913m/z | 162.091 | 6.83137 | pos | 2-Naphthol |
| 6.96_182.0812m/z | 182.081 | 6.95545 | pos | Enol-phenylpyruvate |
| 7.22_238.9950m/z | 238.995 | 7.22153 | neg | 6-Phosphonoglucono-D-lactone |
| 7.76_183.0652m/z | 183.065 | 7.75527 | pos | Homovanillic acid |
| 0.52_413.0059m/z | 413.006 | 0.51712 | neg | Alginic acid |
| 0.70_193.0349m/z | 193.035 | 0.70377 | neg | 2-Hydroxyglutarate |
| 1.06_201.0869m/z | 201.087 | 1.05543 | pos | gamma-Glutamylalanine |
| 1.12_109.0651m/z | 109.065 | 1.11803 | pos | m-Cresol |
| 1.39_232.0277m/z | 232.028 | 1.39452 | neg | Dopamine 4-sulfate |
| 1.55_198.0761m/z | 198.076 | 1.55353 | pos | L-Dopa |
| 10.52_335.2237m/z | 335.224 | 10.5225 | neg | Androsterone |
| 11.61_188.1280m/z | 188.128 | 11.6074 | pos | Pantothenol |
| 13.34_481.3333m/z | 481.333 | 13.3374 | neg | Demethylphylloquinone |
| 13.96_607.3899m/z | 607.39 | 13.9571 | pos | Zeaxanthin |
| 2.32_233.0668m/z | 233.067 | 2.32292 | neg | Deoxyinosine |
| 3.31_198.0761m/z | 198.076 | 3.31402 | pos | DL-Dopa |
| 3.75_251.1139m/z | 251.114 | 3.74853 | neg | Histidinyl-Proline |
| 4.63_214.0122m/z | 214.012 | 4.62687 | neg | 2-Amino-3-phosphonopropionic acid |
| 4.64_380.0425m/z | 380.043 | 4.63642 | pos | 6-Thioguanosine monophosphate |
| 4.85_147.0443m/z | 147.044 | 4.84987 | neg | D-Phenyllactic acid |
| 5.00_170.0367m/z | 170.037 | 5.00343 | pos | 3-Methylindole |
| 5.04_193.0860m/z | 193.086 | 5.0365 | pos | Sinapyl alcohol |
| 5.13_322.1859m/z | 322.186 | 5.13045 | pos | Arginyl-Phenylalanine |
| 6.96_137.1324m/z | 137.132 | 6.95545 | pos | D-Limonene |
| 7.13_403.0943m/z | 403.094 | 7.128 | neg | Pantetheine 4'-phosphate |
| 7.17_182.0812m/z | 182.081 | 7.17492 | pos | cis-p-Coumaric acid |
| 7.64_129.0699m/z | 129.07 | 7.63798 | pos | Naphthalene |
| 8.37_359.0968m/z | 359.097 | 8.37208 | neg | 3-Methoxy-4-hydroxyphenylglycol glucuronide |
| 8.82_115.0544m/z | 115.054 | 8.81702 | pos | Atropaldehyde |
| 0.95_155.1430m/z | 155.143 | 0.9467 | pos | Eucalyptol |
| 1.33_173.0087m/z | 173.009 | 1.3319 | neg | cis-Aconitic acid |
| 1.33_231.0455m/z | 231.045 | 1.3319 | neg | Glutamylcysteine |
| 1.34_207.0441m/z | 207.044 | 1.33868 | pos | Lanthionine ketimine |
| 1.36_165.0910m/z | 165.091 | 1.36168 | pos | 4-Isopropylbenzoic acid |
| 10.24_500.2795m/z | 500.279 | 10.2394 | neg | Fexofenadine |
| 11.69_772.5842m/z | 772.584 | 11.6942 | pos | PE(16:1(9Z)/22:1(13Z)) |
| 12.75_764.5569m/z | 764.557 | 12.7546 | neg | PC(16:0/20:3(8Z,11Z,14Z)) |
| 14.02_155.1177m/z | 155.118 | 14.0231 | pos | 4-Hydroxy-2,6-dimethylaniline |
| 14.29_135.1167m/z | 135.117 | 14.2876 | pos | p-Cymene |
| 3.61_472.1593m/z | 472.159 | 3.613 | neg | 10-Formyltetrahydrofolate |
| 4.04_258.9922m/z | 258.992 | 4.03848 | neg | Caffeic acid 3-sulfate |
| 4.33_227.0848m/z | 227.085 | 4.33058 | pos | Biotin |
| 4.64_215.1390m/z | 215.139 | 4.63642 | pos | Dethiobiotin |
| 4.94_241.0106m/z | 241.011 | 4.93947 | neg | Myo-inositol 1-phosphate |
| 5.29_165.0545m/z | 165.054 | 5.29307 | pos | 3,4-Dihydroxyhydrocinnamic acid |
| 5.30_194.0457m/z | 194.046 | 5.30188 | neg | 5,6-Dihydroxyindole |
| 5.33_243.0334m/z | 243.033 | 5.33378 | neg | 4-phenylbutanic acid-O-sulphate |
| 7.06_363.1126m/z | 363.113 | 7.0568 | neg | Farnesyl pyrophosphate |
| 8.30_349.2393m/z | 349.239 | 8.29992 | neg | Tetrahydrocorticosterone |
| 8.60_231.1127m/z | 231.113 | 8.60425 | pos | 6-Hydroxymelatonin |
| 0.79_167.0204m/z | 167.02 | 0.78747 | neg | Methyl propyl disulfide |
| 1.10_177.1022m/z | 177.102 | 1.09728 | pos | Cotinine |
| 1.18_215.1389m/z | 215.139 | 1.18395 | pos | Metanephrine |
| 10.83_599.5025m/z | 599.502 | 10.8285 | pos | DG(16:0/20:4(5Z,8Z,11Z,14Z)/0:0) |
| 13.04_743.5460n | 761.586 | 13.043 | pos | PE(14:0/22:2(13Z,16Z)) |
| 3.30_197.0808m/z | 197.081 | 3.30475 | pos | Homoveratric acid |
| 3.48_179.0702m/z | 179.07 | 3.47638 | pos | 1,2-Dihydroxy-3,4-epoxy-1,2,3,4-tetrahydronaphthalene |
| 3.57_195.1129m/z | 195.113 | 3.5682 | pos | 6-Hydroxypseudooxynicotine |
| 3.68_157.0759m/z | 157.076 | 3.67933 | pos | 3-Indoleacetonitrile |
| 4.22_155.0343m/z | 155.034 | 4.21903 | neg | 2-Isopropyl-3-oxosuccinate |
| 5.56_182.1175m/z | 182.117 | 5.55982 | pos | (3-Methylphenyl)methyl acetate |
| 6.33_317.1069m/z | 317.107 | 6.33143 | neg | Berberine |
| 0.54_196.0166m/z | 196.017 | 0.53792 | pos | 1-nitrosonaphthalene |
| 0.70_120.0656m/z | 120.066 | 0.6975 | pos | 2-Ketobutyric acid |
| 0.70_162.0400m/z | 162.04 | 0.70377 | neg | 4-Hydroxy-L-glutamic acid |
| 0.74_173.0925m/z | 173.093 | 0.74313 | neg | Valyl-Glycine |
| 0.74_174.0402m/z | 174.04 | 0.74313 | neg | N-Acetyl-L-aspartic acid |
| 0.83_511.9992m/z | 511.999 | 0.83007 | neg | 2,5-Diaminopyrimidine nucleoside triphosphate |
| 0.83_558.0045m/z | 558.004 | 0.83007 | neg | 2,5-Diamino-6-(5'-triphosphoryl-3',4'-trihydroxy-2'-oxopentyl)-amino-4-oxopyrimidine |
| 0.88_172.0401m/z | 172.04 | 0.88253 | pos | L-Methionine |
| 1.06_129.0183m/z | 129.018 | 1.05543 | pos | Oxoglutaric acid |
| 1.47_190.0862m/z | 190.086 | 1.46965 | pos | Menadione |
| 1.56_187.1085m/z | 187.108 | 1.55795 | neg | Alanyl-Valine |
| 1.79_155.1429m/z | 155.143 | 1.78613 | pos | Capric acid |
| 10.50_637.2672m/z | 637.267 | 10.5021 | pos | Coproporphyrin III |
| 11.43_183.1491m/z | 183.149 | 11.4334 | pos | Hordenine |
| 12.35_857.5201m/z | 857.52 | 12.3505 | neg | PI(20:4(5Z,8Z,11Z,14Z)/16:0) |
| 14.36_354.3361m/z | 354.336 | 14.3555 | pos | Docosadienoate (22:2n6) |
| 3.93_214.1549m/z | 214.155 | 3.92665 | pos | Gamma-Aminobutyryl-lysine |
| 4.03_304.1324m/z | 304.132 | 4.03185 | pos | Benzo[a]pyrene-7,8-diol |
| 4.44_343.1296m/z | 343.13 | 4.43545 | pos | 3-Polyprenyl-4-hydroxy-5-methoxybenzoate |
| 4.51_235.1803m/z | 235.18 | 4.51147 | pos | Lidocaine |
| 5.24_351.0184m/z | 351.018 | 5.23628 | neg | (-)-Epicatechin sulfate |
| 5.37_424.0729m/z | 424.073 | 5.36663 | pos | Gluconasturtiin |
| 6.66_213.0841m/z | 213.084 | 6.66378 | pos | Diaminopimelic acid |
| 9.16_305.1744m/z | 305.174 | 9.16295 | pos | 2-Polyprenyl-3-methyl-5-hydroxy-6-methoxy-1,4-benzoquinone |
| 9.50_579.0287m/z | 579.029 | 9.50145 | neg | UDP-D-galacturonate |
| 0.51_178.1338m/z | 178.134 | 0.5112 | pos | Tryptamine |
| 0.68_173.1037m/z | 173.104 | 0.68392 | neg | D-Arginine |
| 0.73_249.1078m/z | 249.108 | 0.73408 | pos | N2-Succinyl-L-glutamic acid 5-semialdehyde |
| 0.92_242.0787m/z | 242.079 | 0.91528 | neg | N-Acetylhistidine |
| 1.05_166.0140m/z | 166.014 | 1.04538 | neg | Quinolinic acid |
| 1.12_102.0552m/z | 102.055 | 1.11803 | pos | L-Threonine |
| 1.25_174.9915m/z | 174.991 | 1.25138 | pos | 3-Sulfinoalanine |
| 10.74_293.2130m/z | 293.213 | 10.7401 | neg | 9(S)-HPODE |
| 10.94_599.5027m/z | 599.503 | 10.9393 | pos | DG(18:2(9Z,12Z)/18:2(9Z,12Z)/0:0) |
| 11.45_433.3785m/z | 433.378 | 11.4548 | pos | Squalene |
| 12.53_490.1675m/z | 490.167 | 12.5295 | neg | Tetrahydrofolic acid |
| 3.99_166.0140m/z | 166.014 | 3.98965 | neg | 2-Amino-3-carboxymuconic acid semialdehyde |
| 3.99_303.0187m/z | 303.019 | 3.98965 | neg | 5-(3',4',5'-Trihydroxyphenyl)-gamma-valerolactone-4'-O-sulphate |
| 4.68_105.0372m/z | 105.037 | 4.67793 | pos | 3-(Methylthio)propanal |
| 5.58_331.2047m/z | 331.205 | 5.58025 | pos | Androstan-3alpha,17beta-diol |
| 1.21_212.0917m/z | 212.092 | 1.2065 | pos | Methyldopa |
| 1.53_219.0775m/z | 219.077 | 1.52885 | neg | Felbamate |
| 10.57_619.2903m/z | 619.29 | 10.5679 | neg | N-Desmethylcitalopram |
| 11.37_729.5892m/z | 729.589 | 11.3704 | pos | SM(d18:1/18:1(9Z)) |
| 11.74_856.5799m/z | 856.58 | 11.7391 | pos | PC(20:3(5Z,8Z,11Z)/20:3(8Z,11Z,14Z)) |
| 12.46_766.5719m/z | 766.572 | 12.4595 | pos | PC(16:1(9Z)/P-18:0) |
| 13.76_756.5524m/z | 756.552 | 13.7613 | pos | PC(14:1(9Z)/20:2(11Z,14Z)) |
| 3.69_163.0394m/z | 163.039 | 3.6894 | neg | 3-Methoxy-4-hydroxyphenylglycolaldehyde |
| 3.83_179.0700m/z | 179.07 | 3.82757 | pos | 4-Methoxycinnamic acid |
| 3.83_305.0344m/z | 305.034 | 3.83098 | neg | 4-Hydroxy-5-(dihydroxyphenyl)-valeric acid-O-sulphate |
| 3.99_165.0188m/z | 165.019 | 3.98965 | neg | Terephthalic acid |
| 4.88_255.0862m/z | 255.086 | 4.87918 | pos | L-Phosphoarginine |
| 7.52_186.0913m/z | 186.091 | 7.52347 | pos | N-hydroxy-4-aminobiphenyl |
| 9.14_455.2483m/z | 455.248 | 9.13955 | neg | Sulfolithocholic acid |
| 9.28_482.2956m/z | 482.296 | 9.2759 | neg | Lithocholyltaurine |
| 0.54_758.5683m/z | 758.568 | 0.53792 | pos | PC(16:1(9Z)/18:1(11Z)) |
| 0.62_103.0506m/z | 103.051 | 0.61677 | pos | Cycloserine |
| 10.87_641.2977m/z | 641.298 | 10.8715 | neg | Coproporphyrinogen III |
| 13.76_269.1619m/z | 269.162 | 13.7613 | pos | Histidinyl-Isoleucine |
| 3.70_270.0543m/z | 270.054 | 3.70147 | neg | Luteolinidin |
| 4.24_270.1912m/z | 270.191 | 4.23543 | pos | Arginyl-Isoleucine |
| 4.55_229.1434m/z | 229.143 | 4.55177 | pos | 3-Hydroxydodecanedioic acid |
| 4.64_213.1596m/z | 213.16 | 4.63642 | pos | Valyl-Leucine |
| 8.27_328.1414m/z | 328.141 | 8.2677 | pos | Arginyl-Methionine |
| 0.68_268.1629m/z | 268.163 | 0.67747 | pos | Lysyl-Valine |
| 0.70_386.9988m/z | 386.999 | 0.70377 | neg | dUDP |
| 0.70_448.9998m/z | 449 | 0.70377 | neg | Uridine 5'-diphosphate |
| 1.06_150.0543m/z | 150.054 | 1.05543 | pos | (S)-4-Hydroxymandelonitrile |
| 1.64_231.0455m/z | 231.046 | 1.64148 | neg | gamma-Glutamylcysteine |
| 10.55_561.0168m/z | 561.017 | 10.5498 | neg | UDP-L-iduronate |
| 12.83_238.9950m/z | 238.995 | 12.8255 | neg | 2-Keto-3-deoxy-6-phosphogluconic acid |
| 13.02_367.0202m/z | 367.02 | 13.0183 | neg | Orotidylic acid |
| 14.83_201.9755m/z | 201.975 | 14.832 | pos | 3-Methylsulfinylpropyl isothiocyanate |
| 4.35_582.2930m/z | 582.293 | 4.35432 | pos | LysoPC(20:4(8Z,11Z,14Z,17Z)) |
| 4.84_253.0720m/z | 253.072 | 4.84077 | neg | Arbutin |
| 6.98_200.1069m/z | 200.107 | 6.98243 | pos | 9-Hydroxyfluorene |
| 1.00_163.0394m/z | 163.039 | 1.0015 | neg | 3-(2,3-dihydroxyphenyl)propanoic acid |
| 11.41_151.1116m/z | 151.112 | 11.4124 | pos | Perillyl aldehyde |
| 11.74_796.5239m/z | 796.524 | 11.7391 | pos | PC(16:1(9Z)/18:1(9Z)) |
| 13.89_766.5717m/z | 766.572 | 13.8915 | pos | PC(18:1(9Z)/P-16:0) |
| 4.28_230.0074m/z | 230.007 | 4.28008 | neg | Phosphoserine |
| 4.53_331.1515m/z | 331.151 | 4.53305 | pos | Tyramine glucuronide |
| 4.63_306.0400n | 611.073 | 4.62687 | neg | 4-Hydroxy-5-(dihydroxyphenyl)-valeric acid-O-sulphate III |
| 4.83_233.1284m/z | 233.128 | 4.83157 | pos | Melatonin |
| 7.50_273.1484m/z | 273.148 | 7.49755 | pos | 3-Polyprenyl-4,5-dihydroxybenzoate |
| 8.85_200.2371m/z | 200.237 | 8.84922 | pos | 1-Tridecene |
| 1.09_282.0837m/z | 282.084 | 1.08818 | neg | Sepiapterin |
| 10.69_727.0392m/z | 727.039 | 10.6935 | neg | 6-Thioinosine-5'-monophosphate |
| 13.11_811.6635m/z | 811.664 | 13.1081 | pos | SM(d18:0/22:0) |
| 13.76_768.5525m/z | 768.553 | 13.7613 | pos | PC(15:0/18:1(9Z)) |
| 15.41_392.9990m/z | 392.999 | 15.4055 | neg | dIDP |
| 2.86_155.1429m/z | 155.143 | 2.86192 | pos | Alpha-Terpineol |
| 3.50_216.9805m/z | 216.98 | 3.50152 | neg | 4-hydroxybenzoic acid-4-O-sulphate |
| 9.91_198.0913m/z | 198.091 | 9.90942 | pos | Methionine sulfoximine |
| 0.54_883.5352m/z | 883.535 | 0.53828 | neg | PI(16:2(9Z,12Z)/22:3(10Z,13Z,16Z)) |
| 12.22_874.6666m/z | 874.667 | 12.2173 | neg | PC(22:2(13Z,16Z)/22:2(13Z,16Z)) |
| 3.66_159.0921m/z | 159.092 | 3.65817 | neg | Pseudooxynicotine |
| 4.25_105.0338m/z | 105.034 | 4.2506 | pos | Benzoic acid |
| 5.51_165.0188m/z | 165.019 | 5.5096 | neg | 4-O-Methylgallic acid |
| 7.02_359.0676m/z | 359.068 | 7.02335 | neg | Geranyl-PP |
| 8.72_195.0803m/z | 195.08 | 8.72052 | pos | N-Formyl-L-methionine |
| 1.80_143.9967m/z | 143.997 | 1.7971 | pos | Dimethyl trisulfide |
| 10.50_220.1178m/z | 220.118 | 10.5021 | pos | 4-ethylamino-6-isopropylamino-1,3,5-triazin-2-ol |
| 10.87_299.1479m/z | 299.148 | 10.8728 | pos | 4-Hydroxycinnamoylagmatine |
| 11.16_225.2210m/z | 225.221 | 11.1596 | pos | Pentadecanoic acid |
| 12.67_337.1664m/z | 337.166 | 12.6745 | pos | Norfloxacin |
| 4.46_653.1342m/z | 653.134 | 4.4562 | pos | CMP-N-glycoloylneuraminate |
| 4.85_235.0885m/z | 235.088 | 4.84987 | neg | Carbamazepine |
| 6.83_255.1304m/z | 255.13 | 6.83137 | pos | Leucyl-Threonine |
| 0.76_348.0695m/z | 348.07 | 0.75592 | pos | Adenosine monophosphate |
| 11.01_571.2905m/z | 571.291 | 11.0145 | neg | D-Urobilinogen |
| 12.18_856.5803m/z | 856.58 | 12.1756 | pos | PC(20:3(8Z,11Z,14Z)/20:3(8Z,11Z,14Z)) |
| 13.20_785.6504m/z | 785.65 | 13.1958 | pos | SM(d18:1/22:1(13Z)) |
| 6.66_245.1283m/z | 245.128 | 6.66378 | pos | Phenylalanylproline |
| 9.02_554.1249m/z | 554.125 | 9.0241 | neg | Biotinyl-5'-AMP |
| 0.70_425.0812m/z | 425.081 | 0.70377 | neg | Cysteineglutathione disulfide |
| 1.58_130.9664m/z | 130.966 | 1.58038 | pos | trans-3-Chloro-2-propene-1-ol |
| 10.57_470.3833m/z | 470.383 | 10.5699 | pos | 5b-Cholestane-3a,7a,12a,23R,25-pentol |
| 11.72_874.6667m/z | 874.667 | 11.7191 | neg | PC(24:0/20:4(8Z,11Z,14Z,17Z)) |
| 13.94_801.6827m/z | 801.683 | 13.9354 | pos | SM(d18:1/23:0) |
| 3.50_130.9664m/z | 130.966 | 3.4983 | pos | cis-3-Chloro-2-propene-1-ol |
| 0.68_464.9874m/z | 464.987 | 0.67747 | pos | Thymidine 5'-triphosphate |
| 0.70_199.9688m/z | 199.969 | 0.70377 | neg | Cysteine-S-sulfate |
| 12.66_494.1066m/z | 494.107 | 12.6568 | neg | Cyanidin 3-glucoside |
| 13.09_369.0357m/z | 369.036 | 13.0889 | neg | Pseudouridine 5'-phosphate |
| 0.72_300.0402m/z | 300.04 | 0.72283 | neg | N-Acetylgalactosamine 4-sulphate |
| 4.63_351.0704m/z | 351.07 | 4.62687 | neg | 7-Hydroxymethyl-12-methylbenz[a]anthracene sulfate |
| 0.62_106.0290m/z | 106.029 | 0.61677 | pos | Nicotinic acid |
| 0.79_283.1036m/z | 283.104 | 0.78747 | neg | gamma-Glutamylhistidine |
| 0.94_375.0229m/z | 375.023 | 0.93742 | neg | p-Cresol sulfate |
| 14.07_792.5869m/z | 792.587 | 14.0675 | pos | PC(18:1(9Z)/P-18:1(9Z)) |
| 4.37_255.1464m/z | 255.146 | 4.37373 | pos | Histidinyl-Valine |
| 9.15_326.2145m/z | 326.215 | 9.15012 | pos | Farnesylcysteine |
| 1.59_653.1566m/z | 653.157 | 1.58622 | neg | 2-(S-Glutathionyl)acetyl glutathione |
| 10.84_591.3509m/z | 591.351 | 10.8387 | neg | 17a-Ethynylestradiol |
| 12.50_856.5788m/z | 856.579 | 12.5041 | pos | PC(22:5(4Z,7Z,10Z,13Z,16Z)/18:1(11Z)) |
| 4.15_188.0562m/z | 188.056 | 4.14955 | neg | Glutarylglycine |
| 8.27_295.2266m/z | 295.227 | 8.2677 | pos | 11-HpODE |
| 1.36_175.0477m/z | 175.048 | 1.36168 | pos | N1-Methyl-4-pyridone-3-carboxamide |
| 10.99_750.5462m/z | 750.546 | 10.9906 | neg | PE(O-18:1(1Z)/20:4(5Z,8Z,11Z,14Z)) |
| 6.07_171.0657m/z | 171.066 | 6.07242 | neg | Diacetyl |
| 4.97_245.1285m/z | 245.128 | 4.96817 | pos | Prolylphenylalanine |
| 9.47_125.0961m/z | 125.096 | 9.471 | pos | 2,3-Octanedione |

**Table S5 Significantly differential metabolites in rat plasma between model group and vehicle groups**

| ID | m/z | Retention time (min) | Ion mode | Metabolites | Compound ID | Super Class | Class | Sub Class | VIP | P-value |
| --- | --- | --- | --- | --- | --- | --- | --- | --- | --- | --- |
| 11.51_568.3630m/z | 568.363 | 11.50967 | neg | LysoPC(18:0) | HMDB0010384 | Lipids and lipid-like molecules | Glycerophospholipids | Glycerophosphocholines | 16.4566 | 0.00064 |
| 10.39_588.3319m/z | 588.3319 | 10.38872 | neg | LysoPC(20:4(5Z,8Z,11Z,14Z)) | HMDB0010395 | Lipids and lipid-like molecules | Glycerophospholipids | Glycerophosphocholines | 13.76719 | 2.59E-05 |
| 7.87_451.2712m/z | 451.2712 | 7.866767 | neg | 7-Ketodeoxycholic acid | HMDB0000391 | Lipids and lipid-like molecules | Steroids and steroid derivatives | Bile acids, alcohols and derivatives | 0.596723 | 0.037882 |
| 10.09_493.3164n | 494.3236 | 10.09015 | pos | LysoPC(16:1(9Z)/0:0) | HMDB0010383 | Lipids and lipid-like molecules | Glycerophospholipids | Glycerophosphocholines | 2.809026 | 0.039868 |
| 9.93_382.2713m/z | 382.2713 | 9.9323 | pos | Sphinganine 1-phosphate | HMDB0001383 | Lipids and lipid-like molecules | Sphingolipids | Phosphosphingolipids | 1.315882 | 0.034014 |
| 8.65_272.2582m/z | 272.2582 | 8.65385 | pos | Palmitoleic acid | HMDB0003229 | Lipids and lipid-like molecules | Fatty Acyls | Fatty acids and conjugates | 0.59838 | 0.011763 |
| 13.24_780.5505m/z | 780.5505 | 13.23957 | pos | PC(14:0/20:2(11Z,14Z)) | HMDB0007880 | Lipids and lipid-like molecules | Glycerophospholipids | Glycerophosphocholines | 8.38551 | 0.001482 |
| 13.28_506.3604m/z | 506.3604 | 13.28398 | pos | LysoPC(P-18:1(9Z)) | HMDB0010408 | Unclassified | Unclassified | Unclassified | 0.405146 | 0.033127 |
| 7.51_449.3141n | 432.3108 | 7.510567 | pos | Deoxycholic acid glycine conjugate | HMDB0000631 | Lipids and lipid-like molecules | Steroids and steroid derivatives | Bile acids, alcohols and derivatives | 1.195265 | 0.037928 |
| 0.70_195.0506m/z | 195.0506 | 0.703767 | neg | D-Ribose | HMDB0000283 | Organic oxygen compounds | Organooxygen compounds | Carbohydrates and carbohydrate conjugates | 1.066228 | 0.012891 |
| 6.43_498.2882m/z | 498.2882 | 6.433117 | pos | Taurocholic acid | HMDB0000036 | Lipids and lipid-like molecules | Steroids and steroid derivatives | Bile acids, alcohols and derivatives | 0.668894 | 0.005182 |
| 0.70_161.1050n | 162.1122 | 0.6975 | pos | L-Carnitine | HMDB0000062 | Organic nitrogen compounds | Organonitrogen compounds | Quaternary ammonium salts | 4.628648 | 0.01572 |
| 8.82_408.2871n | 426.3209 | 8.817017 | pos | Cholic acid | HMDB0000619 | Lipids and lipid-like molecules | Steroids and steroid derivatives | Bile acids, alcohols and derivatives | 3.563427 | 0.0149 |
| 13.24_809.5922n | 832.5814 | 13.23957 | pos | PC(20:4(5Z,8Z,11Z,14Z)/18:0) | HMDB0008431 | Lipids and lipid-like molecules | Glycerophospholipids | Glycerophosphocholines | 7.966556 | 0.014678 |
| 10.46_400.3416m/z | 400.3416 | 10.45915 | pos | L-Palmitoylcarnitine | HMDB0000222 | Lipids and lipid-like molecules | Fatty Acyls | Fatty acid esters | 3.835169 | 0.00151 |
| 5.71_513.2760n | 514.2833 | 5.705417 | pos | Sulfolithocholylglycine | HMDB0002639 | Lipids and lipid-like molecules | Steroids and steroid derivatives | Bile acids, alcohols and derivatives | 1.888099 | 0.000411 |
| 6.54_515.2914n | 498.2881 | 6.541783 | pos | Tauro-b-muricholic acid | HMDB0000932 | Lipids and lipid-like molecules | Steroids and steroid derivatives | Bile acids, alcohols and derivatives | 1.274341 | 0.007874 |
| 9.97_294.2426m/z | 294.2426 | 9.971283 | pos | Stearidonic acid | HMDB0006547 | Lipids and lipid-like molecules | Fatty Acyls | Lineolic acids and derivatives | 0.459407 | 0.002867 |
| 5.05_349.2372m/z | 349.2372 | 5.04765 | pos | Cortolone | HMDB0003128 | Lipids and lipid-like molecules | Steroids and steroid derivatives | Hydroxysteroids | 0.386417 | 0.004965 |
| 10.76_431.3152m/z | 431.3152 | 10.76277 | pos | 7alpha-Hydroxy-3-oxo-4-cholestenoate | HMDB0012458 | Lipids and lipid-like molecules | Steroids and steroid derivatives | Bile acids, alcohols and derivatives | 0.885445 | 0.002779 |
| 6.77_246.2426m/z | 246.2426 | 6.771433 | pos | Myristic acid | HMDB0000806 | Lipids and lipid-like molecules | Fatty Acyls | Fatty acids and conjugates | 1.147838 | 8.52E-05 |
| 8.64_288.2895m/z | 288.2895 | 8.644367 | pos | Heptadecanoic acid | HMDB0002259 | Lipids and lipid-like molecules | Fatty Acyls | Fatty acids and conjugates | 1.384246 | 8.31E-05 |
| 2.23_118.0653m/z | 118.0653 | 2.228183 | pos | Benzeneacetonitrile | HMDB0034171 | Benzenoids | Benzene and substituted derivatives | Benzyl cyanides | 0.380725 | 1.17E-05 |
| 6.81_500.3036m/z | 500.3036 | 6.812217 | pos | Taurochenodesoxycholic acid | HMDB0000951 | Lipids and lipid-like molecules | Steroids and steroid derivatives | Bile acids, alcohols and derivatives | 0.328378 | 0.00817 |
| 0.64_146.1055n | 147.1126 | 0.6363 | pos | L-Lysine | HMDB0000182 | Organic acids and derivatives | Carboxylic acids and derivatives | Amino acids, peptides, and analogues | 2.247819 | 8.56E-07 |
| 11.41_332.0918m/z | 332.0918 | 11.41243 | pos | S-(Formylmethyl)glutathione | HMDB0060507 | Organic acids and derivatives | Carboxylic acids and derivatives | Amino acids, peptides, and analogues | 0.226122 | 0.025966 |
| 11.99_885.5514m/z | 885.5514 | 11.98792 | neg | PI(20:4(8Z,11Z,14Z,17Z)/18:0) | HMDB0009900 | Lipids and lipid-like molecules | Glycerophospholipids | Glycerophosphoinositols | 9.119381 | 0.022779 |
| 12.59_240.2319m/z | 240.2319 | 12.58927 | pos | (-)-alpha-Bisabolol | HMDB0036197 | Lipids and lipid-like molecules | Prenol lipids | Sesquiterpenoids | 1.222245 | 0.034892 |
| 11.09_548.3706m/z | 548.3706 | 11.09352 | pos | LysoPC(20:2(11Z,14Z)) | HMDB0010392 | Lipids and lipid-like molecules | Glycerophospholipids | Glycerophosphocholines | 1.010195 | 0.022516 |
| 2.42_309.1098m/z | 309.1098 | 2.4181 | neg | Phenylacetylglutamine | HMDB0006344 | Organic acids and derivatives | Carboxylic acids and derivatives | Amino acids, peptides, and analogues | 0.622082 | 2.19E-06 |
| 4.08_273.0080m/z | 273.008 | 4.07695 | neg | Ferulic acid 4-sulfate | HMDB0029200 | Phenylpropanoids and polyketides | Cinnamic acids and derivatives | Hydroxycinnamic acids and derivatives | 0.900773 | 0.001438 |
| 8.82_372.2660n | 373.2733 | 8.817017 | pos | Cervonoyl ethanolamide | HMDB0013627 | Lipids and lipid-like molecules | Fatty Acyls | Fatty acid esters | 6.023105 | 0.018783 |
| 1.01_245.0767m/z | 245.0767 | 1.011917 | pos | Uridine | HMDB0000296 | Nucleosides, nucleotides, and analogues | Pyrimidine nucleosides | Unclassified | 0.249202 | 0.01931 |
| 1.14_303.0840m/z | 303.084 | 1.136467 | neg | Imidazoleacetic acid riboside | HMDB0002331 | Nucleosides, nucleotides, and analogues | Imidazole ribonucleosides and ribonucleotides | Unclassified | 0.219669 | 0.009477 |
| 8.92_281.1016m/z | 281.1016 | 8.92495 | pos | 2-Phenylethanol glucuronide | HMDB0010350 | Organic oxygen compounds | Organooxygen compounds | Carbohydrates and carbohydrate conjugates | 0.399748 | 0.009307 |
| 9.61_211.1328m/z | 211.1328 | 9.60675 | pos | Traumatic acid | HMDB0000933 | Lipids and lipid-like molecules | Fatty Acyls | Fatty acids and conjugates | 0.39836 | 0.000266 |
| 0.68_174.0878m/z | 174.0878 | 0.683917 | neg | Citrulline | HMDB0000904 | Organic acids and derivatives | Carboxylic acids and derivatives | Amino acids, peptides, and analogues | 0.510155 | 9.16E-05 |
| 9.56_302.3051m/z | 302.3051 | 9.560117 | pos | Sphinganine | HMDB0000269 | Organic nitrogen compounds | Organonitrogen compounds | Amines | 1.180585 | 0.000379 |
| 12.48_752.5569m/z | 752.5569 | 12.48145 | pos | PE(22:2(13Z,16Z)/16:1(9Z)) | HMDB0009551 | Lipids and lipid-like molecules | Glycerophospholipids | Glycerophosphoethanolamines | 1.484626 | 0.000114 |
| 3.86_230.1022m/z | 230.1022 | 3.856083 | pos | Vanillactic acid | HMDB0000913 | Phenylpropanoids and polyketides | Phenylpropanoic acids | Unclassified | 0.410943 | 0.000141 |
| 3.87_190.0497m/z | 190.0497 | 3.870283 | pos | 4-(2-Aminophenyl)-2,4-dioxobutanoic acid | HMDB0000978 | Organic oxygen compounds | Organooxygen compounds | Carbonyl compounds | 0.326864 | 0.011936 |
| 4.56_230.9969m/z | 230.9969 | 4.555917 | neg | Hydroxymethoxyphenylcarboxylic acid-O-sulphate | HMDB0060000 | Benzenoids | Phenols | Methoxyphenols | 0.137726 | 0.000445 |
| 0.78_227.0903n | 250.0795 | 0.77695 | pos | Deoxycytidine | HMDB0000014 | Nucleosides, nucleotides, and analogues | Pyrimidine nucleosides | Pyrimidine 2'-deoxyribonucleosides | 1.283189 | 0.001461 |
| 10.23_330.3362m/z | 330.3362 | 10.23487 | pos | Arachidic acid | HMDB0002212 | Lipids and lipid-like molecules | Fatty Acyls | Fatty acids and conjugates | 0.647201 | 0.009207 |
| 0.79_243.0624m/z | 243.0624 | 0.787467 | neg | Pseudouridine | HMDB0000767 | Nucleosides, nucleotides, and analogues | Nucleoside and nucleotide analogues | Unclassified | 0.531231 | 0.001203 |
| 11.41_266.2476m/z | 266.2476 | 11.41243 | pos | (all-Z)-8,11,14-Heptadecatrienal | HMDB0041333 | Lipids and lipid-like molecules | Fatty Acyls | Fatty aldehydes | 0.48665 | 0.018639 |
| 2.41_311.1236m/z | 311.1236 | 2.40895 | pos | gamma-Glutamyltyrosine | HMDB0011741 | Organic acids and derivatives | Carboxylic acids and derivatives | Amino acids, peptides, and analogues | 0.786124 | 6.08E-06 |
| 1.52_232.1289m/z | 232.1289 | 1.515033 | pos | Asparaginyl-Valine | HMDB0028744 | Organic acids and derivatives | Carboxylic acids and derivatives | Amino acids, peptides, and analogues | 0.126162 | 0.030188 |
| 0.70_165.0397m/z | 165.0397 | 0.703767 | neg | L-Lyxonate | HMDB0060255 | Organic oxygen compounds | Organooxygen compounds | Carbohydrates and carbohydrate conjugates | 0.68734 | 0.003833 |
| 12.61_212.2005m/z | 212.2005 | 12.6103 | pos | Theaspirane | HMDB0036823 | Organoheterocyclic compounds | Tetrahydrofurans | Unclassified | 1.166337 | 0.048587 |
| 9.91_316.3207m/z | 316.3207 | 9.909417 | pos | Nonadecanoic acid | HMDB0000772 | Lipids and lipid-like molecules | Fatty Acyls | Fatty acids and conjugates | 1.771923 | 0.000941 |
| 4.97_204.0664m/z | 204.0664 | 4.9729 | neg | Indolelactic acid | HMDB0000671 | Organoheterocyclic compounds | Indoles and derivatives | Indolyl carboxylic acids and derivatives | 0.652779 | 9.49E-05 |
| 0.64_130.0862m/z | 130.0862 | 0.6363 | pos | L-Pipecolic acid | HMDB0000716 | Organic acids and derivatives | Carboxylic acids and derivatives | Amino acids, peptides, and analogues | 1.045352 | 0.000147 |
| 0.83_192.0264n | 191.0193 | 0.830067 | neg | Isocitric acid | HMDB0000193 | Organic acids and derivatives | Carboxylic acids and derivatives | Tricarboxylic acids and derivatives | 5.0044 | 2.83E-08 |
| 1.05_611.1456m/z | 611.1456 | 1.045383 | neg | Oxidized glutathione | HMDB0003337 | Organic acids and derivatives | Carboxylic acids and derivatives | Amino acids, peptides, and analogues | 0.749701 | 0.033547 |
| 12.59_170.1537m/z | 170.1537 | 12.58927 | pos | Dihydrocarvone | HMDB0036079 | Lipids and lipid-like molecules | Prenol lipids | Monoterpenoids | 0.654413 | 0.027015 |
| 9.61_318.3000m/z | 318.3 | 9.60675 | pos | Phytosphingosine | HMDB0004610 | Organic nitrogen compounds | Organonitrogen compounds | Amines | 0.299431 | 0.020401 |
| 1.12_164.0472n | 182.0811 | 1.118033 | pos | m-Coumaric acid | HMDB0001713 | Phenylpropanoids and polyketides | Cinnamic acids and derivatives | Hydroxycinnamic acids and derivatives | 3.584793 | 5.97E-05 |
| 11.23_205.0857m/z | 205.0857 | 11.22547 | pos | Monoisobutyl phthalic acid | HMDB0002056 | Benzenoids | Benzene and substituted derivatives | Benzoic acids and derivatives | 0.523678 | 0.004599 |
| 12.61_135.1167m/z | 135.1167 | 12.6103 | pos | (1S,4S)-Dihydrocarvone | HMDB0036080 | Lipids and lipid-like molecules | Prenol lipids | Monoterpenoids | 0.36599 | 0.014316 |
| 2.08_211.1076m/z | 211.1076 | 2.082917 | pos | 4-Hydroxy-5-phenyltetrahydro-1,3-oxazin-2-one | HMDB0060389 | Benzenoids | Benzene and substituted derivatives | Unclassified | 0.524089 | 0.011019 |
| 0.68_174.1115n | 175.1188 | 0.677467 | pos | L-Arginine | HMDB0000517 | Organic acids and derivatives | Carboxylic acids and derivatives | Amino acids, peptides, and analogues | 1.302957 | 0.003741 |
| 4.29_338.0888m/z | 338.0888 | 4.291917 | neg | 6-Hydroxy-5-methoxyindole glucuronide | HMDB0010362 | Organic oxygen compounds | Organooxygen compounds | Carbohydrates and carbohydrate conjugates | 0.303731 | 0.031828 |
| 1.12_135.0686n | 136.0756 | 1.118033 | pos | 2-Phenylacetamide | HMDB0010715 | Benzenoids | Benzene and substituted derivatives | Phenylacetamides | 1.507415 | 3.97E-05 |
| 5.49_301.1181m/z | 301.1181 | 5.49065 | pos | Pantetheine | HMDB0003426 | Organic acids and derivatives | Carboxylic acids and derivatives | Amino acids, peptides, and analogues | 0.402467 | 0.007235 |
| 0.78_231.1701m/z | 231.1701 | 0.77695 | pos | Valyl-Isoleucine | HMDB0029130 | Organic acids and derivatives | Carboxylic acids and derivatives | Amino acids, peptides, and analogues | 0.25069 | 0.0287 |
| 10.28_424.3416m/z | 424.3416 | 10.28005 | pos | trans-2-Tetradecenoylcarnitine | HMDB0013329 | Organic acids and derivatives | Hydroxy acids and derivatives | Beta hydroxy acids and derivatives | 1.374619 | 0.017202 |
| 1.10_180.0661m/z | 180.0661 | 1.103683 | neg | o-Tyrosine | HMDB0006050 | Organic acids and derivatives | Carboxylic acids and derivatives | Amino acids, peptides, and analogues | 0.408065 | 0.016612 |
| 0.78_112.0507m/z | 112.0507 | 0.77695 | pos | Cytosine | HMDB0000630 | Organoheterocyclic compounds | Diazines | Pyrimidines and pyrimidine derivatives | 0.763207 | 0.000399 |
| 1.97_245.1145m/z | 245.1145 | 1.967733 | neg | Glutamylvaline | HMDB0028832 | Organic acids and derivatives | Carboxylic acids and derivatives | Amino acids, peptides, and analogues | 0.233341 | 0.028042 |
| 11.35_324.2893m/z | 324.2893 | 11.34828 | pos | 11,14,17-Eicosatrienoic acid | HMDB0060039 | Lipids and lipid-like molecules | Fatty Acyls | Fatty acids and conjugates | 0.498757 | 0.000531 |
| 9.11_378.2400m/z | 378.24 | 9.109 | pos | Oleoyl glycine | HMDB0013631 | Organic acids and derivatives | Carboxylic acids and derivatives | Amino acids, peptides, and analogues | 1.137409 | 0.009713 |
| 1.12_268.1038m/z | 268.1038 | 1.118033 | pos | Adenosine | HMDB0000050 | Nucleosides, nucleotides, and analogues | Purine nucleosides | Unclassified | 0.492663 | 0.011538 |
| 1.21_221.0918m/z | 221.0918 | 1.2065 | pos | 5-Hydroxy-L-tryptophan | HMDB0000472 | Organoheterocyclic compounds | Indoles and derivatives | Tryptamines and derivatives | 0.201723 | 0.000214 |
| 1.64_261.0072m/z | 261.0072 | 1.641483 | neg | Dihydrocaffeic acid 3-sulfate | HMDB0041721 | Organic acids and derivatives | Organic sulfuric acids and derivatives | Arylsulfates | 0.431727 | 0.001048 |
| 1.06_126.0662m/z | 126.0662 | 1.055433 | pos | 5-Methylcytosine | HMDB0002894 | Organoheterocyclic compounds | Diazines | Pyrimidines and pyrimidine derivatives | 0.620607 | 0.000475 |
| 3.39_246.9920m/z | 246.992 | 3.39165 | neg | Vanillic acid 4-sulfate | HMDB0041788 | Benzenoids | Benzene and substituted derivatives | Benzoic acids and derivatives | 0.399508 | 0.000138 |
| 4.03_269.0671m/z | 269.0671 | 4.030283 | neg | Phenylglucuronide | HMDB0059806 | Organic oxygen compounds | Organooxygen compounds | Carbohydrates and carbohydrate conjugates | 0.38595 | 0.028474 |
| 11.85_627.5339m/z | 627.5339 | 11.84975 | pos | DG(18:0/20:4(5Z,8Z,11Z,14Z)/0:0) | HMDB0007170 | Lipids and lipid-like molecules | Glycerolipids | Diradylglycerols | 9.10602 | 1.25E-05 |
| 1.12_123.0441m/z | 123.0441 | 1.118033 | pos | 2-Hydroxybenzaldehyde | HMDB0034170 | Organic oxygen compounds | Organooxygen compounds | Carbonyl compounds | 0.837658 | 6.35E-05 |
| 10.28_212.2007m/z | 212.2007 | 10.28005 | pos | (3S,6E)-Nerolidol | HMDB0041629 | Lipids and lipid-like molecules | Prenol lipids | Monoterpenoids | 0.414569 | 0.02876 |
| 8.34_274.2737m/z | 274.2737 | 8.3446 | pos | Palmitic acid | HMDB0000220 | Lipids and lipid-like molecules | Fatty Acyls | Fatty acids and conjugates | 4.794773 | 0.02023 |
| 0.73_116.0707m/z | 116.0707 | 0.734083 | pos | L-Proline | HMDB0000162 | Organic acids and derivatives | Carboxylic acids and derivatives | Amino acids, peptides, and analogues | 2.148171 | 9.65E-06 |
| 13.48_729.5892m/z | 729.5892 | 13.48088 | pos | SM(d18:1/18:1(11Z)) | HMDB0012100 | Lipids and lipid-like molecules | Sphingolipids | Phosphosphingolipids | 4.928752 | 4.28E-05 |
| 4.86_206.0821m/z | 206.0821 | 4.8642 | neg | N-Acetyl-L-phenylalanine | HMDB0000512 | Organic acids and derivatives | Carboxylic acids and derivatives | Amino acids, peptides, and analogues | 0.353692 | 0.0311 |
| 0.92_243.0854n | 244.0927 | 0.924967 | pos | Cytidine | HMDB0000089 | Nucleosides, nucleotides, and analogues | Pyrimidine nucleosides | Unclassified | 0.955447 | 0.00018 |
| 12.65_552.4013m/z | 552.4013 | 12.6528 | pos | LysoPC(20:0/0:0) | HMDB0010390 | Lipids and lipid-like molecules | Glycerophospholipids | Glycerophosphocholines | 1.052481 | 0.000115 |
| 4.17_219.1127m/z | 219.1127 | 4.1738 | pos | N-Acetylserotonin | HMDB0001238 | Organoheterocyclic compounds | Indoles and derivatives | Hydroxyindoles | 0.176723 | 0.018073 |
| 1.06_258.1084m/z | 258.1084 | 1.055433 | pos | 5-Methylcytidine | HMDB0000982 | Nucleosides, nucleotides, and analogues | Pyrimidine nucleosides | Unclassified | 0.947403 | 5.08E-06 |
| 9.16_275.1638m/z | 275.1638 | 9.16295 | pos | 2-Polyprenyl-6-methoxy-1,4-benzoquinone | HMDB0060354 | Lipids and lipid-like molecules | Prenol lipids | Quinone and hydroquinone lipids | 0.458618 | 0.009988 |
| 0.80_130.0862m/z | 130.0862 | 0.796917 | pos | Pipecolic acid | HMDB0000070 | Organic acids and derivatives | Carboxylic acids and derivatives | Amino acids, peptides, and analogues | 1.270562 | 0.000107 |
| 3.59_144.0655m/z | 144.0655 | 3.591433 | pos | Aminoadipic acid | HMDB0000510 | Organic acids and derivatives | Carboxylic acids and derivatives | Amino acids, peptides, and analogues | 0.226356 | 0.003857 |
| 3.68_220.1178m/z | 220.1178 | 3.679333 | pos | Pantothenic acid | HMDB0000210 | Organic oxygen compounds | Organooxygen compounds | Alcohols and polyols | 0.291895 | 0.023497 |
| 1.61_203.0558m/z | 203.0558 | 1.612417 | neg | Succinylacetone | HMDB0000635 | Organic acids and derivatives | Keto acids and derivatives | Medium-chain keto acids and derivatives | 0.729302 | 0.018148 |
| 1.03_130.0862m/z | 130.0862 | 1.034133 | pos | D-Pipecolic acid | HMDB0005960 | Organic acids and derivatives | Carboxylic acids and derivatives | Amino acids, peptides, and analogues | 1.082859 | 0.000169 |
| 1.25_162.0527n | 145.0495 | 1.251383 | pos | 2-Hydroxyadipic acid | HMDB0000321 | Lipids and lipid-like molecules | Fatty Acyls | Fatty acids and conjugates | 2.610687 | 1.82E-05 |
| 10.85_282.2788m/z | 282.2788 | 10.8505 | pos | 3-Dehydrosphinganine | HMDB0001480 | Organic oxygen compounds | Organooxygen compounds | Carbonyl compounds | 0.450379 | 0.018352 |
| 3.88_114.0915m/z | 114.0915 | 3.882533 | pos | Aminocaproic acid | HMDB0001901 | Lipids and lipid-like molecules | Fatty Acyls | Fatty acids and conjugates | 1.154542 | 6.84E-07 |
| 4.26_178.0504m/z | 178.0504 | 4.259983 | neg | Hippuric acid | HMDB0000714 | Benzenoids | Benzene and substituted derivatives | Benzoic acids and derivatives | 1.057322 | 0.001023 |
| 4.85_165.0551m/z | 165.0551 | 4.849867 | neg | Homovanillin | HMDB0005175 | Benzenoids | Phenols | Methoxyphenols | 0.386015 | 0.004301 |
| 1.05_192.0264n | 191.0193 | 1.045383 | neg | Diketogulonic acid | HMDB0005971 | Organic oxygen compounds | Organooxygen compounds | Carbohydrates and carbohydrate conjugates | 4.798133 | 4.92E-08 |
| 5.31_218.2114m/z | 218.2114 | 5.307667 | pos | Dodecanoic acid | HMDB0000638 | Lipids and lipid-like molecules | Fatty Acyls | Fatty acids and conjugates | 2.379709 | 1.66E-06 |
| 0.73_146.1173m/z | 146.1173 | 0.734083 | pos | Pantolactone | HMDB0059876 | Organoheterocyclic compounds | Lactones | Gamma butyrolactones | 0.854896 | 0.017568 |
| 1.16_143.0338m/z | 143.0338 | 1.1621 | pos | Oxoadipic acid | HMDB0000225 | Organic acids and derivatives | Keto acids and derivatives | Medium-chain keto acids and derivatives | 0.731722 | 0.000184 |
| 8.25_185.1180m/z | 185.118 | 8.245317 | neg | 2-Nonenal | HMDB0031269 | Organic oxygen compounds | Organooxygen compounds | Carbonyl compounds | 0.625348 | 0.045171 |
| 8.92_105.0702m/z | 105.0702 | 8.92495 | pos | 2-Phenylethanol | HMDB0033944 | Benzenoids | Benzene and substituted derivatives | Unclassified | 0.426602 | 0.000465 |
| 0.73_102.0552m/z | 102.0552 | 0.734083 | pos | L-Homoserine | HMDB0000719 | Organic acids and derivatives | Carboxylic acids and derivatives | Amino acids, peptides, and analogues | 0.345248 | 0.007666 |
| 1.03_127.0390m/z | 127.039 | 1.034133 | pos | Maltol | HMDB0030776 | Organoheterocyclic compounds | Pyrans | Pyranones and derivatives | 2.125575 | 2.1E-05 |
| 12.61_156.1381m/z | 156.1381 | 12.6103 | pos | 2-Pentylfuran | HMDB0013824 | Organoheterocyclic compounds | Heteroaromatic compounds | Unclassified | 0.526039 | 0.025014 |
| 5.41_190.0498m/z | 190.0498 | 5.4097 | pos | Kynurenic acid | HMDB0000715 | Organoheterocyclic compounds | Quinolines and derivatives | Quinoline carboxylic acids | 0.521998 | 0.000212 |
| 0.72_132.0655m/z | 132.0655 | 0.715717 | pos | L-Glutamic gamma-semialdehyde | HMDB0002104 | Organic acids and derivatives | Carboxylic acids and derivatives | Amino acids, peptides, and analogues | 1.1076 | 0.00168 |
| 0.74_157.0359m/z | 157.0359 | 0.743133 | neg | Allantoic acid | HMDB0001209 | Organic acids and derivatives | Carboxylic acids and derivatives | Amino acids, peptides, and analogues | 0.5786 | 0.012775 |
| 11.94_300.2896m/z | 300.2896 | 11.93697 | pos | Elaidic acid | HMDB0000573 | Lipids and lipid-like molecules | Fatty Acyls | Fatty acids and conjugates | 0.236773 | 0.013617 |
| 4.79_173.0814m/z | 173.0814 | 4.791317 | neg | Cyclohexanecarboxylic acid | HMDB0031342 | Organic acids and derivatives | Carboxylic acids and derivatives | Carboxylic acids | 1.116539 | 0.007104 |
| 1.36_153.0658m/z | 153.0658 | 1.361683 | pos | N1-Methyl-2-pyridone-5-carboxamide | HMDB0004193 | Organoheterocyclic compounds | Pyridines and derivatives | Pyridinecarboxylic acids and derivatives | 1.017156 | 0.010402 |
| 3.99_242.9971m/z | 242.9971 | 3.98965 | neg | 3-hydroxy-3-(3-hydroxyphenyl)propanoic acid-O-sulphate | HMDB0059967 | Organic acids and derivatives | Hydroxy acids and derivatives | Beta hydroxy acids and derivatives | 0.903951 | 3.83E-05 |
| 1.12_136.0526n | 119.0493 | 1.118033 | pos | Phenylacetic acid | HMDB0000209 | Benzenoids | Benzene and substituted derivatives | Unclassified | 0.582007 | 0.000187 |
| 10.87_104.1071m/z | 104.1071 | 10.8728 | pos | (E)-2-Penten-1-ol | HMDB0031604 | Organic oxygen compounds | Organooxygen compounds | Alcohols and polyols | 0.234024 | 3.99E-07 |
| 13.76_267.2680m/z | 267.268 | 13.76133 | pos | Stearic acid | HMDB0000827 | Lipids and lipid-like molecules | Fatty Acyls | Fatty acids and conjugates | 1.179984 | 7.55E-06 |
| 2.21_164.0710m/z | 164.071 | 2.214233 | neg | L-Phenylalanine | HMDB0000159 | Organic acids and derivatives | Carboxylic acids and derivatives | Amino acids, peptides, and analogues | 0.550222 | 0.000267 |
| 3.87_228.0877m/z | 228.0877 | 3.865133 | neg | Epinephrine | HMDB0000068 | Benzenoids | Phenols | Benzenediols | 0.466272 | 0.001819 |
| 7.13_214.1800m/z | 214.18 | 7.128983 | pos | alpha-Terpineol acetate | HMDB0032051 | Lipids and lipid-like molecules | Prenol lipids | Monoterpenoids | 0.554008 | 0.001031 |
| 0.68_188.1522n | 189.1595 | 0.677467 | pos | N6,N6,N6-Trimethyl-L-lysine | HMDB0001325 | Organic acids and derivatives | Carboxylic acids and derivatives | Amino acids, peptides, and analogues | 1.554776 | 3.37E-06 |
| 0.70_175.0955n | 198.0847 | 0.6975 | pos | Argininic acid | HMDB0003148 | Organic acids and derivatives | Carboxylic acids and derivatives | Amino acids, peptides, and analogues | 1.437045 | 2.33E-05 |
| 9.16_338.2321m/z | 338.2321 | 9.16295 | pos | 5'-Carboxy-alpha-chromanol | HMDB0012798 | Organoheterocyclic compounds | Benzopyrans | 1-benzopyrans | 0.389417 | 0.005474 |
| 10.26_448.3417m/z | 448.3417 | 10.25752 | pos | LysoSM(d18:1) | HMDB0006482 | Lipids and lipid-like molecules | Sphingolipids | Phosphosphingolipids | 0.60045 | 0.014721 |
| 7.20_283.1691m/z | 283.1691 | 7.203533 | pos | 2-Methoxyestrone | HMDB0000010 | Lipids and lipid-like molecules | Steroids and steroid derivatives | Estrane steroids | 0.127151 | 0.012252 |
| 8.86_288.2894m/z | 288.2894 | 8.8619 | pos | Methyl hexadecanoic acid | HMDB0061859 | Lipids and lipid-like molecules | Fatty Acyls | Fatty acid esters | 1.755232 | 0.000723 |
| 0.76_174.1004n | 175.1076 | 0.755917 | pos | Glycyl-Valine | HMDB0028854 | Organic acids and derivatives | Carboxylic acids and derivatives | Amino acids, peptides, and analogues | 0.69552 | 1.49E-05 |
| 10.67_104.1071m/z | 104.1071 | 10.67483 | pos | Pentanal | HMDB0031206 | Organic oxygen compounds | Organooxygen compounds | Carbonyl compounds | 0.318945 | 1.51E-08 |
| 4.46_447.2067m/z | 447.2067 | 4.45855 | neg | 1-Methyl-6-phenyl-1H-imidazo[4,5-b]pyridin-2-amine | HMDB0041008 | Organoheterocyclic compounds | Pyridines and derivatives | Phenylpyridines | 0.885454 | 0.006865 |
| 5.58_514.2854m/z | 514.2854 | 5.581033 | neg | Taurohyocholate | HMDB0011637 | Lipids and lipid-like molecules | Steroids and steroid derivatives | Bile acids, alcohols and derivatives | 1.525406 | 0.024068 |
| 0.64_155.0693n | 156.0766 | 0.6363 | pos | L-Histidine | HMDB0000177 | Organic acids and derivatives | Carboxylic acids and derivatives | Amino acids, peptides, and analogues | 0.769719 | 5.35E-05 |
| 0.80_219.0973m/z | 219.0973 | 0.796917 | pos | Alanyl-Glutamic acid | HMDB0028686 | Organic acids and derivatives | Carboxylic acids and derivatives | Amino acids, peptides, and analogues | 0.451062 | 2.11E-05 |
| 0.68_381.0251m/z | 381.0251 | 0.677467 | pos | 6-Thioxanthine 5'-monophosphate | HMDB0060418 | Organoheterocyclic compounds | Diazines | Pyrimidines and pyrimidine derivatives | 0.965381 | 0.00288 |
| 11.94_199.1690m/z | 199.169 | 11.93697 | pos | 3-Hydroxydodecanoic acid | HMDB0000387 | Organic acids and derivatives | Hydroxy acids and derivatives | Medium-chain hydroxy acids and derivatives | 0.186604 | 0.03462 |
| 2.17_126.0550m/z | 126.055 | 2.174117 | pos | Quinone | HMDB0003364 | Organic oxygen compounds | Organooxygen compounds | Carbonyl compounds | 0.135327 | 0.007914 |
| 2.37_121.0649m/z | 121.0649 | 2.373333 | pos | Phenylacetaldehyde | HMDB0006236 | Benzenoids | Benzene and substituted derivatives | Phenylacetaldehydes | 0.120577 | 0.042336 |
| 2.73_216.9811m/z | 216.9811 | 2.729317 | neg | 3-hydroxybenzoic acid-3-O-sulphate | HMDB0059968 | Organic acids and derivatives | Organic sulfuric acids and derivatives | Arylsulfates | 0.445668 | 0.001287 |
| 3.91_142.0862m/z | 142.0862 | 3.914167 | pos | Betonicine | HMDB0029412 | Organic acids and derivatives | Carboxylic acids and derivatives | Amino acids, peptides, and analogues | 0.820119 | 0.000309 |
| 1.01_209.0556m/z | 209.0556 | 1.011917 | pos | 3-Nitrotyrosine | HMDB0001904 | Organic acids and derivatives | Carboxylic acids and derivatives | Amino acids, peptides, and analogues | 0.146479 | 0.038375 |
| 1.01_217.1293m/z | 217.1293 | 1.011917 | pos | N-a-Acetyl-L-arginine | HMDB0004620 | Organic acids and derivatives | Carboxylic acids and derivatives | Amino acids, peptides, and analogues | 0.155963 | 0.024248 |
| 10.23_297.2665n | 280.2632 | 10.23487 | pos | Palmitoleoyl Ethanolamide | HMDB0013648 | Organic nitrogen compounds | Organonitrogen compounds | Amines | 2.088839 | 0.020342 |
| 11.54_104.1072m/z | 104.1072 | 11.5425 | pos | 3-Methyl-3-buten-1-ol | HMDB0030126 | Organic oxygen compounds | Organooxygen compounds | Alcohols and polyols | 0.380439 | 7.2E-06 |
| 3.43_185.1284m/z | 185.1284 | 3.426317 | pos | Alanyl-Isoleucine | HMDB0028690 | Organic acids and derivatives | Carboxylic acids and derivatives | Amino acids, peptides, and analogues | 0.621552 | 0.003297 |
| 2.21_107.0494m/z | 107.0494 | 2.2062 | pos | Benzaldehyde | HMDB0006115 | Benzenoids | Benzene and substituted derivatives | Benzoyl derivatives | 0.161542 | 1.76E-05 |
| 5.09_188.1645m/z | 188.1645 | 5.086 | pos | 8-Methylnonenoate | HMDB0012183 | Lipids and lipid-like molecules | Fatty Acyls | Fatty acids and conjugates | 0.149178 | 0.010922 |
| 8.92_120.0577n | 121.0647 | 8.92495 | pos | 2-Methylbenzaldehyde | HMDB0029636 | Benzenoids | Benzene and substituted derivatives | Benzoyl derivatives | 0.334142 | 0.001461 |
| 1.28_276.0180m/z | 276.018 | 1.278917 | neg | Paracetamol sulfate | HMDB0059911 | Organic acids and derivatives | Organic sulfuric acids and derivatives | Arylsulfates | 0.499124 | 0.003061 |
| 2.23_148.0524n | 166.0862 | 2.228183 | pos | (E)-3-(4-Hydroxyphenyl)-2-propenal | HMDB0040986 | Phenylpropanoids and polyketides | Cinnamaldehydes | Unclassified | 5.290475 | 9.36E-07 |
| 3.43_126.0914m/z | 126.0914 | 3.426317 | pos | Proline betaine | HMDB0004827 | Organic acids and derivatives | Carboxylic acids and derivatives | Amino acids, peptides, and analogues | 0.205709 | 0.003135 |
| 0.97_231.1701m/z | 231.1701 | 0.968517 | pos | Leucyl-Valine | HMDB0028942 | Organic acids and derivatives | Carboxylic acids and derivatives | Amino acids, peptides, and analogues | 0.228133 | 0.008037 |
| 12.48_731.6049m/z | 731.6049 | 12.48145 | pos | SM(d18:0/18:1(11Z)) | HMDB0012088 | Lipids and lipid-like molecules | Sphingolipids | Phosphosphingolipids | 6.651656 | 0.001395 |
| 13.04_328.3205m/z | 328.3205 | 13.04303 | pos | 11Z-Eicosenoic acid | HMDB0002231 | Lipids and lipid-like molecules | Fatty Acyls | Fatty acids and conjugates | 0.272101 | 0.010599 |
| 3.37_159.0656m/z | 159.0656 | 3.369717 | neg | 3-Methyladipic acid | HMDB0000555 | Lipids and lipid-like molecules | Fatty Acyls | Fatty acids and conjugates | 0.207164 | 0.046756 |
| 2.01_171.0657m/z | 171.0657 | 2.009183 | neg | Gamma-Butyrolactone | HMDB0000549 | Organoheterocyclic compounds | Lactones | Gamma butyrolactones | 0.275811 | 0.024604 |
| 4.51_181.0859m/z | 181.0859 | 4.511467 | pos | 3-Methoxybenzenepropanoic acid | HMDB0011751 | Phenylpropanoids and polyketides | Phenylpropanoic acids | Unclassified | 0.373459 | 0.004741 |
| 1.05_316.1154m/z | 316.1154 | 1.045383 | neg | Aspartylglycosamine | HMDB0000489 | Organic oxygen compounds | Organooxygen compounds | Carbohydrates and carbohydrate conjugates | 0.504914 | 0.001688 |
| 1.45_182.0454m/z | 182.0454 | 1.45255 | neg | 2-Aminobenzoic acid | HMDB0001123 | Benzenoids | Benzene and substituted derivatives | Benzoic acids and derivatives | 0.187392 | 0.028511 |
| 10.39_356.3519m/z | 356.3519 | 10.39185 | pos | Erucic acid | HMDB0002068 | Lipids and lipid-like molecules | Fatty Acyls | Fatty acids and conjugates | 0.26294 | 0.023142 |
| 9.96_111.1170m/z | 111.117 | 9.955583 | pos | (R)-1-Octen-3-ol | HMDB0031299 | Lipids and lipid-like molecules | Fatty Acyls | Fatty alcohols | 0.244288 | 0.001031 |
| 10.21_149.0960m/z | 149.096 | 10.21247 | pos | Perillic acid | HMDB0004586 | Lipids and lipid-like molecules | Prenol lipids | Monoterpenoids | 0.386094 | 0.001171 |
| 12.15_326.3047m/z | 326.3047 | 12.15347 | pos | Eicosadienoic acid | HMDB0005060 | Lipids and lipid-like molecules | Fatty Acyls | Fatty acids and conjugates | 0.314467 | 0.01641 |
| 4.55_186.1131m/z | 186.1131 | 4.5452 | neg | (±)-Pelletierine | HMDB0030325 | Organoheterocyclic compounds | Piperidines | Unclassified | 0.948574 | 2.85E-05 |
| 7.86_405.2655m/z | 405.2655 | 7.856083 | neg | 3a,6b,7a,12a-Tetrahydroxy-5b-cholanoic acid | HMDB0000399 | Organic oxygen compounds | Organooxygen compounds | Carbohydrates and carbohydrate conjugates | 1.537074 | 0.000123 |
| 9.07_338.9893m/z | 338.9893 | 9.0664 | neg | D-Glyceraldehyde 3-phosphate | HMDB0001112 | Organic oxygen compounds | Organooxygen compounds | Carbohydrates and carbohydrate conjugates | 0.325889 | 0.000981 |
| 9.07_361.1641m/z | 361.1641 | 9.0664 | neg | Thyrotropin releasing hormone | HMDB0060080 | Organic acids and derivatives | Carboxylic acids and derivatives | Amino acids, peptides, and analogues | 0.571076 | 1.54E-06 |
| 0.72_160.0602m/z | 160.0602 | 0.715717 | pos | Sumiki's acid | HMDB0002432 | Organoheterocyclic compounds | Furans | Furoic acid and derivatives | 1.214473 | 0.033724 |
| 12.02_223.2054m/z | 223.2054 | 12.02277 | pos | Farnesol | HMDB0004305 | Lipids and lipid-like molecules | Prenol lipids | Sesquiterpenoids | 0.311019 | 0.049184 |
| 12.50_796.5232m/z | 796.5232 | 12.50405 | pos | PC(14:1(9Z)/20:1(11Z)) | HMDB0007912 | Lipids and lipid-like molecules | Glycerophospholipids | Glycerophosphocholines | 1.590881 | 0.000351 |
| 3.63_198.0527n | 181.0494 | 3.626933 | pos | Vanillylmandelic acid | HMDB0000291 | Benzenoids | Phenols | Methoxyphenols | 0.463845 | 0.047382 |
| 5.29_144.0655m/z | 144.0655 | 5.293067 | pos | Methyl 2-furoate | HMDB0029750 | Organoheterocyclic compounds | Furans | Furoic acid and derivatives | 0.129461 | 0.008167 |
| 1.06_192.0268n | 215.016 | 1.055433 | pos | Citric acid | HMDB0000094 | Organic acids and derivatives | Carboxylic acids and derivatives | Tricarboxylic acids and derivatives | 1.974059 | 7.5E-09 |
| 11.69_794.6033m/z | 794.6033 | 11.69422 | pos | PC(20:1(11Z)/P-16:0) | HMDB0008324 | Lipids and lipid-like molecules | Glycerophospholipids | Glycerophosphocholines | 3.189605 | 0.019217 |
| 13.20_152.9947m/z | 152.9947 | 13.19578 | pos | Dihydroxyacetone phosphate | HMDB0001473 | Organic oxygen compounds | Organooxygen compounds | Carbohydrates and carbohydrate conjugates | 3.556277 | 3.01E-05 |
| 13.24_768.5870m/z | 768.587 | 13.23957 | pos | PC(18:1(11Z)/18:1(11Z)) | HMDB0008070 | Lipids and lipid-like molecules | Glycerophospholipids | Glycerophosphocholines | 5.089688 | 0.012019 |
| 0.70_104.1072m/z | 104.1072 | 0.6975 | pos | 1-Penten-3-ol | HMDB0031605 | Organic oxygen compounds | Organooxygen compounds | Alcohols and polyols | 0.800144 | 0.019846 |
| 0.70_137.0708m/z | 137.0708 | 0.6975 | pos | N-Methylnicotinamide | HMDB0003152 | Organoheterocyclic compounds | Pyridines and derivatives | Pyridinecarboxylic acids and derivatives | 0.640918 | 0.00129 |
| 1.15_203.0558m/z | 203.0558 | 1.14835 | neg | Acetoacetic acid | HMDB0000060 | Organic acids and derivatives | Keto acids and derivatives | Short-chain keto acids and derivatives | 0.268965 | 0.002319 |
| 1.23_176.9718m/z | 176.9718 | 1.228717 | pos | Fosfomycin | HMDB0014966 | Organic acids and derivatives | Organic phosphonic acids and derivatives | Organic phosphonic acids | 0.486824 | 6.22E-05 |
| 1.36_153.0426n | 154.0498 | 1.361683 | pos | 3-Hydroxyanthranilic acid | HMDB0001476 | Benzenoids | Benzene and substituted derivatives | Benzoic acids and derivatives | 0.901163 | 0.014264 |
| 4.56_151.0393m/z | 151.0393 | 4.555917 | neg | 3,4-Dihydroxyphenylglycol | HMDB0000318 | Benzenoids | Phenols | Benzenediols | 0.094937 | 1.39E-05 |
| 1.06_123.0554m/z | 123.0554 | 1.055433 | pos | Pi-Methylimidazoleacetic acid | HMDB0004988 | Organoheterocyclic compounds | Azoles | Imidazoles | 1.629552 | 2.43E-08 |
| 10.23_364.2479m/z | 364.2479 | 10.23487 | pos | 21-Hydroxy-5b-pregnane-3,11,20-trione | HMDB0006756 | Lipids and lipid-like molecules | Steroids and steroid derivatives | Hydroxysteroids | 0.137158 | 0.013717 |
| 13.24_794.6031m/z | 794.6031 | 13.23957 | pos | PC(20:0/18:3(6Z,9Z,12Z)) | HMDB0008271 | Lipids and lipid-like molecules | Glycerophospholipids | Glycerophosphocholines | 4.698304 | 0.006446 |
| 4.70_163.0394m/z | 163.0394 | 4.6992 | neg | Phenylpyruvic acid | HMDB0000205 | Benzenoids | Benzene and substituted derivatives | Phenylpyruvic acid derivatives | 0.411629 | 0.000153 |
| 4.85_193.0495m/z | 193.0495 | 4.845917 | pos | Vanilpyruvic acid | HMDB0011714 | Benzenoids | Benzene and substituted derivatives | Phenylpyruvic acid derivatives | 0.279846 | 0.00176 |
| 9.63_260.1278m/z | 260.1278 | 9.62775 | pos | Equol | HMDB0002209 | Phenylpropanoids and polyketides | Isoflavonoids | Isoflavans | 0.172329 | 0.018278 |
| 0.78_305.0842m/z | 305.0842 | 0.77695 | pos | Xylobiose | HMDB0029894 | Organic oxygen compounds | Organooxygen compounds | Carbohydrates and carbohydrate conjugates | 0.767025 | 0.019957 |
| 10.89_289.1560m/z | 289.156 | 10.89498 | pos | Normorphine | HMDB0041959 | Alkaloids and derivatives | Morphinans | Unclassified | 0.556324 | 9.61E-06 |
| 10.96_286.3100m/z | 286.31 | 10.96143 | pos | Stearaldehyde | HMDB0002384 | Lipids and lipid-like molecules | Fatty Acyls | Fatty aldehydes | 1.540817 | 0.028295 |
| 11.23_121.0285m/z | 121.0285 | 11.22547 | pos | 4-Hydroxybenzoic acid | HMDB0000500 | Benzenoids | Benzene and substituted derivatives | Benzoic acids and derivatives | 0.141356 | 0.007266 |
| 3.88_108.0811m/z | 108.0811 | 3.882533 | pos | Benzylamine | HMDB0033871 | Benzenoids | Benzene and substituted derivatives | Phenylmethylamines | 0.217978 | 0.006177 |
| 4.90_172.0973m/z | 172.0973 | 4.8964 | neg | Hexanoylglycine | HMDB0000701 | Organic acids and derivatives | Carboxylic acids and derivatives | Amino acids, peptides, and analogues | 0.269951 | 0.004295 |
| 5.26_343.2590m/z | 343.259 | 5.2613 | pos | 10-Nitrolinoleic acid | HMDB0005049 | Lipids and lipid-like molecules | Fatty Acyls | Lineolic acids and derivatives | 0.68843 | 0.00017 |
| 0.79_300.0942m/z | 300.0942 | 0.787467 | neg | 7,8-Dihydroneopterin | HMDB0002275 | Organoheterocyclic compounds | Pteridines and derivatives | Pterins and derivatives | 0.493655 | 0.046823 |
| 4.32_174.0885n | 173.0814 | 4.315217 | neg | Suberic acid | HMDB0000893 | Lipids and lipid-like molecules | Fatty Acyls | Fatty acids and conjugates | 0.411906 | 0.031827 |
| 6.05_123.0805m/z | 123.0805 | 6.052767 | pos | 4-Ethylphenol | HMDB0029306 | Benzenoids | Phenols | 1-hydroxy-2-unsubstituted benzenoids | 0.284348 | 0.002855 |
| 8.42_230.2477m/z | 230.2477 | 8.420017 | pos | 2-Tetradecanone | HMDB0030924 | Organic oxygen compounds | Organooxygen compounds | Carbonyl compounds | 1.371745 | 0.001756 |
| 10.07_442.3522m/z | 442.3522 | 10.06787 | pos | 2,3-Diaminosalicylic acid | HMDB0013159 | Benzenoids | Benzene and substituted derivatives | Benzoic acids and derivatives | 0.801064 | 0.017225 |
| 11.18_127.0866m/z | 127.0866 | 11.18158 | pos | 4-Aminophenol | HMDB0001169 | Benzenoids | Benzene and substituted derivatives | Aniline and substituted anilines | 0.068096 | 0.038313 |
| 13.20_203.0215m/z | 203.0215 | 13.19578 | pos | Imidazole acetol-phosphate | HMDB0012236 | Organic acids and derivatives | Organic phosphoric acids and derivatives | Phosphate esters | 0.400719 | 0.000129 |
| 2.56_204.0267m/z | 204.0267 | 2.560467 | pos | 2-Phosphoglyceric acid | HMDB0000362 | Organic oxygen compounds | Organooxygen compounds | Carbohydrates and carbohydrate conjugates | 0.072392 | 0.014031 |
| 3.63_168.0655m/z | 168.0655 | 3.626933 | pos | Pyridoxal | HMDB0001545 | Organoheterocyclic compounds | Pyridines and derivatives | Pyridine carboxaldehydes | 0.135417 | 0.005242 |
| 4.65_187.0971m/z | 187.0971 | 4.653417 | neg | cis-3-Hexenyl acetate | HMDB0040215 | Organic acids and derivatives | Carboxylic acids and derivatives | Carboxylic acid derivatives | 0.22622 | 0.024243 |
| 4.87_208.0968m/z | 208.0968 | 4.866183 | pos | Phenylpropionylglycine | HMDB0000860 | Organic acids and derivatives | Carboxylic acids and derivatives | Amino acids, peptides, and analogues | 0.252457 | 0.005998 |
| 5.18_189.1128m/z | 189.1128 | 5.1824 | neg | Caprylic acid | HMDB0000482 | Lipids and lipid-like molecules | Fatty Acyls | Fatty acids and conjugates | 0.264184 | 9.5E-06 |
| 0.56_172.9961m/z | 172.9961 | 0.556 | pos | (S)-Ureidoglycolic acid | HMDB0001005 | Organic acids and derivatives | Carboxylic acids and derivatives | Amino acids, peptides, and analogues | 0.48348 | 9.78E-07 |
| 1.12_121.0649m/z | 121.0649 | 1.118033 | pos | Tyrosol | HMDB0004284 | Benzenoids | Phenols | Tyrosols and derivatives | 0.20334 | 0.003439 |
| 1.34_228.0715m/z | 228.0715 | 1.338683 | pos | Galactaric acid | HMDB0000639 | Organic oxygen compounds | Organooxygen compounds | Carbohydrates and carbohydrate conjugates | 0.09133 | 0.024745 |
| 13.72_101.0713m/z | 101.0713 | 13.71755 | pos | L-2,4-diaminobutyric acid | HMDB0006284 | Organic acids and derivatives | Carboxylic acids and derivatives | Amino acids, peptides, and analogues | 0.089567 | 0.029546 |
| 2.61_225.0880m/z | 225.088 | 2.612433 | neg | 5-Hydroxykynurenamine | HMDB0004076 | Organic oxygen compounds | Organooxygen compounds | Carbonyl compounds | 0.145777 | 0.034611 |
| 5.18_204.0664m/z | 204.0664 | 5.1824 | neg | N-Hydroxy-1-aminonaphthalene | HMDB0062503 | Benzenoids | Naphthalenes | Unclassified | 0.206142 | 0.001593 |
| 8.88_244.2632m/z | 244.2632 | 8.88085 | pos | Pentadecanal | HMDB0031078 | Lipids and lipid-like molecules | Fatty Acyls | Fatty aldehydes | 0.325247 | 0.001499 |
| 0.58_127.0390m/z | 127.039 | 0.578 | pos | benzene-1,2,4-triol | HMDB0124831 | Benzenoids | Phenols | Benzenetriols and derivatives | 0.327595 | 0.001441 |
| 0.68_152.0681m/z | 152.0681 | 0.677467 | pos | N4-Acetylaminobutanal | HMDB0004226 | Organic oxygen compounds | Organooxygen compounds | Carbonyl compounds | 0.269647 | 8.25E-05 |
| 0.92_194.0421m/z | 194.0421 | 0.924967 | pos | Tetrahydrodipicolinate | HMDB0012289 | Organic acids and derivatives | Carboxylic acids and derivatives | Amino acids, peptides, and analogues | 0.069481 | 0.012476 |
| 1.01_146.0923m/z | 146.0923 | 1.011917 | pos | 4-Guanidinobutanoic acid | HMDB0003464 | Organic acids and derivatives | Carboxylic acids and derivatives | Amino acids, peptides, and analogues | 0.511883 | 0.007941 |
| 11.00_179.0559m/z | 179.0559 | 11.00007 | neg | D-Lactic acid | HMDB0001311 | Organic acids and derivatives | Hydroxy acids and derivatives | Alpha hydroxy acids and derivatives | 0.148302 | 0.001852 |
| 11.05_104.1071m/z | 104.1071 | 11.04965 | pos | Prenol | HMDB0030124 | Organic oxygen compounds | Organooxygen compounds | Alcohols and polyols | 0.216653 | 8.41E-06 |
| 12.48_180.9895m/z | 180.9895 | 12.48145 | pos | Dihydroxyacetone Phosphate Acyl Ester | HMDB0011750 | Organic oxygen compounds | Organooxygen compounds | Carbonyl compounds | 0.749903 | 3.97E-05 |
| 13.31_730.5371m/z | 730.5371 | 13.3062 | pos | PC(16:1(9Z)/16:1(9Z)) | HMDB0008002 | Lipids and lipid-like molecules | Glycerophospholipids | Glycerophosphocholines | 3.039855 | 0.000865 |
| 4.67_259.1516m/z | 259.1516 | 4.668483 | pos | Tetrahydrobiopterin | HMDB0000027 | Organoheterocyclic compounds | Pteridines and derivatives | Pterins and derivatives | 0.39479 | 0.009767 |
| 4.89_291.1339m/z | 291.1339 | 4.888733 | pos | L-Thyronine | HMDB0000667 | Organic acids and derivatives | Carboxylic acids and derivatives | Amino acids, peptides, and analogues | 0.117632 | 0.006333 |
| 0.62_275.0565m/z | 275.0565 | 0.6222 | neg | Salicylic acid | HMDB0001895 | Benzenoids | Benzene and substituted derivatives | Benzoic acids and derivatives | 0.393033 | 1.71E-05 |
| 1.05_256.0941m/z | 256.0941 | 1.045383 | neg | N-gamma-Glutamylglutamine | HMDB0029147 | Organic acids and derivatives | Carboxylic acids and derivatives | Amino acids, peptides, and analogues | 0.098536 | 0.036011 |
| 1.12_204.0631m/z | 204.0631 | 1.118033 | pos | L-Tyrosine | HMDB0000158 | Organic acids and derivatives | Carboxylic acids and derivatives | Amino acids, peptides, and analogues | 0.220692 | 0.004336 |
| 10.47_255.2331m/z | 255.2331 | 10.46582 | neg | 1-Pentadecene | HMDB0031082 | Hydrocarbons | Unsaturated hydrocarbons | Unsaturated aliphatic hydrocarbons | 0.266974 | 0.013016 |
| 13.24_180.9896m/z | 180.9896 | 13.23957 | pos | cis,cis-Muconic acid | HMDB0006331 | Lipids and lipid-like molecules | Fatty Acyls | Fatty acids and conjugates | 2.832047 | 6.85E-05 |
| 14.29_170.0962m/z | 170.0962 | 14.28758 | pos | 4-Aminobiphenyl | HMDB0013195 | Benzenoids | Benzene and substituted derivatives | Biphenyls and derivatives | 0.335291 | 0.045821 |
| 14.51_381.3503m/z | 381.3503 | 14.50852 | pos | Episterol | HMDB0006847 | Lipids and lipid-like molecules | Steroids and steroid derivatives | Ergostane steroids | 0.520899 | 0.034847 |
| 4.35_267.1336m/z | 267.1336 | 4.354317 | pos | Phenylalanyl-Threonine | HMDB0029005 | Organic acids and derivatives | Carboxylic acids and derivatives | Amino acids, peptides, and analogues | 0.116288 | 0.013407 |
| 5.26_240.1593m/z | 240.1593 | 5.2613 | pos | 2-Hydroxyibuprofen | HMDB0060920 | Phenylpropanoids and polyketides | Phenylpropanoic acids | Unclassified | 0.144945 | 0.004607 |
| 5.57_196.0977m/z | 196.0977 | 5.5719 | neg | N-Methyltyramine | HMDB0003633 | Benzenoids | Benzene and substituted derivatives | Phenethylamines | 0.214908 | 0.002232 |
| 8.90_146.0578n | 147.065 | 8.903033 | pos | Adipic acid | HMDB0000448 | Lipids and lipid-like molecules | Fatty Acyls | Fatty acids and conjugates | 0.197669 | 0.003134 |
| 1.60_168.0655m/z | 168.0655 | 1.6013 | pos | 3-Methoxyanthranilate | HMDB0060374 | Benzenoids | Benzene and substituted derivatives | Benzoic acids and derivatives | 0.11396 | 0.030638 |
| 11.33_311.1621m/z | 311.1621 | 11.32643 | pos | Estriol | HMDB0000153 | Lipids and lipid-like molecules | Steroids and steroid derivatives | Estrane steroids | 0.395451 | 0.00012 |
| 2.08_235.0823m/z | 235.0823 | 2.077467 | neg | Galactosylglycerol | HMDB0006790 | Lipids and lipid-like molecules | Glycerolipids | Glycosylglycerols | 0.269044 | 0.005276 |
| 5.21_259.0467m/z | 259.0467 | 5.213367 | neg | Mesaconic acid | HMDB0000749 | Lipids and lipid-like molecules | Fatty Acyls | Fatty acids and conjugates | 0.010079 | 0.036795 |
| 5.40_263.0598m/z | 263.0598 | 5.3976 | neg | 5,6-Dihydro-5-fluorouracil | HMDB0060402 | Organoheterocyclic compounds | Diazines | Pyrimidines and pyrimidine derivatives | 0.170506 | 0.039724 |
| 0.68_188.1756m/z | 188.1756 | 0.677467 | pos | N1-Acetylspermidine | HMDB0001276 | Organic acids and derivatives | Carboximidic acids and derivatives | Carboximidic acids | 0.255166 | 0.008999 |
| 10.60_239.0675m/z | 239.0675 | 10.5958 | neg | 4-Aminohippuric acid | HMDB0001867 | Benzenoids | Benzene and substituted derivatives | Benzoic acids and derivatives | 0.256145 | 0.007614 |
| 10.92_179.0558m/z | 179.0558 | 10.92357 | neg | (R)-2,3-Dihydroxy-isovalerate | HMDB0012141 | Lipids and lipid-like molecules | Fatty Acyls | Fatty acids and conjugates | 0.114589 | 0.021205 |
| 2.41_171.0657m/z | 171.0657 | 2.4088 | neg | Ethenyl acetate | HMDB0031209 | Organic acids and derivatives | Carboxylic acids and derivatives | Carboxylic acid derivatives | 0.493681 | 0.030562 |
| 4.25_265.1181m/z | 265.1181 | 4.2506 | pos | Acetyl-N-formyl-5-methoxykynurenamine | HMDB0004259 | Organic oxygen compounds | Organooxygen compounds | Carbonyl compounds | 0.239009 | 0.00059 |
| 4.28_267.0425m/z | 267.0425 | 4.284233 | pos | Resveratrol | HMDB0003747 | Phenylpropanoids and polyketides | Stilbenes | Unclassified | 0.300133 | 0.033779 |
| 4.55_152.1069m/z | 152.1069 | 4.551767 | pos | Phenylpropanolamine | HMDB0001942 | Benzenoids | Benzene and substituted derivatives | Phenylpropanes | 0.051177 | 0.048642 |
| 5.22_201.0224m/z | 201.0224 | 5.2223 | neg | 4-ethylphenylsulfate | HMDB0062551 | Organic acids and derivatives | Organic sulfuric acids and derivatives | Arylsulfates | 0.24137 | 0.036509 |
| 5.37_242.0576m/z | 242.0576 | 5.366633 | pos | 3-Indolebutyric acid | HMDB0002096 | Organoheterocyclic compounds | Indoles and derivatives | Indoles | 0.13945 | 0.022839 |
| 7.64_187.1336m/z | 187.1336 | 7.644567 | neg | 2,6-Dimethyl-4-heptanone | HMDB0031417 | Organic oxygen compounds | Organooxygen compounds | Carbonyl compounds | 0.184134 | 0.024046 |
| 9.68_194.0821m/z | 194.0821 | 9.675733 | neg | 2-Phenyl-1,3-propanediol monocarbamate | HMDB0060351 | Benzenoids | Benzene and substituted derivatives | Unclassified | 0.144525 | 0.010548 |
| 0.68_266.0883m/z | 266.0883 | 0.683917 | neg | N-Acetylgalactosamine | HMDB0000212 | Organic oxygen compounds | Organooxygen compounds | Carbohydrates and carbohydrate conjugates | 0.412572 | 0.004249 |
| 0.70_203.1501m/z | 203.1501 | 0.6975 | pos | Asymmetric dimethylarginine | HMDB0001539 | Organic acids and derivatives | Carboxylic acids and derivatives | Amino acids, peptides, and analogues | 1.155679 | 9.26E-08 |
| 1.55_211.0845n | 212.0917 | 1.553533 | pos | 3-Methoxytyrosine | HMDB0001434 | Organic acids and derivatives | Carboxylic acids and derivatives | Amino acids, peptides, and analogues | 0.265898 | 0.000108 |
| 12.59_163.1478m/z | 163.1478 | 12.58927 | pos | 1,3-Diisopropylbenzene | HMDB0013806 | Benzenoids | Benzene and substituted derivatives | Cumenes | 0.442592 | 0.022651 |
| 2.74_184.0967m/z | 184.0967 | 2.735283 | pos | 3-(2-Hydroxyphenyl)propanoic acid | HMDB0033752 | Phenylpropanoids and polyketides | Phenylpropanoic acids | Unclassified | 0.247142 | 0.008273 |
| 3.91_113.1076m/z | 113.1076 | 3.914167 | pos | N-Acetylputrescine | HMDB0002064 | Organic acids and derivatives | Carboximidic acids and derivatives | Carboximidic acids | 0.156678 | 1.64E-05 |
| 4.24_332.1259m/z | 332.1259 | 4.236733 | neg | Glutamyltryptophan | HMDB0028830 | Organic acids and derivatives | Carboxylic acids and derivatives | Amino acids, peptides, and analogues | 0.173203 | 0.013144 |
| 4.93_244.1193m/z | 244.1193 | 4.92865 | neg | Ecgonine methyl ester | HMDB0006406 | Alkaloids and derivatives | Tropane alkaloids | Unclassified | 0.114157 | 0.045024 |
| 5.41_186.0761m/z | 186.0761 | 5.4097 | pos | Homogentisic acid | HMDB0000130 | Benzenoids | Benzene and substituted derivatives | Phenylacetic acids | 0.170832 | 0.017235 |
| 0.70_268.1024m/z | 268.1024 | 0.6975 | pos | Neuraminic acid | HMDB0000830 | Organic oxygen compounds | Organooxygen compounds | Carbohydrates and carbohydrate conjugates | 0.284274 | 0.003657 |
| 0.70_417.0018m/z | 417.0018 | 0.6975 | pos | 6-Methylthiopurine 5'-monophosphate ribonucleotide | HMDB0060414 | Nucleosides, nucleotides, and analogues | Purine nucleotides | Purine ribonucleotides | 1.415218 | 9.59E-05 |
| 0.79_224.0234m/z | 224.0234 | 0.787467 | neg | S-Carboxymethyl-L-cysteine | HMDB0029415 | Organic acids and derivatives | Carboxylic acids and derivatives | Amino acids, peptides, and analogues | 0.453248 | 0.00451 |
| 1.09_164.0380m/z | 164.038 | 1.088183 | neg | Methionine sulfoxide | HMDB0002005 | Organic acids and derivatives | Carboxylic acids and derivatives | Amino acids, peptides, and analogues | 0.301819 | 0.000651 |
| 13.06_813.6821m/z | 813.6821 | 13.0648 | pos | SM(d18:1/24:1(15Z)) | HMDB0012107 | Lipids and lipid-like molecules | Sphingolipids | Phosphosphingolipids | 20.54985 | 3.91E-09 |
| 3.44_279.1340m/z | 279.134 | 3.444183 | pos | N1-(alpha-D-ribosyl)-5,6-dimethyl-benzimidazole | HMDB0011112 | Nucleosides, nucleotides, and analogues | Benzimidazole ribonucleosides and ribonucleotides | Unclassified | 0.684077 | 1.41E-06 |
| 3.86_311.1389m/z | 311.1389 | 3.856083 | pos | Phenylalanyl-Tyrosine | HMDB0029007 | Organic acids and derivatives | Carboxylic acids and derivatives | Amino acids, peptides, and analogues | 0.099485 | 0.013224 |
| 4.23_263.0782m/z | 263.0782 | 4.226717 | neg | Ethylmalonic acid | HMDB0000622 | Lipids and lipid-like molecules | Fatty Acyls | Fatty acids and conjugates | 0.177824 | 0.005801 |
| 4.83_256.1193m/z | 256.1193 | 4.826567 | neg | Isoproterenol | HMDB0015197 | Benzenoids | Phenols | Benzenediols | 0.196412 | 0.029313 |
| 6.55_365.2340m/z | 365.234 | 6.545033 | neg | Tetrahydrocortisol | HMDB0000949 | Lipids and lipid-like molecules | Steroids and steroid derivatives | Bile acids, alcohols and derivatives | 0.24488 | 0.048857 |
| 8.10_268.1037m/z | 268.1037 | 8.10235 | pos | Zidovudine | HMDB0014638 | Nucleosides, nucleotides, and analogues | Pyrimidine nucleosides | Pyrimidine 2',3'-dideoxyribonucleosides | 0.232293 | 0.000511 |
| 8.30_310.1130m/z | 310.113 | 8.304333 | pos | N-Acetylneuraminic acid | HMDB0000230 | Organic oxygen compounds | Organooxygen compounds | Carbohydrates and carbohydrate conjugates | 0.096898 | 0.033012 |
| 9.50_195.1389m/z | 195.1389 | 9.50145 | neg | Cyclohexanone | HMDB0003315 | Organic oxygen compounds | Organooxygen compounds | Carbonyl compounds | 0.132137 | 0.039846 |
| 0.58_143.0338m/z | 143.0338 | 0.578 | pos | 3-Oxoadipic acid | HMDB0000398 | Organic acids and derivatives | Keto acids and derivatives | Medium-chain keto acids and derivatives | 0.278219 | 0.001609 |
| 0.70_159.0762m/z | 159.0762 | 0.6975 | pos | 2-Aminomuconic acid semialdehyde | HMDB0001280 | Organic acids and derivatives | Carboxylic acids and derivatives | Amino acids, peptides, and analogues | 0.637388 | 0.000359 |
| 0.76_132.1018m/z | 132.1018 | 0.755917 | pos | L-Isoleucine | HMDB0000172 | Organic acids and derivatives | Carboxylic acids and derivatives | Amino acids, peptides, and analogues | 0.328363 | 0.00272 |
| 0.84_134.0447m/z | 134.0447 | 0.839567 | pos | Fumaric acid | HMDB0000134 | Organic acids and derivatives | Carboxylic acids and derivatives | Dicarboxylic acids and derivatives | 0.096531 | 0.008575 |
| 1.06_156.0058n | 139.0025 | 1.055433 | pos | 2,5-Furandicarboxylic acid | HMDB0004812 | Organoheterocyclic compounds | Furans | Furoic acid and derivatives | 0.375625 | 4.57E-08 |
| 1.06_242.1132m/z | 242.1132 | 1.055433 | pos | L-3-Hydroxykynurenine | HMDB0011631 | Organic oxygen compounds | Organooxygen compounds | Carbonyl compounds | 0.205937 | 0.04306 |
| 1.06_282.1194m/z | 282.1194 | 1.055433 | pos | N6-Methyladenosine | HMDB0004044 | Nucleosides, nucleotides, and analogues | Purine nucleosides | Unclassified | 0.272133 | 0.000596 |
| 10.58_281.2492m/z | 281.2492 | 10.58202 | neg | Oleic acid | HMDB0000207 | Lipids and lipid-like molecules | Fatty Acyls | Fatty acids and conjugates | 0.192327 | 0.041727 |
| 11.86_554.3838m/z | 554.3838 | 11.86475 | neg | LysoPC(O-18:0) | HMDB0011149 | Lipids and lipid-like molecules | Glycerophospholipids | Glycerophosphocholines | 0.789993 | 0.01 |
| 13.39_137.0000m/z | 137 | 13.39332 | pos | 2-Furanmethanol | HMDB0013742 | Organoheterocyclic compounds | Heteroaromatic compounds | Unclassified | 0.32507 | 5.48E-05 |
| 3.95_215.0848m/z | 215.0848 | 3.9481 | pos | Methyl bisnorbiotinyl ketone | HMDB0004822 | Organoheterocyclic compounds | Thienoimidazolidines | Unclassified | 0.189637 | 0.033507 |
| 4.08_193.0504m/z | 193.0504 | 4.07695 | neg | 1-Phenyl-1,2-propanedione | HMDB0035243 | Benzenoids | Benzene and substituted derivatives | Phenylpropanes | 0.147473 | 0.001507 |
| 5.84_363.2184m/z | 363.2184 | 5.840933 | neg | 3a,11b,21-Trihydroxy-20-oxo-5b-pregnan-18-al | HMDB0006753 | Lipids and lipid-like molecules | Steroids and steroid derivatives | Hydroxysteroids | 0.29067 | 0.000184 |
| 7.30_386.1993m/z | 386.1993 | 7.301733 | pos | Dehydroepiandrosterone sulfate | HMDB0001032 | Lipids and lipid-like molecules | Steroids and steroid derivatives | Sulfated steroids | 1.147575 | 0.015051 |
| 8.99_177.0545m/z | 177.0545 | 8.991583 | pos | trans-Ferulic acid | HMDB0000954 | Phenylpropanoids and polyketides | Cinnamic acids and derivatives | Hydroxycinnamic acids and derivatives | 0.097071 | 0.045978 |
| 9.10_345.2077m/z | 345.2077 | 9.09605 | neg | all-trans-Retinoic acid | HMDB0001852 | Lipids and lipid-like molecules | Prenol lipids | Retinoids | 0.376339 | 0.004644 |
| 0.86_276.1189m/z | 276.1189 | 0.861417 | pos | Ribothymidine | HMDB0000884 | Nucleosides, nucleotides, and analogues | Pyrimidine nucleosides | Unclassified | 0.276928 | 0.002955 |
| 0.99_133.0972m/z | 133.0972 | 0.9902 | pos | D-Proline | HMDB0003411 | Organic acids and derivatives | Carboxylic acids and derivatives | Amino acids, peptides, and analogues | 0.144191 | 0.000355 |
| 1.36_128.0455m/z | 128.0455 | 1.361683 | pos | 1-Methyl-4-nitroimidazole | HMDB0060659 | Organoheterocyclic compounds | Azoles | Imidazoles | 0.047515 | 0.007528 |
| 11.01_454.3884m/z | 454.3884 | 11.00617 | pos | 27-Deoxy-5b-cyprinol | HMDB0001231 | Lipids and lipid-like molecules | Steroids and steroid derivatives | Bile acids, alcohols and derivatives | 0.77688 | 0.000935 |
| 12.29_768.5873m/z | 768.5873 | 12.28563 | pos | PC(18:1(11Z)/18:1(9Z)) | HMDB0008071 | Lipids and lipid-like molecules | Glycerophospholipids | Glycerophosphocholines | 1.52504 | 0.033221 |
| 14.83_198.0170m/z | 198.017 | 14.83202 | pos | O-Phospho-4-hydroxy-L-threonine | HMDB0006802 | Organic acids and derivatives | Carboxylic acids and derivatives | Amino acids, peptides, and analogues | 0.636864 | 0.000412 |
| 2.17_181.0971m/z | 181.0971 | 2.174117 | pos | Tyrosinamide | HMDB0013319 | Organic acids and derivatives | Carboxylic acids and derivatives | Amino acids, peptides, and analogues | 0.101434 | 3.43E-05 |
| 5.51_179.0557m/z | 179.0557 | 5.5096 | neg | Alpha-D-Glucose | HMDB0003345 | Organic oxygen compounds | Organooxygen compounds | Carbohydrates and carbohydrate conjugates | 0.107406 | 0.03542 |
| 7.89_179.0557m/z | 179.0557 | 7.89 | neg | scyllo-Inositol | HMDB0006088 | Organic oxygen compounds | Organooxygen compounds | Alcohols and polyols | 0.219038 | 0.001256 |
| 8.47_347.2236m/z | 347.2236 | 8.469183 | neg | Retinyl ester | HMDB0003598 | Lipids and lipid-like molecules | Prenol lipids | Retinoids | 0.54232 | 0.001261 |
| 0.52_136.0617m/z | 136.0617 | 0.516683 | pos | Adenine | HMDB0000034 | Organoheterocyclic compounds | Imidazopyrimidines | Purines and purine derivatives | 0.275139 | 0.013306 |
| 11.85_133.1009m/z | 133.1009 | 11.84975 | pos | (+)-(S)-Carvone | HMDB0004487 | Lipids and lipid-like molecules | Prenol lipids | Monoterpenoids | 0.24807 | 0.000634 |
| 2.07_186.0760m/z | 186.076 | 2.07395 | pos | p-Hydroxymandelic acid | HMDB0000822 | Benzenoids | Phenols | 1-hydroxy-2-unsubstituted benzenoids | 0.109835 | 0.047833 |
| 4.73_545.0583m/z | 545.0583 | 4.732617 | neg | dTDP-D-glucose | HMDB0001328 | Nucleosides, nucleotides, and analogues | Pyrimidine nucleotides | Pyrimidine nucleotide sugars | 0.36034 | 3.25E-05 |
| 4.93_186.0760m/z | 186.076 | 4.933117 | pos | 3-Hydroxymandelic acid | HMDB0000750 | Benzenoids | Phenols | 1-hydroxy-4-unsubstituted benzenoids | 0.135833 | 0.032406 |
| 7.81_143.0107m/z | 143.0107 | 7.814033 | pos | 2-Hydroxybutyric acid | HMDB0000008 | Organic acids and derivatives | Hydroxy acids and derivatives | Alpha hydroxy acids and derivatives | 0.078357 | 0.001505 |
| 8.05_130.0862m/z | 130.0862 | 8.05135 | pos | 2-Pyrrolidineacetic acid | HMDB0029444 | Organoheterocyclic compounds | Pyrrolidines | Unclassified | 0.20793 | 0.001159 |
| 9.71_136.0756m/z | 136.0756 | 9.71215 | pos | p-Octopamine | HMDB0004825 | Benzenoids | Phenols | 1-hydroxy-2-unsubstituted benzenoids | 0.090664 | 0.028114 |
| 0.76_420.9702m/z | 420.9702 | 0.755917 | pos | Inositol 1,3,4-trisphosphate | HMDB0001143 | Organic oxygen compounds | Organooxygen compounds | Alcohols and polyols | 0.319704 | 0.029661 |
| 1.06_129.1022m/z | 129.1022 | 1.055433 | pos | (3S,5S)-3,5-Diaminohexanoate | HMDB0012115 | Organic acids and derivatives | Carboxylic acids and derivatives | Amino acids, peptides, and analogues | 0.148418 | 0.001266 |
| 1.14_148.0967m/z | 148.0967 | 1.139867 | pos | Adipate semialdehyde | HMDB0012882 | Lipids and lipid-like molecules | Fatty Acyls | Fatty acids and conjugates | 0.090972 | 0.004446 |
| 11.25_193.0062m/z | 193.0062 | 11.24683 | pos | Diethylthiophosphate | HMDB0001460 | Organic acids and derivatives | Organic thiophosphoric acids and derivatives | Thiophosphoric acid esters | 0.230943 | 4.76E-05 |
| 11.90_445.3331m/z | 445.3331 | 11.90243 | neg | 7a-Hydroxy-cholestene-3-one | HMDB0001993 | Lipids and lipid-like molecules | Steroids and steroid derivatives | Cholestane steroids | 0.55579 | 0.00124 |
| 13.89_768.5529m/z | 768.5529 | 13.89148 | pos | PC(20:4(5Z,8Z,11Z,14Z)/15:0) | HMDB0008428 | Lipids and lipid-like molecules | Glycerophospholipids | Glycerophosphocholines | 0.904978 | 0.043827 |
| 0.52_152.0566m/z | 152.0566 | 0.516683 | pos | Guanine | HMDB0000132 | Organoheterocyclic compounds | Imidazopyrimidines | Purines and purine derivatives | 0.165376 | 0.002163 |
| 0.78_174.0873m/z | 174.0873 | 0.77695 | pos | Imidazolelactic acid | HMDB0002320 | Organoheterocyclic compounds | Azoles | Imidazoles | 0.189817 | 0.003292 |
| 1.25_115.0392m/z | 115.0392 | 1.251383 | pos | Dimethylmalonic acid | HMDB0002001 | Organic acids and derivatives | Carboxylic acids and derivatives | Dicarboxylic acids and derivatives | 0.154582 | 7.33E-05 |
| 1.34_183.0208n | 184.0281 | 1.338683 | pos | L-Homocysteic acid | HMDB0002205 | Organic acids and derivatives | Carboxylic acids and derivatives | Amino acids, peptides, and analogues | 0.295237 | 0.00074 |
| 1.56_153.0406m/z | 153.0406 | 1.562867 | pos | Xanthine | HMDB0000292 | Organoheterocyclic compounds | Imidazopyrimidines | Purines and purine derivatives | 0.1193 | 0.040698 |
| 11.30_327.2549m/z | 327.2549 | 11.3036 | neg | Vaccenic acid | HMDB0003231 | Lipids and lipid-like molecules | Fatty Acyls | Fatty acids and conjugates | 0.224553 | 0.026006 |
| 11.30_447.3488m/z | 447.3488 | 11.3036 | neg | 24-Hydroxycholesterol | HMDB0001419 | Lipids and lipid-like molecules | Steroids and steroid derivatives | Bile acids, alcohols and derivatives | 0.437441 | 0.001929 |
| 11.52_1074.6905m/z | 1074.69 | 11.52013 | pos | Trihexosylceramide (d18:1/18:0) | HMDB0004880 | Lipids and lipid-like molecules | Sphingolipids | Glycosphingolipids | 2.764643 | 7.14E-08 |
| 2.98_382.1012m/z | 382.1012 | 2.975 | neg | Succinyladenosine | HMDB0000912 | Nucleosides, nucleotides, and analogues | Purine nucleosides | Unclassified | 0.373525 | 0.000675 |
| 3.50_158.0812m/z | 158.0812 | 3.4983 | pos | 2-methoxybenzene-1,3-diol | HMDB0133970 | Benzenoids | Phenols | Methoxyphenols | 0.108367 | 0.021457 |
| 0.54_887.5632m/z | 887.5632 | 0.537917 | pos | PI(18:1(9Z)/20:3(8Z,11Z,14Z)) | HMDB0009843 | Lipids and lipid-like molecules | Glycerophospholipids | Glycerophosphoinositols | 1.289809 | 0.000403 |
| 0.70_209.0300m/z | 209.03 | 0.703767 | neg | Glucaric acid | HMDB0000663 | Organic oxygen compounds | Organooxygen compounds | Carbohydrates and carbohydrate conjugates | 0.417066 | 0.031271 |
| 0.80_148.0967m/z | 148.0967 | 0.796917 | pos | 2-Methyl-3-ketovaleric acid | HMDB0000408 | Organic acids and derivatives | Keto acids and derivatives | Short-chain keto acids and derivatives | 0.273499 | 1.13E-05 |
| 0.88_106.0502m/z | 106.0502 | 0.882533 | pos | L-Serine | HMDB0000187 | Organic acids and derivatives | Carboxylic acids and derivatives | Amino acids, peptides, and analogues | 0.168755 | 0.00013 |
| 1.70_241.0036n | 242.0109 | 1.70135 | pos | Indole-3-carboxilic acid-O-sulphate | HMDB0060002 | Organoheterocyclic compounds | Indoles and derivatives | Indolecarboxylic acids and derivatives | 0.437957 | 0.026071 |
| 2.09_244.9983m/z | 244.9983 | 2.08795 | neg | Dimethylallylpyrophosphate | HMDB0001120 | Lipids and lipid-like molecules | Prenol lipids | Isoprenoid phosphates | 0.127499 | 0.023934 |
| 2.23_113.0236m/z | 113.0236 | 2.228183 | pos | 2-Furoic acid | HMDB0000617 | Organoheterocyclic compounds | Furans | Furoic acid and derivatives | 0.223962 | 2.15E-05 |
| 4.11_158.0816m/z | 158.0816 | 4.10695 | neg | N-Acetylvaline | HMDB0011757 | Organic acids and derivatives | Carboxylic acids and derivatives | Amino acids, peptides, and analogues | 0.105046 | 0.030051 |
| 7.77_166.0862m/z | 166.0862 | 7.7738 | pos | trans-Cinnamic acid | HMDB0000930 | Phenylpropanoids and polyketides | Cinnamic acids and derivatives | Cinnamic acids | 0.071275 | 0.037927 |
| 8.90_119.0492m/z | 119.0492 | 8.903033 | pos | 2-Methylbenzoic acid | HMDB0002340 | Benzenoids | Benzene and substituted derivatives | Benzoic acids and derivatives | 0.123152 | 0.007707 |
| 0.73_146.0445m/z | 146.0445 | 0.734083 | pos | 2-Keto-glutaramic acid | HMDB0001552 | Organic acids and derivatives | Keto acids and derivatives | Short-chain keto acids and derivatives | 0.179752 | 0.015487 |
| 0.73_189.1230m/z | 189.123 | 0.734083 | pos | Glycylleucine | HMDB0000759 | Organic acids and derivatives | Carboxylic acids and derivatives | Amino acids, peptides, and analogues | 0.299616 | 0.042759 |
| 0.73_259.0921m/z | 259.0921 | 0.734083 | pos | 3-Methyluridine | HMDB0004813 | Nucleosides, nucleotides, and analogues | Pyrimidine nucleosides | Unclassified | 0.434065 | 0.017054 |
| 0.94_327.1303m/z | 327.1303 | 0.937417 | neg | L-Rhamnulose | HMDB0010207 | Lipids and lipid-like molecules | Fatty Acyls | Fatty alcohols | 0.1995 | 0.016291 |
| 0.97_138.0913m/z | 138.0913 | 0.968517 | pos | 3-Methylbenzaldehyde | HMDB0029637 | Benzenoids | Benzene and substituted derivatives | Benzoyl derivatives | 0.079933 | 0.01163 |
| 1.34_293.0543m/z | 293.0543 | 1.338683 | pos | p-Hydroxyfelbamate | HMDB0060669 | Benzenoids | Phenols | 1-hydroxy-2-unsubstituted benzenoids | 0.339015 | 0.001835 |
| 1.69_244.9756m/z | 244.9756 | 1.6933 | neg | Methylgallic acid-O-sulphate | HMDB0060005 | Benzenoids | Benzene and substituted derivatives | Benzoic acids and derivatives | 0.256067 | 0.04485 |
| 12.21_742.5404m/z | 742.5404 | 12.20688 | neg | PC(15:0/18:2(9Z,12Z)) | HMDB0007940 | Lipids and lipid-like molecules | Glycerophospholipids | Glycerophosphocholines | 1.317557 | 0.039834 |
| 13.87_856.5809m/z | 856.5809 | 13.87005 | pos | PC(18:1(9Z)/22:5(4Z,7Z,10Z,13Z,16Z)) | HMDB0008121 | Lipids and lipid-like molecules | Glycerophospholipids | Glycerophosphocholines | 1.576192 | 0.038299 |
| 3.70_113.0599m/z | 113.0599 | 3.700933 | pos | 2-Ketohexanoic acid | HMDB0001864 | Organic acids and derivatives | Keto acids and derivatives | Medium-chain keto acids and derivatives | 0.114364 | 0.016182 |
| 3.74_155.0449m/z | 155.0449 | 3.741867 | pos | Hydantoin-5-propionic acid | HMDB0001212 | Organoheterocyclic compounds | Azolidines | Imidazolidines | 0.447828 | 0.015925 |
| 7.63_208.1332m/z | 208.1332 | 7.626667 | pos | Prenyl benzoate | HMDB0032488 | Benzenoids | Benzene and substituted derivatives | Benzoic acids and derivatives | 0.08337 | 0.015022 |
| 1.14_221.0665m/z | 221.0665 | 1.136467 | neg | 3-Isopropylmalate | HMDB0012156 | Lipids and lipid-like molecules | Fatty Acyls | Fatty acids and conjugates | 0.106627 | 0.012615 |
| 1.23_244.9981m/z | 244.9981 | 1.23085 | neg | Isopentenyl pyrophosphate | HMDB0001347 | Lipids and lipid-like molecules | Prenol lipids | Isoprenoid phosphates | 0.268704 | 0.000134 |
| 1.57_181.0362m/z | 181.0362 | 1.567817 | neg | 7-Methyluric acid | HMDB0011107 | Organoheterocyclic compounds | Imidazopyrimidines | Purines and purine derivatives | 0.153787 | 0.011678 |
| 10.27_432.3129m/z | 432.3129 | 10.26535 | neg | Lithocholic acid glycine conjugate | HMDB0000698 | Lipids and lipid-like molecules | Steroids and steroid derivatives | Bile acids, alcohols and derivatives | 0.250618 | 0.032199 |
| 2.76_149.0449m/z | 149.0449 | 2.761533 | pos | L-Xylonate | HMDB0060256 | Organic oxygen compounds | Organooxygen compounds | Carbohydrates and carbohydrate conjugates | 0.050379 | 0.009065 |
| 3.58_367.1413m/z | 367.1413 | 3.580183 | neg | Phosphorylcholine | HMDB0001565 | Organic nitrogen compounds | Organonitrogen compounds | Quaternary ammonium salts | 0.10384 | 0.033176 |
| 4.10_418.1341m/z | 418.1341 | 4.096417 | pos | 5-(3',4',5'-Trihydroxyphenyl)-gamma-valerolactone-3'-O-glucuronide | HMDB0059984 | Organic oxygen compounds | Organooxygen compounds | Carbohydrates and carbohydrate conjugates | 0.386632 | 5.53E-05 |
| 4.70_155.1178m/z | 155.1178 | 4.6977 | pos | Tyramine | HMDB0000306 | Benzenoids | Benzene and substituted derivatives | Phenethylamines | 0.044309 | 0.005192 |
| 4.86_162.0913m/z | 162.0913 | 4.8569 | pos | 1-Naphthol | HMDB0012138 | Benzenoids | Naphthalenes | Naphthols and derivatives | 0.096929 | 0.001261 |
| 6.54_136.0756m/z | 136.0756 | 6.541783 | pos | Vanillylamine | HMDB0012309 | Benzenoids | Phenols | Methoxyphenols | 0.369935 | 1.13E-06 |
| 0.66_192.0797m/z | 192.0797 | 0.6568 | pos | 2-Hydroxyiminostilbene | HMDB0060652 | Organoheterocyclic compounds | Benzazepines | Dibenzazepines | 0.332302 | 4.39E-06 |
| 0.68_189.1345m/z | 189.1345 | 0.677467 | pos | Homo-L-arginine | HMDB0000670 | Organic acids and derivatives | Carboxylic acids and derivatives | Amino acids, peptides, and analogues | 0.432978 | 1.18E-05 |
| 1.70_184.0048n | 167.0015 | 1.70135 | pos | 6-thiouric acid | HMDB0060601 | Organoheterocyclic compounds | Imidazopyrimidines | Purines and purine derivatives | 0.132942 | 0.033611 |
| 3.16_182.0812m/z | 182.0812 | 3.157683 | pos | 2-Hydroxycinnamic acid | HMDB0002641 | Phenylpropanoids and polyketides | Cinnamic acids and derivatives | Hydroxycinnamic acids and derivatives | 0.090397 | 0.021908 |
| 3.95_204.0866m/z | 204.0866 | 3.9481 | pos | beta-N-Acetylglucosamine | HMDB0000803 | Organic oxygen compounds | Organooxygen compounds | Carbohydrates and carbohydrate conjugates | 0.164075 | 0.007606 |
| 4.83_335.1717m/z | 335.1717 | 4.826567 | neg | Pyridoxamine | HMDB0001431 | Organoheterocyclic compounds | Pyridines and derivatives | Pyridoxamines | 0.280097 | 0.034457 |
| 6.07_195.0877m/z | 195.0877 | 6.070783 | pos | Caffeine | HMDB0001847 | Organoheterocyclic compounds | Imidazopyrimidines | Purines and purine derivatives | 0.089309 | 0.009801 |
| 6.96_182.0812m/z | 182.0812 | 6.95545 | pos | Enol-phenylpyruvate | HMDB0012225 | Benzenoids | Benzene and substituted derivatives | Phenylpyruvic acid derivatives | 0.091628 | 0.013454 |
| 1.06_201.0869m/z | 201.0869 | 1.055433 | pos | gamma-Glutamylalanine | HMDB0006248 | Organic acids and derivatives | Carboxylic acids and derivatives | Amino acids, peptides, and analogues | 0.127654 | 0.022298 |
| 13.34_481.3333m/z | 481.3333 | 13.3374 | neg | Demethylphylloquinone | HMDB0004649 | Lipids and lipid-like molecules | Prenol lipids | Quinone and hydroquinone lipids | 0.628443 | 0.000387 |
| 13.96_607.3899m/z | 607.3899 | 13.95712 | pos | Zeaxanthin | HMDB0002789 | Lipids and lipid-like molecules | Prenol lipids | Tetraterpenoids | 0.468762 | 0.004809 |
| 3.31_198.0761m/z | 198.0761 | 3.314017 | pos | DL-Dopa | HMDB0000609 | Organic acids and derivatives | Carboxylic acids and derivatives | Amino acids, peptides, and analogues | 0.082836 | 0.012644 |
| 4.85_147.0443m/z | 147.0443 | 4.849867 | neg | D-Phenyllactic acid | HMDB0000563 | Phenylpropanoids and polyketides | Phenylpropanoic acids | Unclassified | 0.094908 | 0.001122 |
| 5.13_322.1859m/z | 322.1859 | 5.13045 | pos | Arginyl-Phenylalanine | HMDB0028716 | Organic acids and derivatives | Carboxylic acids and derivatives | Amino acids, peptides, and analogues | 0.608341 | 0.004605 |
| 1.33_231.0455m/z | 231.0455 | 1.3319 | neg | Glutamylcysteine | HMDB0028816 | Organic acids and derivatives | Carboxylic acids and derivatives | Amino acids, peptides, and analogues | 0.364093 | 0.000489 |
| 1.34_207.0441m/z | 207.0441 | 1.338683 | pos | Lanthionine ketimine | HMDB0004823 | Organic acids and derivatives | Carboxylic acids and derivatives | Amino acids, peptides, and analogues | 0.152984 | 0.002179 |
| 14.02_155.1177m/z | 155.1177 | 14.0231 | pos | 4-Hydroxy-2,6-dimethylaniline | HMDB0060681 | Benzenoids | Phenols | Cresols | 0.092708 | 0.023036 |
| 14.29_135.1167m/z | 135.1167 | 14.28758 | pos | p-Cymene | HMDB0005805 | Lipids and lipid-like molecules | Prenol lipids | Monoterpenoids | 0.241104 | 0.034372 |
| 3.61_472.1593m/z | 472.1593 | 3.613 | neg | 10-Formyltetrahydrofolate | HMDB0000972 | Organoheterocyclic compounds | Dithiolanes | Lipoamides | 0.28749 | 0.001559 |
| 4.04_258.9922m/z | 258.9922 | 4.038483 | neg | Caffeic acid 3-sulfate | HMDB0041706 | Phenylpropanoids and polyketides | Cinnamic acids and derivatives | Hydroxycinnamic acids and derivatives | 0.162244 | 0.000258 |
| 4.33_227.0848m/z | 227.0848 | 4.330583 | pos | Biotin | HMDB0000030 | Organoheterocyclic compounds | Biotin and derivatives | Unclassified | 0.162341 | 0.002472 |
| 4.64_215.1390m/z | 215.139 | 4.636417 | pos | Dethiobiotin | HMDB0003581 | Lipids and lipid-like molecules | Fatty Acyls | Fatty acids and conjugates | 0.246416 | 0.000125 |
| 5.30_194.0457m/z | 194.0457 | 5.301883 | neg | 5,6-Dihydroxyindole | HMDB0004058 | Organoheterocyclic compounds | Indoles and derivatives | Hydroxyindoles | 0.061462 | 0.039593 |
| 8.30_349.2393m/z | 349.2393 | 8.299917 | neg | Tetrahydrocorticosterone | HMDB0000268 | Lipids and lipid-like molecules | Steroids and steroid derivatives | Hydroxysteroids | 0.257934 | 0.00049 |
| 8.60_231.1127m/z | 231.1127 | 8.60425 | pos | 6-Hydroxymelatonin | HMDB0004081 | Organoheterocyclic compounds | Indoles and derivatives | Hydroxyindoles | 0.110151 | 0.028634 |
| 1.18_215.1389m/z | 215.1389 | 1.18395 | pos | Metanephrine | HMDB0004063 | Benzenoids | Phenols | Methoxyphenols | 0.152459 | 0.001486 |
| 10.83_599.5025m/z | 599.5025 | 10.82853 | pos | DG(16:0/20:4(5Z,8Z,11Z,14Z)/0:0) | HMDB0007112 | Lipids and lipid-like molecules | Glycerolipids | Diradylglycerols | 0.536541 | 0.004218 |
| 13.04_743.5460n | 761.5862 | 13.04303 | pos | PE(14:0/22:2(13Z,16Z)) | HMDB0008843 | Lipids and lipid-like molecules | Glycerophospholipids | Glycerophosphoethanolamines | 12.26009 | 0.023122 |
| 3.30_197.0808m/z | 197.0808 | 3.30475 | pos | Homoveratric acid | HMDB0000434 | Benzenoids | Benzene and substituted derivatives | Methoxybenzenes | 0.089669 | 0.010327 |
| 3.57_195.1129m/z | 195.1129 | 3.5682 | pos | 6-Hydroxypseudooxynicotine | HMDB0240264 | Organic oxygen compounds | Organooxygen compounds | Carbonyl compounds | 0.089428 | 0.007571 |
| 0.54_196.0166m/z | 196.0166 | 0.537917 | pos | 1-nitrosonaphthalene | HMDB0062189 | Benzenoids | Naphthalenes | Unclassified | 1.900334 | 4.77E-05 |
| 0.70_120.0656m/z | 120.0656 | 0.6975 | pos | 2-Ketobutyric acid | HMDB0000005 | Organic acids and derivatives | Keto acids and derivatives | Short-chain keto acids and derivatives | 0.834193 | 8.13E-06 |
| 0.74_173.0925m/z | 173.0925 | 0.743133 | neg | Valyl-Glycine | HMDB0029127 | Organic acids and derivatives | Carboxylic acids and derivatives | Amino acids, peptides, and analogues | 0.211208 | 0.025438 |
| 0.83_558.0045m/z | 558.0045 | 0.830067 | neg | 2,5-Diamino-6-(5'-triphosphoryl-3',4'-trihydroxy-2'-oxopentyl)-amino-4-oxopyrimidine | HMDB0006823 | Organic oxygen compounds | Organooxygen compounds | Carbohydrates and carbohydrate conjugates | 1.159897 | 1.94E-08 |
| 0.88_172.0401m/z | 172.0401 | 0.882533 | pos | L-Methionine | HMDB0000696 | Organic acids and derivatives | Carboxylic acids and derivatives | Amino acids, peptides, and analogues | 0.112127 | 0.000451 |
| 1.06_129.0183m/z | 129.0183 | 1.055433 | pos | Oxoglutaric acid | HMDB0000208 | Organic acids and derivatives | Keto acids and derivatives | Gamma-keto acids and derivatives | 0.390851 | 6.74E-06 |
| 1.47_190.0862m/z | 190.0862 | 1.46965 | pos | Menadione | HMDB0001892 | Benzenoids | Naphthalenes | Naphthoquinones | 0.056373 | 0.044505 |
| 10.50_637.2672m/z | 637.2672 | 10.50213 | pos | Coproporphyrin III | HMDB0000570 | Organoheterocyclic compounds | Tetrapyrroles and derivatives | Porphyrins | 0.352418 | 0.000228 |
| 11.43_183.1491m/z | 183.1491 | 11.43337 | pos | Hordenine | HMDB0004366 | Benzenoids | Benzene and substituted derivatives | Phenethylamines | 0.059843 | 0.001117 |
| 6.66_213.0841m/z | 213.0841 | 6.663783 | pos | Diaminopimelic acid | HMDB0001370 | Organic acids and derivatives | Carboxylic acids and derivatives | Amino acids, peptides, and analogues | 0.131239 | 0.001493 |
| 0.68_173.1037m/z | 173.1037 | 0.683917 | neg | D-Arginine | HMDB0003416 | Organic acids and derivatives | Carboxylic acids and derivatives | Amino acids, peptides, and analogues | 0.234502 | 0.0046 |
| 0.73_249.1078m/z | 249.1078 | 0.734083 | pos | N2-Succinyl-L-glutamic acid 5-semialdehyde | HMDB0001180 | Organic acids and derivatives | Carboxylic acids and derivatives | Amino acids, peptides, and analogues | 0.622403 | 2.53E-06 |
| 0.92_242.0787m/z | 242.0787 | 0.915283 | neg | N-Acetylhistidine | HMDB0032055 | Organic acids and derivatives | Carboxylic acids and derivatives | Amino acids, peptides, and analogues | 0.130273 | 0.02174 |
| 1.05_166.0140m/z | 166.014 | 1.045383 | neg | Quinolinic acid | HMDB0000232 | Organoheterocyclic compounds | Pyridines and derivatives | Pyridinecarboxylic acids and derivatives | 0.102023 | 0.009476 |
| 1.12_102.0552m/z | 102.0552 | 1.118033 | pos | L-Threonine | HMDB0000167 | Organic acids and derivatives | Carboxylic acids and derivatives | Amino acids, peptides, and analogues | 0.147859 | 0.000444 |
| 10.94_599.5027m/z | 599.5027 | 10.93927 | pos | DG(18:2(9Z,12Z)/18:2(9Z,12Z)/0:0) | HMDB0007248 | Lipids and lipid-like molecules | Fatty Acyls | Lineolic acids and derivatives | 0.642878 | 0.001013 |
| 11.45_433.3785m/z | 433.3785 | 11.45482 | pos | Squalene | HMDB0000256 | Lipids and lipid-like molecules | Prenol lipids | Triterpenoids | 0.79939 | 7.3E-06 |
| 12.53_490.1675m/z | 490.1675 | 12.52952 | neg | Tetrahydrofolic acid | HMDB0001846 | Organoheterocyclic compounds | Imidazopyrimidines | Purines and purine derivatives | 0.222664 | 0.038296 |
| 4.68_105.0372m/z | 105.0372 | 4.677933 | pos | 3-(Methylthio)propanal | HMDB0031857 | Organic oxygen compounds | Organooxygen compounds | Carbonyl compounds | 0.086811 | 0.030487 |
| 1.21_212.0917m/z | 212.0917 | 1.2065 | pos | Methyldopa | HMDB0011754 | Phenylpropanoids and polyketides | Phenylpropanoic acids | Unclassified | 0.085203 | 0.003096 |
| 1.53_219.0775m/z | 219.0775 | 1.52885 | neg | Felbamate | HMDB0015084 | Benzenoids | Benzene and substituted derivatives | Unclassified | 0.169855 | 0.005255 |
| 13.76_756.5524m/z | 756.5524 | 13.76133 | pos | PC(14:1(9Z)/20:2(11Z,14Z)) | HMDB0007913 | Lipids and lipid-like molecules | Glycerophospholipids | Glycerophosphocholines | 3.86073 | 0.000659 |
| 3.69_163.0394m/z | 163.0394 | 3.6894 | neg | 3-Methoxy-4-hydroxyphenylglycolaldehyde | HMDB0004061 | Benzenoids | Phenols | Methoxyphenols | 0.115611 | 0.005224 |
| 3.99_165.0188m/z | 165.0188 | 3.98965 | neg | Terephthalic acid | HMDB0002428 | Benzenoids | Benzene and substituted derivatives | Benzoic acids and derivatives | 0.047908 | 0.041345 |
| 9.28_482.2956m/z | 482.2956 | 9.2759 | neg | Lithocholyltaurine | HMDB0000722 | Lipids and lipid-like molecules | Steroids and steroid derivatives | Bile acids, alcohols and derivatives | 0.692815 | 0.000126 |
| 13.76_269.1619m/z | 269.1619 | 13.76133 | pos | Histidinyl-Isoleucine | HMDB0028888 | Organic acids and derivatives | Carboxylic acids and derivatives | Amino acids, peptides, and analogues | 0.452247 | 0.000115 |
| 4.24_270.1912m/z | 270.1912 | 4.235433 | pos | Arginyl-Isoleucine | HMDB0028712 | Organic acids and derivatives | Carboxylic acids and derivatives | Amino acids, peptides, and analogues | 0.117356 | 0.036203 |
| 1.64_231.0455m/z | 231.0455 | 1.641483 | neg | gamma-Glutamylcysteine | HMDB0001049 | Organic acids and derivatives | Carboxylic acids and derivatives | Amino acids, peptides, and analogues | 0.259834 | 0.000928 |
| 14.83_201.9755m/z | 201.9755 | 14.83202 | pos | 3-Methylsulfinylpropyl isothiocyanate | HMDB0006095 | Organosulfur compounds | Sulfoxides | Unclassified | 1.244353 | 0.00617 |
| 11.74_796.5239m/z | 796.5239 | 11.73907 | pos | PC(16:1(9Z)/18:1(9Z)) | HMDB0008005 | Lipids and lipid-like molecules | Glycerophospholipids | Glycerophosphocholines | 1.303004 | 0.006207 |
| 13.89_766.5717m/z | 766.5717 | 13.89148 | pos | PC(18:1(9Z)/P-16:0) | HMDB0008126 | Lipids and lipid-like molecules | Glycerophospholipids | Glycerophosphocholines | 1.229975 | 0.047627 |
| 4.28_230.0074m/z | 230.0074 | 4.280083 | neg | Phosphoserine | HMDB0000272 | Organic acids and derivatives | Carboxylic acids and derivatives | Amino acids, peptides, and analogues | 0.071802 | 0.02493 |
| 4.53_331.1515m/z | 331.1515 | 4.53305 | pos | Tyramine glucuronide | HMDB0010328 | Organic oxygen compounds | Organooxygen compounds | Carbohydrates and carbohydrate conjugates | 0.13742 | 0.00248 |
| 4.83_233.1284m/z | 233.1284 | 4.831567 | pos | Melatonin | HMDB0001389 | Organoheterocyclic compounds | Indoles and derivatives | Indoles | 0.125005 | 0.001474 |
| 13.11_811.6635m/z | 811.6635 | 13.10807 | pos | SM(d18:0/22:0) | HMDB0012091 | Lipids and lipid-like molecules | Sphingolipids | Phosphosphingolipids | 6.766103 | 0.003111 |
| 3.50_216.9805m/z | 216.9805 | 3.501517 | neg | 4-hydroxybenzoic acid-4-O-sulphate | HMDB0059982 | Organic acids and derivatives | Organic sulfuric acids and derivatives | Arylsulfates | 0.332033 | 3.66E-06 |
| 12.22_874.6666m/z | 874.6666 | 12.21733 | neg | PC(22:2(13Z,16Z)/22:2(13Z,16Z)) | HMDB0008612 | Lipids and lipid-like molecules | Glycerophospholipids | Glycerophosphocholines | 1.467693 | 0.000132 |
| 1.80_143.9967m/z | 143.9967 | 1.7971 | pos | Dimethyl trisulfide | HMDB0013780 | Organosulfur compounds | Organic trisulfides | Unclassified | 1.638508 | 3.38E-08 |
| 10.50_220.1178m/z | 220.1178 | 10.50213 | pos | 4-ethylamino-6-isopropylamino-1,3,5-triazin-2-ol | HMDB0062766 | Organoheterocyclic compounds | Triazines | 1,3,5-triazines | 0.100184 | 0.009264 |
| 4.85_235.0885m/z | 235.0885 | 4.849867 | neg | Carbamazepine | HMDB0014704 | Organoheterocyclic compounds | Benzazepines | Dibenzazepines | 0.183287 | 0.006567 |
| 11.01_571.2905m/z | 571.2905 | 11.01452 | neg | D-Urobilinogen | HMDB0004158 | Organoheterocyclic compounds | Tetrapyrroles and derivatives | Bilirubins | 1.038605 | 0.033578 |
| 12.18_856.5803m/z | 856.5803 | 12.17563 | pos | PC(20:3(8Z,11Z,14Z)/20:3(8Z,11Z,14Z)) | HMDB0008409 | Lipids and lipid-like molecules | Glycerophospholipids | Glycerophosphocholines | 0.69208 | 0.032084 |
| 13.20_785.6504m/z | 785.6504 | 13.19578 | pos | SM(d18:1/22:1(13Z)) | HMDB0012104 | Lipids and lipid-like molecules | Sphingolipids | Phosphosphingolipids | 2.473252 | 0.000469 |
| 1.58_130.9664m/z | 130.9664 | 1.580383 | pos | trans-3-Chloro-2-propene-1-ol | HMDB0060514 | Organohalogen compounds | Vinyl halides | Vinyl chlorides | 0.536402 | 0.000884 |
| 10.57_470.3833m/z | 470.3833 | 10.56985 | pos | 5b-Cholestane-3a,7a,12a,23R,25-pentol | HMDB0000513 | Lipids and lipid-like molecules | Fatty Acyls | Fatty acids and conjugates | 0.324765 | 0.006628 |
| 11.72_874.6667m/z | 874.6667 | 11.71908 | neg | PC(24:0/20:4(8Z,11Z,14Z,17Z)) | HMDB0008773 | Lipids and lipid-like molecules | Glycerophospholipids | Glycerophosphocholines | 1.198771 | 0.00017 |
| 13.94_801.6827m/z | 801.6827 | 13.93542 | pos | SM(d18:1/23:0) | HMDB0012105 | Lipids and lipid-like molecules | Sphingolipids | Phosphosphingolipids | 1.229062 | 1.56E-05 |
| 12.66_494.1066m/z | 494.1066 | 12.65677 | neg | Cyanidin 3-glucoside | HMDB0030684 | Organoheterocyclic compounds | Pyrans | Pyranones and derivatives | 0.388277 | 0.000772 |
| 0.62_106.0290m/z | 106.029 | 0.616767 | pos | Nicotinic acid | HMDB0001488 | Organoheterocyclic compounds | Pyridines and derivatives | Pyridinecarboxylic acids and derivatives | 0.106643 | 0.043288 |
| 0.79_283.1036m/z | 283.1036 | 0.787467 | neg | gamma-Glutamylhistidine | HMDB0029151 | Organic acids and derivatives | Carboxylic acids and derivatives | Amino acids, peptides, and analogues | 0.204916 | 0.031561 |
| 14.07_792.5869m/z | 792.5869 | 14.06745 | pos | PC(18:1(9Z)/P-18:1(9Z)) | HMDB0008129 | Lipids and lipid-like molecules | Glycerophospholipids | Glycerophosphocholines | 0.503943 | 0.036729 |
| 4.37_255.1464m/z | 255.1464 | 4.373733 | pos | Histidinyl-Valine | HMDB0028898 | Organic acids and derivatives | Carboxylic acids and derivatives | Amino acids, peptides, and analogues | 0.1695 | 0.001086 |
| 1.59_653.1566m/z | 653.1566 | 1.586217 | neg | 2-(S-Glutathionyl)acetyl glutathione | HMDB0060343 | Organic acids and derivatives | Carboxylic acids and derivatives | Amino acids, peptides, and analogues | 0.430183 | 0.002005 |
| 10.84_591.3509m/z | 591.3509 | 10.83868 | neg | 17a-Ethynylestradiol | HMDB0001926 | Lipids and lipid-like molecules | Steroids and steroid derivatives | Estrane steroids | 0.208532 | 0.042152 |
| 12.50_856.5788m/z | 856.5788 | 12.50405 | pos | PC(22:5(4Z,7Z,10Z,13Z,16Z)/18:1(11Z)) | HMDB0008662 | Lipids and lipid-like molecules | Glycerophospholipids | Glycerophosphocholines | 0.685256 | 0.037443 |
| 8.27_295.2266m/z | 295.2266 | 8.2677 | pos | 11-HpODE | HMDB0062281 | Lipids and lipid-like molecules | Fatty Acyls | Lineolic acids and derivatives | 0.117296 | 0.043834 |
| 1.36_175.0477m/z | 175.0477 | 1.361683 | pos | N1-Methyl-4-pyridone-3-carboxamide | HMDB0004194 | Organoheterocyclic compounds | Pyridines and derivatives | Pyridinecarboxylic acids and derivatives | 0.24594 | 0.007239 |

**Table S6 Significantly differential metabolites in rat plasma between Shudage-4 group and model groups**

| ID | m/z | Retention time (min) | Ion mode | Metabolites | Compound ID | Super Class | Class | Sub Class | VIP | P-value |
| --- | --- | --- | --- | --- | --- | --- | --- | --- | --- | --- |
| 9.93_382.2713m/z | 382.2713 | 9.9323 | pos | Sphinganine 1-phosphate | HMDB0001383 | Lipids and lipid-like molecules | Sphingolipids | Phosphosphingolipids | 3.563792 | 0.005216 |
| 9.73_379.2482n | 380.2555 | 9.7293 | pos | Sphingosine 1-phosphate | HMDB0000277 | Lipids and lipid-like molecules | Sphingolipids | Phosphosphingolipids | 3.622921 | 0.019573 |
| 13.28_506.3604m/z | 506.3604 | 13.28398 | pos | LysoPC(P-18:1(9Z)) | HMDB0010408 | Unclassified | Unclassified | Unclassified | 0.432392 | 0.034831 |
| 6.16_433.1128m/z | 433.1128 | 6.161383 | pos | Phloretin 2'-O-glucuronide | HMDB0041768 | Phenylpropanoids and polyketides | Flavonoids | Flavonoid glycosides | 1.128939 | 5.45E-05 |
| 7.51_449.3141n | 432.3108 | 7.510567 | pos | Deoxycholic acid glycine conjugate | HMDB0000631 | Lipids and lipid-like molecules | Steroids and steroid derivatives | Bile acids, alcohols and derivatives | 1.196662 | 0.03976 |
| 7.68_459.2036m/z | 459.2036 | 7.680467 | neg | 2-Methoxy-estradiol-17b 3-glucuronide | HMDB0006765 | Lipids and lipid-like molecules | Steroids and steroid derivatives | Steroidal glycosides | 4.233605 | 1.06E-05 |
| 6.43_498.2882m/z | 498.2882 | 6.433117 | pos | Taurocholic acid | HMDB0000036 | Lipids and lipid-like molecules | Steroids and steroid derivatives | Bile acids, alcohols and derivatives | 0.950564 | 0.047478 |
| 5.27_395.1176m/z | 395.1176 | 5.26795 | neg | Estrone sulfate | HMDB0001425 | Lipids and lipid-like molecules | Steroids and steroid derivatives | Sulfated steroids | 7.907641 | 3.49E-12 |
| 4.49_463.0869m/z | 463.0869 | 4.4892 | pos | Kaempferol 3-glucuronide | HMDB0029500 | Phenylpropanoids and polyketides | Flavonoids | Flavonoid glycosides | 1.462454 | 2.58E-06 |
| 5.12_447.0943m/z | 447.0943 | 5.1213 | neg | Naringenin 4'-O-glucuronide | HMDB0041759 | Phenylpropanoids and polyketides | Flavonoids | Flavonoid glycosides | 1.420332 | 2.51E-07 |
| 4.67_246.1487m/z | 246.1487 | 4.668483 | pos | Bisphenol A | HMDB0032133 | Benzenoids | Benzene and substituted derivatives | Diphenylmethanes | 0.714387 | 1.09E-06 |
| 1.01_245.0767m/z | 245.0767 | 1.011917 | pos | Uridine | HMDB0000296 | Nucleosides, nucleotides, and analogues | Pyrimidine nucleosides | Unclassified | 0.406857 | 0.004635 |
| 7.52_459.2036m/z | 459.2036 | 7.5192 | neg | 4-Hydroxyandrostenedione glucuronide | HMDB0010315 | Lipids and lipid-like molecules | Steroids and steroid derivatives | Cholestane steroids | 3.617081 | 2.13E-06 |
| 0.68_174.0878m/z | 174.0878 | 0.683917 | neg | Citrulline | HMDB0000904 | Organic acids and derivatives | Carboxylic acids and derivatives | Amino acids, peptides, and analogues | 0.440458 | 0.029561 |
| 12.50_563.2646m/z | 563.2646 | 12.50405 | pos | Protoporphyrin IX | HMDB0000241 | Organoheterocyclic compounds | Tetrapyrroles and derivatives | Porphyrins | 2.421401 | 0.010696 |
| 4.56_230.9969m/z | 230.9969 | 4.555917 | neg | Hydroxymethoxyphenylcarboxylic acid-O-sulphate | HMDB0060000 | Benzenoids | Phenols | Methoxyphenols | 2.360989 | 1.43E-06 |
| 0.79_243.0624m/z | 243.0624 | 0.787467 | neg | Pseudouridine | HMDB0000767 | Nucleosides, nucleotides, and analogues | Nucleoside and nucleotide analogues | Unclassified | 0.479777 | 0.017855 |
| 1.52_232.1289m/z | 232.1289 | 1.515033 | pos | Asparaginyl-Valine | HMDB0028744 | Organic acids and derivatives | Carboxylic acids and derivatives | Amino acids, peptides, and analogues | 0.21336 | 0.035111 |
| 0.83_192.0264n | 191.0193 | 0.830067 | neg | Isocitric acid | HMDB0000193 | Organic acids and derivatives | Carboxylic acids and derivatives | Tricarboxylic acids and derivatives | 4.285097 | 0.005583 |
| 0.80_160.1330m/z | 160.133 | 0.796917 | pos | DL-2-Aminooctanoic acid | HMDB0000991 | Organic acids and derivatives | Carboxylic acids and derivatives | Amino acids, peptides, and analogues | 2.120698 | 0.001596 |
| 5.27_497.0566m/z | 497.0566 | 5.26795 | neg | Norepinephrine sulfate | HMDB0002062 | Organic acids and derivatives | Organic sulfuric acids and derivatives | Arylsulfates | 4.15227 | 1.31E-11 |
| 6.03_151.0393m/z | 151.0393 | 6.033633 | neg | 2-Methoxybenzoic acid | HMDB0032604 | Benzenoids | Benzene and substituted derivatives | Benzoic acids and derivatives | 0.897494 | 1.71E-06 |
| 1.12_164.0472n | 182.0811 | 1.118033 | pos | m-Coumaric acid | HMDB0001713 | Phenylpropanoids and polyketides | Cinnamic acids and derivatives | Hydroxycinnamic acids and derivatives | 2.569638 | 0.048102 |
| 11.45_338.3050m/z | 338.305 | 11.45482 | pos | 5alpha-Pregnane-3alpha,20alpha-diol | HMDB0060409 | Lipids and lipid-like molecules | Steroids and steroid derivatives | Pregnane steroids | 0.552855 | 0.019608 |
| 1.12_135.0686n | 136.0756 | 1.118033 | pos | 2-Phenylacetamide | HMDB0010715 | Benzenoids | Benzene and substituted derivatives | Phenylacetamides | 1.168494 | 0.033731 |
| 13.96_124.0869m/z | 124.0869 | 13.95712 | pos | L-Histidinol | HMDB0003431 | Organic nitrogen compounds | Organonitrogen compounds | Amines | 1.60793 | 7.93E-05 |
| 8.08_263.1294m/z | 263.1294 | 8.082617 | neg | gamma-CEHC | HMDB0001931 | Organoheterocyclic compounds | Benzopyrans | 1-benzopyrans | 2.006806 | 1.35E-09 |
| 10.28_424.3416m/z | 424.3416 | 10.28005 | pos | trans-2-Tetradecenoylcarnitine | HMDB0013329 | Organic acids and derivatives | Hydroxy acids and derivatives | Beta hydroxy acids and derivatives | 2.160934 | 0.048238 |
| 4.97_292.1654m/z | 292.1654 | 4.968167 | pos | Lysyl-Tyrosine | HMDB0028963 | Organic acids and derivatives | Carboxylic acids and derivatives | Amino acids, peptides, and analogues | 0.380653 | 0.047032 |
| 9.82_249.1500m/z | 249.15 | 9.821167 | neg | 2,4-Di-tert-butylphenol | HMDB0013816 | Benzenoids | Benzene and substituted derivatives | Phenylpropanes | 2.777268 | 0.003139 |
| 1.12_268.1038m/z | 268.1038 | 1.118033 | pos | Adenosine | HMDB0000050 | Nucleosides, nucleotides, and analogues | Purine nucleosides | Unclassified | 0.900641 | 0.032244 |
| 1.64_261.0072m/z | 261.0072 | 1.641483 | neg | Dihydrocaffeic acid 3-sulfate | HMDB0041721 | Organic acids and derivatives | Organic sulfuric acids and derivatives | Arylsulfates | 0.566843 | 0.016803 |
| 1.06_126.0662m/z | 126.0662 | 1.055433 | pos | 5-Methylcytosine | HMDB0002894 | Organoheterocyclic compounds | Diazines | Pyrimidines and pyrimidine derivatives | 0.745061 | 0.010408 |
| 3.39_246.9920m/z | 246.992 | 3.39165 | neg | Vanillic acid 4-sulfate | HMDB0041788 | Benzenoids | Benzene and substituted derivatives | Benzoic acids and derivatives | 0.860459 | 3.16E-05 |
| 11.85_627.5339m/z | 627.5339 | 11.84975 | pos | DG(18:0/20:4(5Z,8Z,11Z,14Z)/0:0) | HMDB0007170 | Lipids and lipid-like molecules | Glycerolipids | Diradylglycerols | 7.144102 | 0.032011 |
| 2.21_144.0655m/z | 144.0655 | 2.2062 | pos | Acetylhomoserine | HMDB0029423 | Organic acids and derivatives | Carboxylic acids and derivatives | Amino acids, peptides, and analogues | 0.16853 | 0.042652 |
| 5.97_221.0806m/z | 221.0806 | 5.965417 | pos | 3,4,5-Trimethoxycinnamic acid | HMDB0002511 | Phenylpropanoids and polyketides | Cinnamic acids and derivatives | Hydroxycinnamic acids and derivatives | 1.201926 | 2.28E-05 |
| 1.12_123.0441m/z | 123.0441 | 1.118033 | pos | 2-Hydroxybenzaldehyde | HMDB0034170 | Organic oxygen compounds | Organooxygen compounds | Carbonyl compounds | 0.890509 | 0.002988 |
| 4.94_171.0657m/z | 171.0657 | 4.939467 | neg | 3-Hydroxysuberic acid | HMDB0000325 | Organic acids and derivatives | Hydroxy acids and derivatives | Medium-chain hydroxy acids and derivatives | 0.616045 | 0.002445 |
| 1.06_258.1084m/z | 258.1084 | 1.055433 | pos | 5-Methylcytidine | HMDB0000982 | Nucleosides, nucleotides, and analogues | Pyrimidine nucleosides | Unclassified | 0.66998 | 0.022746 |
| 7.70_159.1020m/z | 159.102 | 7.6986 | neg | 7-Hydroxyoctanoic acid | HMDB0000486 | Organic acids and derivatives | Hydroxy acids and derivatives | Medium-chain hydroxy acids and derivatives | 0.118698 | 0.009866 |
| 3.59_144.0655m/z | 144.0655 | 3.591433 | pos | Aminoadipic acid | HMDB0000510 | Organic acids and derivatives | Carboxylic acids and derivatives | Amino acids, peptides, and analogues | 0.199424 | 0.03971 |
| 9.87_239.1655m/z | 239.1655 | 9.871867 | neg | 4-(2,6,6-Trimethyl-1-cyclohexen-1-yl)-2-butanone | HMDB0032913 | Lipids and lipid-like molecules | Prenol lipids | Sesquiterpenoids | 1.981216 | 0.034375 |
| 4.26_178.0504m/z | 178.0504 | 4.259983 | neg | Hippuric acid | HMDB0000714 | Benzenoids | Benzene and substituted derivatives | Benzoic acids and derivatives | 1.385113 | 0.016498 |
| 1.05_192.0264n | 191.0193 | 1.045383 | neg | Diketogulonic acid | HMDB0005971 | Organic oxygen compounds | Organooxygen compounds | Carbohydrates and carbohydrate conjugates | 5.819605 | 0.000164 |
| 7.52_267.1742m/z | 267.1742 | 7.523467 | pos | Boldione | HMDB0003422 | Lipids and lipid-like molecules | Steroids and steroid derivatives | Androstane steroids | 1.270901 | 6.71E-07 |
| 1.16_143.0338m/z | 143.0338 | 1.1621 | pos | Oxoadipic acid | HMDB0000225 | Organic acids and derivatives | Keto acids and derivatives | Medium-chain keto acids and derivatives | 1.594425 | 2.97E-05 |
| 5.91_185.0816m/z | 185.0816 | 5.912617 | neg | 2-Propyl-2,4-pentadienoic acid | HMDB0060682 | Lipids and lipid-like molecules | Fatty Acyls | Fatty acids and conjugates | 0.480779 | 0.000719 |
| 4.33_165.0187m/z | 165.0187 | 4.333533 | neg | Phthalic acid | HMDB0002107 | Benzenoids | Benzene and substituted derivatives | Benzoic acids and derivatives | 0.600323 | 0.017748 |
| 4.64_209.0453m/z | 209.0453 | 4.642683 | neg | 3-(3,4-Dihydroxy-5-methoxy)-2-propenoic acid | HMDB0035484 | Phenylpropanoids and polyketides | Cinnamic acids and derivatives | Hydroxycinnamic acids and derivatives | 2.911378 | 4.54E-05 |
| 4.79_173.0814m/z | 173.0814 | 4.791317 | neg | Cyclohexanecarboxylic acid | HMDB0031342 | Organic acids and derivatives | Carboxylic acids and derivatives | Carboxylic acids | 0.779638 | 0.02147 |
| 5.20_273.0443m/z | 273.0443 | 5.20445 | neg | 4-Hydroxy-5-(phenyl)-valeric acid-O-sulphate | HMDB0059981 | Benzenoids | Benzene and substituted derivatives | Unclassified | 0.763031 | 7.62E-06 |
| 13.76_267.2680m/z | 267.268 | 13.76133 | pos | Stearic acid | HMDB0000827 | Lipids and lipid-like molecules | Fatty Acyls | Fatty acids and conjugates | 1.005993 | 5.43E-06 |
| 2.21_164.0710m/z | 164.071 | 2.214233 | neg | L-Phenylalanine | HMDB0000159 | Organic acids and derivatives | Carboxylic acids and derivatives | Amino acids, peptides, and analogues | 0.595453 | 0.021207 |
| 4.90_447.0942m/z | 447.0942 | 4.8964 | neg | Naringenin 5-O-glucuronide | HMDB0041760 | Phenylpropanoids and polyketides | Flavonoids | Flavonoid glycosides | 0.840498 | 0.004114 |
| 7.13_214.1800m/z | 214.18 | 7.128983 | pos | alpha-Terpineol acetate | HMDB0032051 | Lipids and lipid-like molecules | Prenol lipids | Monoterpenoids | 1.002291 | 0.003282 |
| 5.55_224.0928m/z | 224.0928 | 5.545233 | neg | 2(N)-Methyl-norsalsolinol | HMDB0001189 | Organoheterocyclic compounds | Tetrahydroisoquinolines | Unclassified | 0.368848 | 0.045044 |
| 5.24_187.0972m/z | 187.0972 | 5.236283 | neg | 4-ene-Valproic acid | HMDB0013897 | Lipids and lipid-like molecules | Fatty Acyls | Fatty acids and conjugates | 1.263092 | 0.002514 |
| 0.76_174.1004n | 175.1076 | 0.755917 | pos | Glycyl-Valine | HMDB0028854 | Organic acids and derivatives | Carboxylic acids and derivatives | Amino acids, peptides, and analogues | 0.744523 | 0.000554 |
| 1.45_163.0389m/z | 163.0389 | 1.447617 | pos | (E)-3-(2,3-Dihydroxyphenyl)-2-propenoic acid | HMDB0032057 | Phenylpropanoids and polyketides | Cinnamic acids and derivatives | Hydroxycinnamic acids and derivatives | 0.654167 | 0.039126 |
| 4.46_447.2067m/z | 447.2067 | 4.45855 | neg | 1-Methyl-6-phenyl-1H-imidazo[4,5-b]pyridin-2-amine | HMDB0041008 | Organoheterocyclic compounds | Pyridines and derivatives | Phenylpyridines | 1.492583 | 0.013248 |
| 11.94_199.1690m/z | 199.169 | 11.93697 | pos | 3-Hydroxydodecanoic acid | HMDB0000387 | Organic acids and derivatives | Hydroxy acids and derivatives | Medium-chain hydroxy acids and derivatives | 0.302941 | 0.043164 |
| 2.73_216.9811m/z | 216.9811 | 2.729317 | neg | 3-hydroxybenzoic acid-3-O-sulphate | HMDB0059968 | Organic acids and derivatives | Organic sulfuric acids and derivatives | Arylsulfates | 6.014791 | 3.46E-09 |
| 4.56_171.0657m/z | 171.0657 | 4.555917 | neg | But-2-enoic acid | HMDB0010720 | Lipids and lipid-like molecules | Fatty Acyls | Fatty acids and conjugates | 0.528907 | 0.001341 |
| 11.54_104.1072m/z | 104.1072 | 11.5425 | pos | 3-Methyl-3-buten-1-ol | HMDB0030126 | Organic oxygen compounds | Organooxygen compounds | Alcohols and polyols | 0.198435 | 0.039759 |
| 1.47_184.0604m/z | 184.0604 | 1.46965 | pos | 4-Pyridoxic acid | HMDB0000017 | Organoheterocyclic compounds | Pyridines and derivatives | Pyridinecarboxylic acids and derivatives | 0.324307 | 0.006262 |
| 5.09_188.1645m/z | 188.1645 | 5.086 | pos | 8-Methylnonenoate | HMDB0012183 | Lipids and lipid-like molecules | Fatty Acyls | Fatty acids and conjugates | 0.317713 | 0.000189 |
| 1.28_276.0180m/z | 276.018 | 1.278917 | neg | Paracetamol sulfate | HMDB0059911 | Organic acids and derivatives | Organic sulfuric acids and derivatives | Arylsulfates | 0.651863 | 0.011272 |
| 5.98_283.1689m/z | 283.1689 | 5.979233 | pos | Adrenosterone | HMDB0006772 | Lipids and lipid-like molecules | Steroids and steroid derivatives | Androstane steroids | 0.803275 | 1.95E-05 |
| 5.33_447.0919m/z | 447.0919 | 5.32815 | pos | Isoquercitrin | HMDB0037362 | Phenylpropanoids and polyketides | Flavonoids | Flavonoid glycosides | 2.58251 | 1.67E-05 |
| 4.56_259.0285m/z | 259.0285 | 4.564717 | neg | Fluorouracil | HMDB0014684 | Organoheterocyclic compounds | Diazines | Pyrimidines and pyrimidine derivatives | 2.295023 | 9.01E-07 |
| 10.21_149.0960m/z | 149.096 | 10.21247 | pos | Perillic acid | HMDB0004586 | Lipids and lipid-like molecules | Prenol lipids | Monoterpenoids | 0.530738 | 0.014847 |
| 4.55_186.1131m/z | 186.1131 | 4.5452 | neg | (±)-Pelletierine | HMDB0030325 | Organoheterocyclic compounds | Piperidines | Unclassified | 1.04606 | 2.2E-05 |
| 7.86_405.2655m/z | 405.2655 | 7.856083 | neg | 3a,6b,7a,12a-Tetrahydroxy-5b-cholanoic acid | HMDB0000399 | Organic oxygen compounds | Organooxygen compounds | Carbohydrates and carbohydrate conjugates | 2.010968 | 0.006639 |
| 9.88_167.0701m/z | 167.0701 | 9.877267 | pos | 3-(3-Hydroxyphenyl)propanoic acid | HMDB0000375 | Phenylpropanoids and polyketides | Phenylpropanoic acids | Unclassified | 0.321862 | 0.009148 |
| 5.15_163.0389m/z | 163.0389 | 5.1451 | pos | Umbelliferone | HMDB0029865 | Phenylpropanoids and polyketides | Coumarins and derivatives | Hydroxycoumarins | 0.367637 | 0.03425 |
| 4.77_287.0549m/z | 287.0549 | 4.766917 | pos | Luteolin | HMDB0005800 | Phenylpropanoids and polyketides | Flavonoids | Flavones | 2.286565 | 0.000118 |
| 12.02_223.2054m/z | 223.2054 | 12.02277 | pos | Farnesol | HMDB0004305 | Lipids and lipid-like molecules | Prenol lipids | Sesquiterpenoids | 0.672282 | 0.001988 |
| 5.29_144.0655m/z | 144.0655 | 5.293067 | pos | Methyl 2-furoate | HMDB0029750 | Organoheterocyclic compounds | Furans | Furoic acid and derivatives | 0.195686 | 0.016367 |
| 1.06_192.0268n | 215.016 | 1.055433 | pos | Citric acid | HMDB0000094 | Organic acids and derivatives | Carboxylic acids and derivatives | Tricarboxylic acids and derivatives | 1.95795 | 0.001904 |
| 11.21_339.2548m/z | 339.2548 | 11.21477 | neg | Linalyl oxide | HMDB0035907 | Organoheterocyclic compounds | Tetrahydrofurans | Unclassified | 0.549859 | 0.048534 |
| 12.35_327.2336m/z | 327.2336 | 12.35052 | neg | Docosahexaenoic acid | HMDB0002183 | Lipids and lipid-like molecules | Fatty Acyls | Fatty acids and conjugates | 0.663447 | 0.009473 |
| 13.20_152.9947m/z | 152.9947 | 13.19578 | pos | Dihydroxyacetone phosphate | HMDB0001473 | Organic oxygen compounds | Organooxygen compounds | Carbohydrates and carbohydrate conjugates | 2.404856 | 8.54E-06 |
| 0.70_137.0708m/z | 137.0708 | 0.6975 | pos | N-Methylnicotinamide | HMDB0003152 | Organoheterocyclic compounds | Pyridines and derivatives | Pyridinecarboxylic acids and derivatives | 0.753717 | 0.049214 |
| 4.56_151.0393m/z | 151.0393 | 4.555917 | neg | 3,4-Dihydroxyphenylglycol | HMDB0000318 | Benzenoids | Phenols | Benzenediols | 0.417227 | 1.72E-05 |
| 4.70_163.0394m/z | 163.0394 | 4.6992 | neg | Phenylpyruvic acid | HMDB0000205 | Benzenoids | Benzene and substituted derivatives | Phenylpyruvic acid derivatives | 0.620108 | 0.001234 |
| 5.05_259.0468m/z | 259.0468 | 5.049667 | neg | Citraconic acid | HMDB0000634 | Lipids and lipid-like molecules | Fatty Acyls | Fatty acids and conjugates | 1.759087 | 2.42E-07 |
| 8.08_193.0868m/z | 193.0868 | 8.082617 | neg | Cuminaldehyde | HMDB0002214 | Lipids and lipid-like molecules | Prenol lipids | Monoterpenoids | 0.544451 | 0.01177 |
| 3.29_168.0655m/z | 168.0655 | 3.294083 | pos | Phenylglyoxylic acid | HMDB0001587 | Benzenoids | Benzene and substituted derivatives | Benzoyl derivatives | 0.150713 | 0.043978 |
| 5.88_269.0457m/z | 269.0457 | 5.877883 | neg | Genistein | HMDB0003217 | Phenylpropanoids and polyketides | Isoflavonoids | Isoflav-2-enes | 1.04625 | 1.81E-07 |
| 1.87_204.0267m/z | 204.0267 | 1.872617 | pos | 2-Phospho-D-glyceric acid | HMDB0003391 | Organic oxygen compounds | Organooxygen compounds | Carbohydrates and carbohydrate conjugates | 0.184927 | 0.007099 |
| 13.20_203.0215m/z | 203.0215 | 13.19578 | pos | Imidazole acetol-phosphate | HMDB0012236 | Organic acids and derivatives | Organic phosphoric acids and derivatives | Phosphate esters | 0.281962 | 0.001026 |
| 2.56_204.0267m/z | 204.0267 | 2.560467 | pos | 2-Phosphoglyceric acid | HMDB0000362 | Organic oxygen compounds | Organooxygen compounds | Carbohydrates and carbohydrate conjugates | 0.128274 | 0.043197 |
| 5.18_189.1128m/z | 189.1128 | 5.1824 | neg | Caprylic acid | HMDB0000482 | Lipids and lipid-like molecules | Fatty Acyls | Fatty acids and conjugates | 0.274792 | 0.001786 |
| 5.40_228.0280m/z | 228.028 | 5.3976 | neg | 5-Phosphoribosylamine | HMDB0001128 | Organic oxygen compounds | Organooxygen compounds | Carbohydrates and carbohydrate conjugates | 8.822922 | 5.9E-06 |
| 0.56_172.9961m/z | 172.9961 | 0.556 | pos | (S)-Ureidoglycolic acid | HMDB0001005 | Organic acids and derivatives | Carboxylic acids and derivatives | Amino acids, peptides, and analogues | 0.448118 | 9.5E-05 |
| 1.12_121.0649m/z | 121.0649 | 1.118033 | pos | Tyrosol | HMDB0004284 | Benzenoids | Phenols | Tyrosols and derivatives | 0.367521 | 0.000116 |
| 4.99_298.1284m/z | 298.1284 | 4.99225 | pos | Phenethylamine glucuronide | HMDB0010323 | Organic oxygen compounds | Organooxygen compounds | Carbohydrates and carbohydrate conjugates | 1.305169 | 3.79E-09 |
| 0.99_160.1331m/z | 160.1331 | 0.9902 | pos | Methyl cyclohexanecarboxylate | HMDB0031343 | Organic acids and derivatives | Carboxylic acids and derivatives | Carboxylic acid derivatives | 1.848927 | 0.003953 |
| 1.98_207.0764m/z | 207.0764 | 1.981217 | pos | 1-Nitronaphthalene-5,6-oxide | HMDB0060331 | Benzenoids | Naphthalenes | Nitronaphthalenes | 0.161111 | 0.002863 |
| 11.05_104.1071m/z | 104.1071 | 11.04965 | pos | Prenol | HMDB0030124 | Organic oxygen compounds | Organooxygen compounds | Alcohols and polyols | 0.234558 | 5.84E-05 |
| 12.48_180.9895m/z | 180.9895 | 12.48145 | pos | Dihydroxyacetone Phosphate Acyl Ester | HMDB0011750 | Organic oxygen compounds | Organooxygen compounds | Carbonyl compounds | 0.309573 | 0.007167 |
| 4.60_201.1129m/z | 201.1129 | 4.596817 | neg | Dihydro-5-pentyl-2(3H)-furanone | HMDB0031531 | Organoheterocyclic compounds | Lactones | Gamma butyrolactones | 0.316702 | 0.009583 |
| 4.67_259.1516m/z | 259.1516 | 4.668483 | pos | Tetrahydrobiopterin | HMDB0000027 | Organoheterocyclic compounds | Pteridines and derivatives | Pterins and derivatives | 0.48607 | 0.043143 |
| 5.17_151.1117m/z | 151.1117 | 5.173683 | pos | Cuminyl alcohol | HMDB0031817 | Lipids and lipid-like molecules | Prenol lipids | Monoterpenoids | 0.140802 | 0.043426 |
| 6.64_361.1124m/z | 361.1124 | 6.640933 | neg | 8-Hydroxy-7-methylguanine | HMDB0006037 | Organoheterocyclic compounds | Imidazopyrimidines | Purines and purine derivatives | 7.597444 | 8.38E-05 |
| 9.66_149.0960m/z | 149.096 | 9.661333 | pos | Anethole | HMDB0030837 | Benzenoids | Phenol ethers | Anisoles | 0.315289 | 0.005094 |
| 13.24_180.9896m/z | 180.9896 | 13.23957 | pos | cis,cis-Muconic acid | HMDB0006331 | Lipids and lipid-like molecules | Fatty Acyls | Fatty acids and conjugates | 1.864906 | 2.18E-05 |
| 4.59_244.0231m/z | 244.0231 | 4.585217 | neg | O-Phosphohomoserine | HMDB0003484 | Organic acids and derivatives | Carboxylic acids and derivatives | Amino acids, peptides, and analogues | 2.191515 | 1.6E-07 |
| 5.26_240.1593m/z | 240.1593 | 5.2613 | pos | 2-Hydroxyibuprofen | HMDB0060920 | Phenylpropanoids and polyketides | Phenylpropanoic acids | Unclassified | 0.315076 | 9.53E-05 |
| 11.36_295.2284m/z | 295.2284 | 11.35835 | neg | Alpha-dimorphecolic acid | HMDB0004670 | Lipids and lipid-like molecules | Fatty Acyls | Lineolic acids and derivatives | 0.879279 | 0.046489 |
| 3.78_267.0514m/z | 267.0514 | 3.7827 | neg | Diphenol glucuronide | HMDB0059998 | Organic oxygen compounds | Organooxygen compounds | Carbohydrates and carbohydrate conjugates | 0.538821 | 2.08E-05 |
| 4.61_190.0498m/z | 190.0498 | 4.613317 | pos | 1-Nitronaphthalene-7,8-oxide | HMDB0060332 | Benzenoids | Naphthalenes | Nitronaphthalenes | 0.371685 | 0.002427 |
| 5.21_259.0467m/z | 259.0467 | 5.213367 | neg | Mesaconic acid | HMDB0000749 | Lipids and lipid-like molecules | Fatty Acyls | Fatty acids and conjugates | 2.581581 | 1.13E-06 |
| 5.40_263.0598m/z | 263.0598 | 5.3976 | neg | 5,6-Dihydro-5-fluorouracil | HMDB0060402 | Organoheterocyclic compounds | Diazines | Pyrimidines and pyrimidine derivatives | 0.257972 | 0.005627 |
| 0.68_188.1756m/z | 188.1756 | 0.677467 | pos | N1-Acetylspermidine | HMDB0001276 | Organic acids and derivatives | Carboximidic acids and derivatives | Carboximidic acids | 0.556254 | 0.002684 |
| 10.18_144.0654m/z | 144.0654 | 10.18415 | pos | 1,2,3-Trihydroxybenzene | HMDB0013674 | Benzenoids | Phenols | Benzenetriols and derivatives | 0.230581 | 0.013214 |
| 4.61_245.0309m/z | 245.0309 | 4.607583 | neg | 4-Fumarylacetoacetic acid | HMDB0001268 | Organic acids and derivatives | Keto acids and derivatives | Medium-chain keto acids and derivatives | 2.760978 | 1.51E-09 |
| 5.33_244.0232m/z | 244.0232 | 5.333783 | neg | O-Phosphothreonine | HMDB0011185 | Organic acids and derivatives | Carboxylic acids and derivatives | Amino acids, peptides, and analogues | 2.268277 | 4.8E-06 |
| 7.02_777.6931m/z | 777.6931 | 7.023883 | pos | Thyroxine | HMDB0000248 | Organic acids and derivatives | Carboxylic acids and derivatives | Amino acids, peptides, and analogues | 0.619244 | 0.022659 |
| 7.64_187.1336m/z | 187.1336 | 7.644567 | neg | 2,6-Dimethyl-4-heptanone | HMDB0031417 | Organic oxygen compounds | Organooxygen compounds | Carbonyl compounds | 0.23669 | 0.049845 |
| 9.77_144.0653m/z | 144.0653 | 9.77395 | pos | N-methyl-L-glutamic Acid | HMDB0062660 | Organic acids and derivatives | Carboxylic acids and derivatives | Amino acids, peptides, and analogues | 0.109 | 0.042879 |
| 0.68_266.0883m/z | 266.0883 | 0.683917 | neg | N-Acetylgalactosamine | HMDB0000212 | Organic oxygen compounds | Organooxygen compounds | Carbohydrates and carbohydrate conjugates | 0.514022 | 0.032289 |
| 2.74_184.0967m/z | 184.0967 | 2.735283 | pos | 3-(2-Hydroxyphenyl)propanoic acid | HMDB0033752 | Phenylpropanoids and polyketides | Phenylpropanoic acids | Unclassified | 0.533086 | 0.000989 |
| 3.91_113.1076m/z | 113.1076 | 3.914167 | pos | N-Acetylputrescine | HMDB0002064 | Organic acids and derivatives | Carboximidic acids and derivatives | Carboximidic acids | 0.342774 | 4.02E-06 |
| 5.07_268.1040m/z | 268.104 | 5.07315 | pos | Deoxyguanosine | HMDB0000085 | Nucleosides, nucleotides, and analogues | Purine nucleosides | Purine 2'-deoxyribonucleosides | 0.258331 | 0.014484 |
| 5.19_186.0760m/z | 186.076 | 5.1851 | pos | 3,4-Dihydroxybenzeneacetic acid | HMDB0001336 | Benzenoids | Phenols | Benzenediols | 0.372285 | 0.000386 |
| 5.32_271.0597m/z | 271.0597 | 5.318817 | pos | Apigenin | HMDB0002124 | Phenylpropanoids and polyketides | Flavonoids | Flavones | 0.873633 | 2.52E-05 |
| 5.41_186.0761m/z | 186.0761 | 5.4097 | pos | Homogentisic acid | HMDB0000130 | Benzenoids | Benzene and substituted derivatives | Phenylacetic acids | 0.278577 | 0.004881 |
| 0.70_417.0018m/z | 417.0018 | 0.6975 | pos | 6-Methylthiopurine 5'-monophosphate ribonucleotide | HMDB0060414 | Nucleosides, nucleotides, and analogues | Purine nucleotides | Purine ribonucleotides | 1.794147 | 0.009441 |
| 0.79_224.0234m/z | 224.0234 | 0.787467 | neg | S-Carboxymethyl-L-cysteine | HMDB0029415 | Organic acids and derivatives | Carboxylic acids and derivatives | Amino acids, peptides, and analogues | 0.618049 | 0.046979 |
| 1.09_164.0380m/z | 164.038 | 1.088183 | neg | Methionine sulfoxide | HMDB0002005 | Organic acids and derivatives | Carboxylic acids and derivatives | Amino acids, peptides, and analogues | 0.376791 | 0.004592 |
| 13.06_813.6821m/z | 813.6821 | 13.0648 | pos | SM(d18:1/24:1(15Z)) | HMDB0012107 | Lipids and lipid-like molecules | Sphingolipids | Phosphosphingolipids | 18.94837 | 0.000673 |
| 13.24_338.9892m/z | 338.9892 | 13.2358 | neg | Alpha-D-Glucose 1,6-bisphosphate | HMDB0003514 | Organic oxygen compounds | Organooxygen compounds | Carbohydrates and carbohydrate conjugates | 0.588937 | 0.001089 |
| 4.23_263.0782m/z | 263.0782 | 4.226717 | neg | Ethylmalonic acid | HMDB0000622 | Lipids and lipid-like molecules | Fatty Acyls | Fatty acids and conjugates | 0.379765 | 0.001791 |
| 4.23_385.1147m/z | 385.1147 | 4.226717 | neg | trans-isoeugenol-O-glucuronide | HMDB0060021 | Organic oxygen compounds | Organooxygen compounds | Carbohydrates and carbohydrate conjugates | 0.265955 | 0.040933 |
| 4.31_186.0760m/z | 186.076 | 4.306383 | pos | Vanillic acid | HMDB0000484 | Benzenoids | Benzene and substituted derivatives | Benzoic acids and derivatives | 0.249485 | 0.049504 |
| 8.30_310.1130m/z | 310.113 | 8.304333 | pos | N-Acetylneuraminic acid | HMDB0000230 | Organic oxygen compounds | Organooxygen compounds | Carbohydrates and carbohydrate conjugates | 0.17528 | 0.010662 |
| 0.58_143.0338m/z | 143.0338 | 0.578 | pos | 3-Oxoadipic acid | HMDB0000398 | Organic acids and derivatives | Keto acids and derivatives | Medium-chain keto acids and derivatives | 0.444422 | 0.004864 |
| 1.06_156.0058n | 139.0025 | 1.055433 | pos | 2,5-Furandicarboxylic acid | HMDB0004812 | Organoheterocyclic compounds | Furans | Furoic acid and derivatives | 0.456435 | 0.002459 |
| 1.06_242.1132m/z | 242.1132 | 1.055433 | pos | L-3-Hydroxykynurenine | HMDB0011631 | Organic oxygen compounds | Organooxygen compounds | Carbonyl compounds | 0.401201 | 0.012098 |
| 11.04_293.2128m/z | 293.2128 | 11.03955 | neg | 9-OxoODE | HMDB0004669 | Lipids and lipid-like molecules | Fatty Acyls | Lineolic acids and derivatives | 0.556136 | 0.043816 |
| 13.26_399.0086m/z | 399.0086 | 13.25658 | neg | D-Erythrose 4-phosphate | HMDB0001321 | Organic oxygen compounds | Organooxygen compounds | Carbohydrates and carbohydrate conjugates | 0.602599 | 0.003538 |
| 13.39_137.0000m/z | 137 | 13.39332 | pos | 2-Furanmethanol | HMDB0013742 | Organoheterocyclic compounds | Heteroaromatic compounds | Unclassified | 0.259399 | 3.01E-05 |
| 4.50_158.0816m/z | 158.0816 | 4.5008 | neg | Valerylglycine | HMDB0000927 | Organic acids and derivatives | Carboxylic acids and derivatives | Amino acids, peptides, and analogues | 0.341285 | 0.031241 |
| 5.25_379.1208m/z | 379.1208 | 5.252383 | pos | Citalopram N-oxide | HMDB0060654 | Benzenoids | Benzene and substituted derivatives | Phenylbutylamines | 3.186687 | 2.42E-12 |
| 5.48_295.1191m/z | 295.1191 | 5.48145 | neg | Ubiquinone-1 | HMDB0002012 | Lipids and lipid-like molecules | Prenol lipids | Quinone and hydroquinone lipids | 0.479289 | 0.000295 |
| 6.90_386.1451m/z | 386.1451 | 6.902317 | pos | 3-O-Feruloylquinic acid | HMDB0030669 | Organic oxygen compounds | Organooxygen compounds | Alcohols and polyols | 0.483968 | 0.000293 |
| 8.16_219.1392m/z | 219.1392 | 8.157333 | neg | 2-Ethyl-5-methylfuran | HMDB0029728 | Organoheterocyclic compounds | Heteroaromatic compounds | Unclassified | 0.708134 | 3.92E-15 |
| 9.73_107.0857m/z | 107.0857 | 9.7293 | pos | Ethylbenzene | HMDB0059905 | Benzenoids | Benzene and substituted derivatives | Unclassified | 0.180807 | 0.048224 |
| 0.99_133.0972m/z | 133.0972 | 0.9902 | pos | D-Proline | HMDB0003411 | Organic acids and derivatives | Carboxylic acids and derivatives | Amino acids, peptides, and analogues | 0.120662 | 0.042739 |
| 11.44_344.0859m/z | 344.0859 | 11.44108 | neg | 8-Hydroxyguanosine | HMDB0002044 | Nucleosides, nucleotides, and analogues | Purine nucleosides | Unclassified | 0.356111 | 0.032287 |
| 11.79_461.3648m/z | 461.3648 | 11.78952 | neg | Gamma-Tocopherol | HMDB0001492 | Lipids and lipid-like molecules | Prenol lipids | Quinone and hydroquinone lipids | 0.774579 | 0.017547 |
| 14.83_198.0170m/z | 198.017 | 14.83202 | pos | O-Phospho-4-hydroxy-L-threonine | HMDB0006802 | Organic acids and derivatives | Carboxylic acids and derivatives | Amino acids, peptides, and analogues | 0.495174 | 0.040335 |
| 3.93_153.1273m/z | 153.1273 | 3.92665 | pos | Pulegone | HMDB0035604 | Lipids and lipid-like molecules | Prenol lipids | Monoterpenoids | 0.458262 | 8.02E-06 |
| 5.15_132.0655m/z | 132.0655 | 5.1451 | pos | 5-Aminolevulinic acid | HMDB0001149 | Organic acids and derivatives | Carboxylic acids and derivatives | Amino acids, peptides, and analogues | 0.53535 | 4.27E-08 |
| 5.51_179.0557m/z | 179.0557 | 5.5096 | neg | Alpha-D-Glucose | HMDB0003345 | Organic oxygen compounds | Organooxygen compounds | Carbohydrates and carbohydrate conjugates | 0.212389 | 0.004971 |
| 6.95_235.1341m/z | 235.1341 | 6.948233 | neg | beta-Damascenone | HMDB0013804 | Organic oxygen compounds | Organooxygen compounds | Carbonyl compounds | 0.39864 | 0.001249 |
| 7.31_132.0655m/z | 132.0655 | 7.3147 | pos | 4-Hydroxyproline | HMDB0000725 | Organic acids and derivatives | Carboxylic acids and derivatives | Amino acids, peptides, and analogues | 0.400677 | 0.000179 |
| 7.89_179.0557m/z | 179.0557 | 7.89 | neg | scyllo-Inositol | HMDB0006088 | Organic oxygen compounds | Organooxygen compounds | Alcohols and polyols | 0.292374 | 0.016935 |
| 8.21_179.0557m/z | 179.0557 | 8.206083 | neg | L-Gulose | HMDB0012326 | Organic oxygen compounds | Organooxygen compounds | Carbohydrates and carbohydrate conjugates | 0.288676 | 0.008399 |
| 1.56_115.0392m/z | 115.0392 | 1.562867 | pos | Methylsuccinic acid | HMDB0001844 | Lipids and lipid-like molecules | Fatty Acyls | Fatty acids and conjugates | 0.20761 | 0.002386 |
| 10.10_392.2937n | 437.2919 | 10.10057 | neg | Murocholic acid | HMDB0000811 | Lipids and lipid-like molecules | Steroids and steroid derivatives | Bile acids, alcohols and derivatives | 2.274707 | 0.043081 |
| 3.25_188.9860m/z | 188.986 | 3.249767 | neg | Pyrocatechol sulfate | HMDB0059724 | Organic acids and derivatives | Organic sulfuric acids and derivatives | Arylsulfates | 0.794671 | 0.006645 |
| 3.41_125.0962m/z | 125.0962 | 3.411717 | pos | 2-ene-Valproic acid | HMDB0013902 | Lipids and lipid-like molecules | Fatty Acyls | Fatty acids and conjugates | 0.224239 | 0.001957 |
| 4.73_545.0583m/z | 545.0583 | 4.732617 | neg | dTDP-D-glucose | HMDB0001328 | Nucleosides, nucleotides, and analogues | Pyrimidine nucleotides | Pyrimidine nucleotide sugars | 0.753217 | 4.62E-06 |
| 4.93_186.0760m/z | 186.076 | 4.933117 | pos | 3-Hydroxymandelic acid | HMDB0000750 | Benzenoids | Phenols | 1-hydroxy-4-unsubstituted benzenoids | 0.167045 | 0.036646 |
| 5.11_179.0557m/z | 179.0557 | 5.1123 | neg | D-chiro-inositol | HMDB0240209 | Organic oxygen compounds | Organooxygen compounds | Alcohols and polyols | 0.597772 | 2.68E-08 |
| 5.44_367.0033m/z | 367.0033 | 5.4448 | neg | 6-Thiourate | HMDB0060417 | Organoheterocyclic compounds | Imidazopyrimidines | Purines and purine derivatives | 0.713567 | 1.52E-08 |
| 6.16_179.0557m/z | 179.0557 | 6.163467 | neg | D-Mannose | HMDB0000169 | Organic oxygen compounds | Organooxygen compounds | Carbohydrates and carbohydrate conjugates | 0.278048 | 0.008925 |
| 8.05_130.0862m/z | 130.0862 | 8.05135 | pos | 2-Pyrrolidineacetic acid | HMDB0029444 | Organoheterocyclic compounds | Pyrrolidines | Unclassified | 0.199182 | 0.009723 |
| 8.66_179.0557m/z | 179.0557 | 8.65785 | neg | Beta-D-Galactose | HMDB0003449 | Organic oxygen compounds | Organooxygen compounds | Carbohydrates and carbohydrate conjugates | 0.263912 | 0.036159 |
| 10.60_833.5204m/z | 833.5204 | 10.5958 | neg | PI(16:1(9Z)/18:1(9Z)) | HMDB0009801 | Lipids and lipid-like molecules | Glycerophospholipids | Glycerophosphoinositols | 2.009075 | 0.003914 |
| 6.62_525.2828m/z | 525.2828 | 6.621467 | neg | Desmosine | HMDB0000572 | Organic acids and derivatives | Carboxylic acids and derivatives | Tetracarboxylic acids and derivatives | 1.381956 | 0.014043 |
| 8.12_601.2653m/z | 601.2653 | 8.123467 | pos | APC | HMDB0060661 | Organic oxygen compounds | Organooxygen compounds | Carbonyl compounds | 0.762341 | 0.005891 |
| 9.75_132.0576n | 133.0647 | 9.751317 | pos | Cinnamaldehyde | HMDB0003441 | Phenylpropanoids and polyketides | Cinnamaldehydes | Unclassified | 0.192249 | 0.032289 |
| 2.80_131.0704m/z | 131.0704 | 2.8009 | pos | 3-Methyl-2-oxovaleric acid | HMDB0000491 | Organic acids and derivatives | Keto acids and derivatives | Short-chain keto acids and derivatives | 0.111975 | 0.044686 |
| 3.50_158.0812m/z | 158.0812 | 3.4983 | pos | 2-methoxybenzene-1,3-diol | HMDB0133970 | Benzenoids | Phenols | Methoxyphenols | 0.212036 | 0.005679 |
| 0.54_887.5632m/z | 887.5632 | 0.537917 | pos | PI(18:1(9Z)/20:3(8Z,11Z,14Z)) | HMDB0009843 | Lipids and lipid-like molecules | Glycerophospholipids | Glycerophosphoinositols | 1.424708 | 0.04927 |
| 0.70_169.9856m/z | 169.9856 | 0.6975 | pos | Iminoaspartic acid | HMDB0001131 | Organic acids and derivatives | Carboxylic acids and derivatives | Dicarboxylic acids and derivatives | 0.896168 | 0.012863 |
| 1.70_166.0723m/z | 166.0723 | 1.70135 | pos | N2-Methylguanine | HMDB0006040 | Organoheterocyclic compounds | Imidazopyrimidines | Purines and purine derivatives | 0.147574 | 0.015906 |
| 2.23_113.0236m/z | 113.0236 | 2.228183 | pos | 2-Furoic acid | HMDB0000617 | Organoheterocyclic compounds | Furans | Furoic acid and derivatives | 0.282512 | 0.000735 |
| 3.88_159.0287m/z | 159.0287 | 3.882533 | pos | D-Glucurono-6,3-lactone | HMDB0006355 | Organoheterocyclic compounds | Furofurans | Isosorbides | 0.124113 | 0.000525 |
| 4.70_337.0428m/z | 337.0428 | 4.6992 | neg | Imidazoleacetic acid ribotide | HMDB0006032 | Organic oxygen compounds | Organooxygen compounds | Carbohydrates and carbohydrate conjugates | 0.515117 | 0.035859 |
| 4.89_323.0272m/z | 323.0272 | 4.887283 | neg | Uridine 5'-monophosphate | HMDB0000288 | Nucleosides, nucleotides, and analogues | Pyrimidine nucleotides | Pyrimidine ribonucleotides | 0.396766 | 0.002674 |
| 4.97_447.0943m/z | 447.0943 | 4.9729 | neg | hesperetin-7-O-glucuronide | HMDB0029203 | Organoheterocyclic compounds | Benzodioxanes | Phenylbenzodioxanes | 0.576435 | 0.019282 |
| 5.46_285.0409m/z | 285.0409 | 5.455517 | neg | Kaempferol | HMDB0005801 | Phenylpropanoids and polyketides | Flavonoids | Flavones | 0.764599 | 1.91E-13 |
| 5.91_141.0913m/z | 141.0913 | 5.912617 | neg | 5-Hydroxyvalproic acid | HMDB0013898 | Lipids and lipid-like molecules | Fatty Acyls | Fatty acids and conjugates | 0.323567 | 0.000415 |
| 0.52_150.0773m/z | 150.0773 | 0.516683 | pos | 3-Methyladenine | HMDB0011600 | Organoheterocyclic compounds | Imidazopyrimidines | Purines and purine derivatives | 0.115317 | 0.036783 |
| 0.86_331.0445m/z | 331.0445 | 0.861417 | pos | Inosinic acid | HMDB0000175 | Nucleosides, nucleotides, and analogues | Purine nucleotides | Purine ribonucleotides | 0.918682 | 0.008765 |
| 1.52_176.1281m/z | 176.1281 | 1.515033 | pos | 3-Oxovalproic acid | HMDB0060685 | Organic acids and derivatives | Keto acids and derivatives | Short-chain keto acids and derivatives | 0.108175 | 0.018524 |
| 4.43_203.0016m/z | 203.0016 | 4.427533 | neg | O-methoxycatechol-O-sulphate | HMDB0060013 | Organic acids and derivatives | Organic sulfuric acids and derivatives | Arylsulfates | 0.321134 | 0.027163 |
| 5.01_258.0389m/z | 258.0389 | 5.0062 | neg | Glucosamine 6-phosphate | HMDB0001254 | Organic oxygen compounds | Organooxygen compounds | Carbohydrates and carbohydrate conjugates | 1.392791 | 9.11E-05 |
| 5.30_195.0507m/z | 195.0507 | 5.301883 | neg | D-Ribulose | HMDB0000621 | Organic oxygen compounds | Organooxygen compounds | Carbohydrates and carbohydrate conjugates | 0.209476 | 0.000182 |
| 5.88_508.0743m/z | 508.0743 | 5.877883 | neg | Adenylsuccinic acid | HMDB0000536 | Benzenoids | Benzene and substituted derivatives | Biphenyls and derivatives | 2.217364 | 2.85E-08 |
| 1.57_181.0362m/z | 181.0362 | 1.567817 | neg | 7-Methyluric acid | HMDB0011107 | Organoheterocyclic compounds | Imidazopyrimidines | Purines and purine derivatives | 0.246717 | 0.021458 |
| 4.70_155.1178m/z | 155.1178 | 4.6977 | pos | Tyramine | HMDB0000306 | Benzenoids | Benzene and substituted derivatives | Phenethylamines | 0.072945 | 0.003061 |
| 4.82_171.1021m/z | 171.1021 | 4.8159 | neg | 1-Octen-3-one | HMDB0031309 | Organic oxygen compounds | Organooxygen compounds | Carbonyl compounds | 0.336851 | 0.014881 |
| 0.62_146.1650m/z | 146.165 | 0.616767 | pos | Spermidine | HMDB0001257 | Organic nitrogen compounds | Organonitrogen compounds | Amines | 0.557553 | 0.019226 |
| 1.53_149.0449m/z | 149.0449 | 1.53465 | pos | Ribonic acid | HMDB0000867 | Organic oxygen compounds | Organooxygen compounds | Carbohydrates and carbohydrate conjugates | 0.096638 | 0.034336 |
| 14.20_136.1120m/z | 136.112 | 14.19923 | pos | Amphetamine | HMDB0014328 | Benzenoids | Benzene and substituted derivatives | Phenethylamines | 0.293026 | 0.002001 |
| 4.83_335.1717m/z | 335.1717 | 4.826567 | neg | Pyridoxamine | HMDB0001431 | Organoheterocyclic compounds | Pyridines and derivatives | Pyridoxamines | 0.330396 | 0.022646 |
| 6.96_182.0812m/z | 182.0812 | 6.95545 | pos | Enol-phenylpyruvate | HMDB0012225 | Benzenoids | Benzene and substituted derivatives | Phenylpyruvic acid derivatives | 0.149856 | 0.023142 |
| 3.31_198.0761m/z | 198.0761 | 3.314017 | pos | DL-Dopa | HMDB0000609 | Organic acids and derivatives | Carboxylic acids and derivatives | Amino acids, peptides, and analogues | 0.166459 | 0.011452 |
| 4.63_214.0122m/z | 214.0122 | 4.626867 | neg | 2-Amino-3-phosphonopropionic acid | HMDB0000370 | Organic acids and derivatives | Carboxylic acids and derivatives | Amino acids, peptides, and analogues | 0.31861 | 0.004466 |
| 5.00_170.0367m/z | 170.0367 | 5.003433 | pos | 3-Methylindole | HMDB0000466 | Organoheterocyclic compounds | Indoles and derivatives | Indoles | 0.080058 | 0.011328 |
| 6.96_137.1324m/z | 137.1324 | 6.95545 | pos | D-Limonene | HMDB0003375 | Lipids and lipid-like molecules | Prenol lipids | Monoterpenoids | 0.126075 | 0.007699 |
| 7.64_129.0699m/z | 129.0699 | 7.637983 | pos | Naphthalene | HMDB0029751 | Benzenoids | Naphthalenes | Unclassified | 0.088319 | 0.007276 |
| 8.37_359.0968m/z | 359.0968 | 8.372083 | neg | 3-Methoxy-4-hydroxyphenylglycol glucuronide | HMDB0000496 | Organic oxygen compounds | Organooxygen compounds | Carbohydrates and carbohydrate conjugates | 1.817203 | 2.36E-05 |
| 1.36_165.0910m/z | 165.091 | 1.361683 | pos | 4-Isopropylbenzoic acid | HMDB0035268 | Lipids and lipid-like molecules | Prenol lipids | Monoterpenoids | 0.407422 | 0.010079 |
| 14.29_135.1167m/z | 135.1167 | 14.28758 | pos | p-Cymene | HMDB0005805 | Lipids and lipid-like molecules | Prenol lipids | Monoterpenoids | 0.350838 | 0.021622 |
| 4.04_258.9922m/z | 258.9922 | 4.038483 | neg | Caffeic acid 3-sulfate | HMDB0041706 | Phenylpropanoids and polyketides | Cinnamic acids and derivatives | Hydroxycinnamic acids and derivatives | 0.529183 | 0.00225 |
| 4.94_241.0106m/z | 241.0106 | 4.939467 | neg | Myo-inositol 1-phosphate | HMDB0000213 | Organic oxygen compounds | Organooxygen compounds | Alcohols and polyols | 0.737007 | 8.33E-06 |
| 5.29_165.0545m/z | 165.0545 | 5.293067 | pos | 3,4-Dihydroxyhydrocinnamic acid | HMDB0000423 | Phenylpropanoids and polyketides | Phenylpropanoic acids | Unclassified | 0.138461 | 0.009358 |
| 5.33_243.0334m/z | 243.0334 | 5.333783 | neg | 4-phenylbutanic acid-O-sulphate | HMDB0059983 | Benzenoids | Benzene and substituted derivatives | Unclassified | 5.169591 | 0.000113 |
| 1.18_215.1389m/z | 215.1389 | 1.18395 | pos | Metanephrine | HMDB0004063 | Benzenoids | Phenols | Methoxyphenols | 0.170658 | 0.011331 |
| 13.04_743.5460n | 761.5862 | 13.04303 | pos | PE(14:0/22:2(13Z,16Z)) | HMDB0008843 | Lipids and lipid-like molecules | Glycerophospholipids | Glycerophosphoethanolamines | 20.92861 | 0.03035 |
| 3.30_197.0808m/z | 197.0808 | 3.30475 | pos | Homoveratric acid | HMDB0000434 | Benzenoids | Benzene and substituted derivatives | Methoxybenzenes | 0.112253 | 0.033072 |
| 3.48_179.0702m/z | 179.0702 | 3.476383 | pos | 1,2-Dihydroxy-3,4-epoxy-1,2,3,4-tetrahydronaphthalene | HMDB0062273 | Benzenoids | Tetralins | Unclassified | 0.349583 | 2.79E-08 |
| 3.57_195.1129m/z | 195.1129 | 3.5682 | pos | 6-Hydroxypseudooxynicotine | HMDB0240264 | Organic oxygen compounds | Organooxygen compounds | Carbonyl compounds | 0.245863 | 1.17E-05 |
| 0.54_196.0166m/z | 196.0166 | 0.537917 | pos | 1-nitrosonaphthalene | HMDB0062189 | Benzenoids | Naphthalenes | Unclassified | 2.074194 | 3.36E-05 |
| 0.70_120.0656m/z | 120.0656 | 0.6975 | pos | 2-Ketobutyric acid | HMDB0000005 | Organic acids and derivatives | Keto acids and derivatives | Short-chain keto acids and derivatives | 0.651275 | 0.003053 |
| 0.83_558.0045m/z | 558.0045 | 0.830067 | neg | 2,5-Diamino-6-(5'-triphosphoryl-3',4'-trihydroxy-2'-oxopentyl)-amino-4-oxopyrimidine | HMDB0006823 | Organic oxygen compounds | Organooxygen compounds | Carbohydrates and carbohydrate conjugates | 1.141498 | 0.002124 |
| 0.88_172.0401m/z | 172.0401 | 0.882533 | pos | L-Methionine | HMDB0000696 | Organic acids and derivatives | Carboxylic acids and derivatives | Amino acids, peptides, and analogues | 0.111085 | 0.006811 |
| 1.06_129.0183m/z | 129.0183 | 1.055433 | pos | Oxoglutaric acid | HMDB0000208 | Organic acids and derivatives | Keto acids and derivatives | Gamma-keto acids and derivatives | 0.515104 | 0.00159 |
| 14.36_354.3361m/z | 354.3361 | 14.35548 | pos | Docosadienoate (22:2n6) | HMDB0061714 | Lipids and lipid-like molecules | Fatty Acyls | Fatty acids and conjugates | 0.215339 | 0.037316 |
| 5.24_351.0184m/z | 351.0184 | 5.236283 | neg | (-)-Epicatechin sulfate | HMDB0012467 | Phenylpropanoids and polyketides | Flavonoids | Sulfated flavonoids | 0.428174 | 0.005501 |
| 10.74_293.2130m/z | 293.213 | 10.74013 | neg | 9(S)-HPODE | HMDB0006940 | Lipids and lipid-like molecules | Fatty Acyls | Lineolic acids and derivatives | 0.388053 | 0.004341 |
| 3.99_303.0187m/z | 303.0187 | 3.98965 | neg | 5-(3',4',5'-Trihydroxyphenyl)-gamma-valerolactone-4'-O-sulphate | HMDB0059987 | Organic acids and derivatives | Organic sulfuric acids and derivatives | Arylsulfates | 0.475841 | 0.001917 |
| 1.53_219.0775m/z | 219.0775 | 1.52885 | neg | Felbamate | HMDB0015084 | Benzenoids | Benzene and substituted derivatives | Unclassified | 0.185579 | 0.035484 |
| 3.83_179.0700m/z | 179.07 | 3.827567 | pos | 4-Methoxycinnamic acid | HMDB0002040 | Phenylpropanoids and polyketides | Cinnamic acids and derivatives | Cinnamic acids | 0.126934 | 0.00317 |
| 3.83_305.0344m/z | 305.0344 | 3.830983 | neg | 4-Hydroxy-5-(dihydroxyphenyl)-valeric acid-O-sulphate | HMDB0059978 | Benzenoids | Phenols | Benzenediols | 0.281649 | 0.046719 |
| 13.76_269.1619m/z | 269.1619 | 13.76133 | pos | Histidinyl-Isoleucine | HMDB0028888 | Organic acids and derivatives | Carboxylic acids and derivatives | Amino acids, peptides, and analogues | 0.298287 | 0.003263 |
| 4.24_270.1912m/z | 270.1912 | 4.235433 | pos | Arginyl-Isoleucine | HMDB0028712 | Organic acids and derivatives | Carboxylic acids and derivatives | Amino acids, peptides, and analogues | 0.24484 | 0.01035 |
| 1.64_231.0455m/z | 231.0455 | 1.641483 | neg | gamma-Glutamylcysteine | HMDB0001049 | Organic acids and derivatives | Carboxylic acids and derivatives | Amino acids, peptides, and analogues | 0.317892 | 0.027618 |
| 4.84_253.0720m/z | 253.072 | 4.840767 | neg | Arbutin | HMDB0029943 | Organic oxygen compounds | Organooxygen compounds | Carbohydrates and carbohydrate conjugates | 0.240713 | 0.035432 |
| 11.41_151.1116m/z | 151.1116 | 11.41243 | pos | Perillyl aldehyde | HMDB0003647 | Lipids and lipid-like molecules | Prenol lipids | Monoterpenoids | 0.139366 | 0.027391 |
| 4.53_331.1515m/z | 331.1515 | 4.53305 | pos | Tyramine glucuronide | HMDB0010328 | Organic oxygen compounds | Organooxygen compounds | Carbohydrates and carbohydrate conjugates | 0.243167 | 0.005984 |
| 4.63_306.0400n | 611.0726 | 4.626867 | neg | 4-Hydroxy-5-(dihydroxyphenyl)-valeric acid-O-sulphate III | HMDB0059979 | Benzenoids | Phenols | Benzenediols | 0.763869 | 0.002558 |
| 10.69_727.0392m/z | 727.0392 | 10.69345 | neg | 6-Thioinosine-5'-monophosphate | HMDB0060416 | Nucleosides, nucleotides, and analogues | Purine nucleotides | Purine ribonucleotides | 0.53641 | 0.002488 |
| 15.41_392.9990m/z | 392.999 | 15.40553 | neg | dIDP | HMDB0003536 | Nucleosides, nucleotides, and analogues | Purine nucleotides | Purine deoxyribonucleotides | 0.411421 | 0.014267 |
| 2.86_155.1429m/z | 155.1429 | 2.861917 | pos | Alpha-Terpineol | HMDB0004043 | Lipids and lipid-like molecules | Prenol lipids | Monoterpenoids | 0.121691 | 0.005316 |
| 3.66_159.0921m/z | 159.0921 | 3.658167 | neg | Pseudooxynicotine | HMDB0001240 | Organic oxygen compounds | Organooxygen compounds | Carbonyl compounds | 0.046258 | 0.04404 |
| 8.72_195.0803m/z | 195.0803 | 8.720517 | pos | N-Formyl-L-methionine | HMDB0001015 | Organic acids and derivatives | Carboxylic acids and derivatives | Amino acids, peptides, and analogues | 0.123448 | 0.029958 |
| 1.80_143.9967m/z | 143.9967 | 1.7971 | pos | Dimethyl trisulfide | HMDB0013780 | Organosulfur compounds | Organic trisulfides | Unclassified | 2.080667 | 2.53E-06 |
| 10.87_299.1479m/z | 299.1479 | 10.8728 | pos | 4-Hydroxycinnamoylagmatine | HMDB0033460 | Phenylpropanoids and polyketides | Cinnamic acids and derivatives | Hydroxycinnamic acids and derivatives | 0.149083 | 0.018849 |
| 4.46_653.1342m/z | 653.1342 | 4.4562 | pos | CMP-N-glycoloylneuraminate | HMDB0012206 | Nucleosides, nucleotides, and analogues | Pyrimidine nucleotides | Pyrimidine ribonucleotides | 0.749319 | 2.51E-05 |
| 4.85_235.0885m/z | 235.0885 | 4.849867 | neg | Carbamazepine | HMDB0014704 | Organoheterocyclic compounds | Benzazepines | Dibenzazepines | 0.330385 | 0.005371 |
| 13.20_785.6504m/z | 785.6504 | 13.19578 | pos | SM(d18:1/22:1(13Z)) | HMDB0012104 | Lipids and lipid-like molecules | Sphingolipids | Phosphosphingolipids | 3.233246 | 0.015282 |
| 1.58_130.9664m/z | 130.9664 | 1.580383 | pos | trans-3-Chloro-2-propene-1-ol | HMDB0060514 | Organohalogen compounds | Vinyl halides | Vinyl chlorides | 0.792361 | 0.003757 |
| 13.94_801.6827m/z | 801.6827 | 13.93542 | pos | SM(d18:1/23:0) | HMDB0012105 | Lipids and lipid-like molecules | Sphingolipids | Phosphosphingolipids | 1.450571 | 0.006974 |
| 6.07_171.0657m/z | 171.0657 | 6.072417 | neg | Diacetyl | HMDB0003407 | Organic oxygen compounds | Organooxygen compounds | Carbonyl compounds | 0.089942 | 0.035735 |
| 4.97_245.1285m/z | 245.1285 | 4.968167 | pos | Prolylphenylalanine | HMDB0011179 | Organic acids and derivatives | Carboxylic acids and derivatives | Amino acids, peptides, and analogues | 0.233047 | 0.003135 |
| 9.47_125.0961m/z | 125.0961 | 9.471 | pos | 2,3-Octanedione | HMDB0031293 | Organic oxygen compounds | Organooxygen compounds | Carbonyl compounds | 0.529861 | 0.000108 |
